# Supplementary material for: Hybrids of Imatinib with Quinoline: Synthesis, Antimyeloproliferative Activity Evaluation, and Molecular Docking
Source: Pharmaceuticals (Basel). 2022 Mar 3;15(3):309. doi: 10.3390/ph15030309 (PMC8950477; doi:10.3390/ph15030309)
Supplement: Supplementary file 1 [file pharmaceuticals-15-00309-s001.zip › pharmaceuticals-1571646-supplementary.pdf]

# Hybrids of Imatinib with Quinoline: Synthesis, Anti-myeloproliferative Activity Evaluation, and Molecular Docking

Carine Santos[1,2], Luiz Pimentel[1], Henayle Canzian[1], Andressa Oliveira[1,2], Floriano Junior[3], Rafael Dantas[3], Lucas Hoelz[1], Debora Marinho[1], Anna Cunha[4], Monica M. Bastos[1,2], Nubia Boechat*[1,2]

[1] Laboratório de Sintese de Farmacos - LASFAR, Instituto de Tecnologia em Farmacos - Farmanguinhos, FIOCRUZ, Rua Sizenando Nabuco 100, Manguinhos, Rio de Janeiro, RJ, 21041-250, Brazil.
[2] Programa de Pós-graduação em Farmacologia e Química Medicinal do Instituto de Ciências Biomédicas – ICB- UFRJ, Centro de Ciências da Saúde - CCS, Bloco J, Ilha do Fundão, Rio de Janeiro, RJ, 21941-902, Brazil.
[3] Laboratório de Bioquímica Experimental e Computacional de Fármacos, Instituto Oswaldo Cruz, FIOCRUZ, Av. Brasil 4365, Manguinhos, Rio de Janeiro, RJ, 21040-360, Brazil.
[4] Departamento de Química Orgânica, Universidade Federal Fluminense – UFF, Campus do Valonguinho, Niterói, RJ, CEP 24020-150, Brazil.
* Nubia Boechat,, nboechat@gmail.com

## Table of contents (IR, HRMS, HPLC-UV, $^{1}$H NMR, $^{13}$C NMR, $^{19}$F NMR):

Compounds **2a-h**........................................................................ Page 2-50

Compounds **3a-b**........................................................................ Page 51-62

Compounds **4a-b**........................................................................ Page 63-74

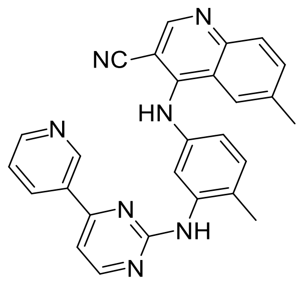


**2a**

6-methyl-4-((4-methyl-3-((4-(pyridin-3-yl)pyrimidin-2-yl)amino)phenyl)amino)quinoline-3-carbonitrile

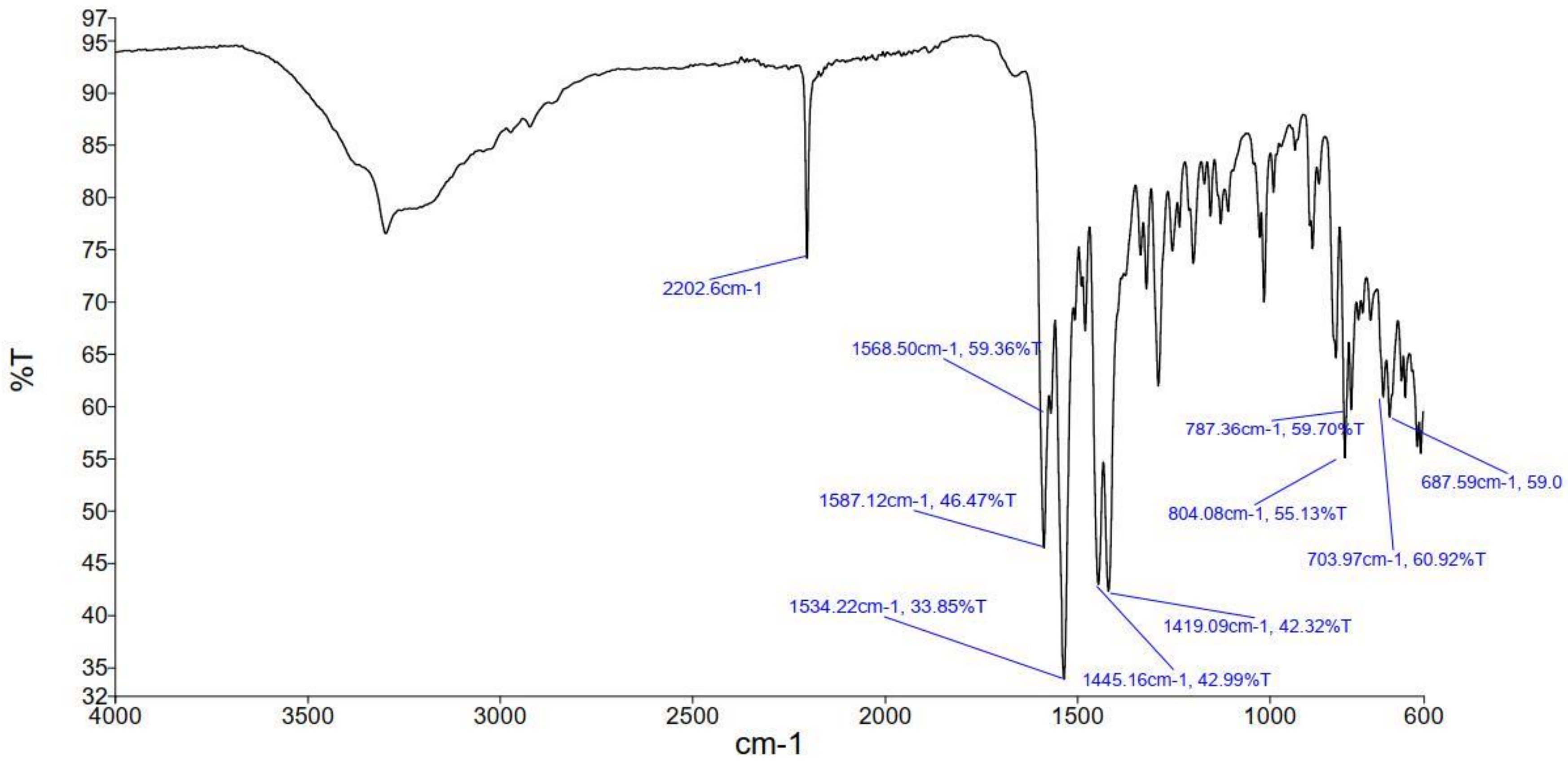


**Figure S1.** IR of **2a**

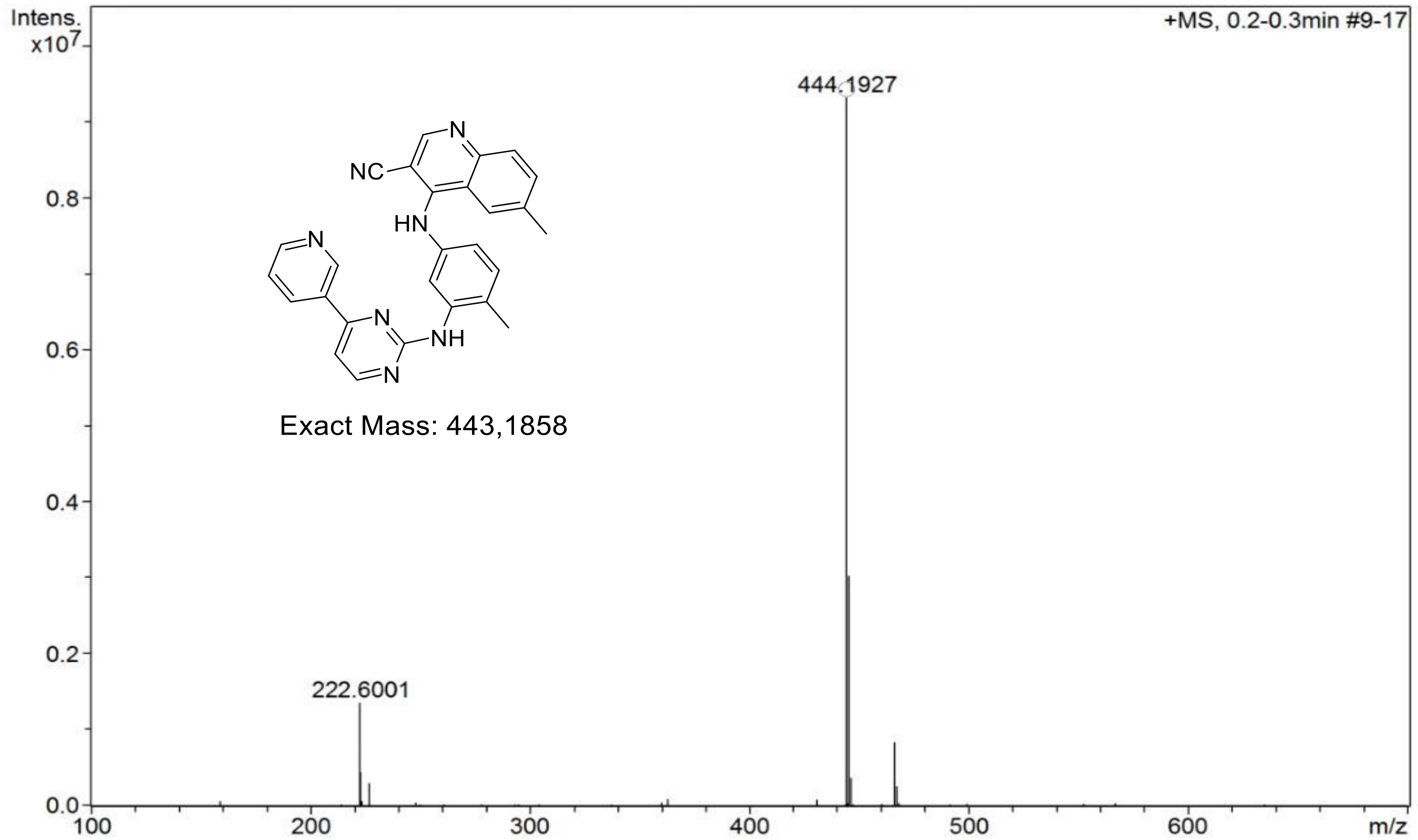


| Meas. m/z | # | Ion Formula | m/z | err [ppm] | Mean err [ppm] | rdb | N-Rule | e⁻ Conf | mSigma | Std I | Std Mean m/z | Std I VarNorm | Std m/z Diff | Std Comb Dev |
|---|---|---|---|---|---|---|---|---|---|---|---|---|---|---|
| 444.192650 | 1 | C27H22N7 | 444.193120 | 1.1 | 0.8 | 20.5 | ok | even | 6.3 | 8.7 | n.a. | n.a. | n.a. | n.a. |
| | 2 | C26H26N3O4 | 444.191783 | -2.0 | -1.9 | 15.5 | ok | even | 17.5 | 22.0 | n.a. | n.a. | n.a. | n.a. |

**Figure S2.** HRMS of **2a**

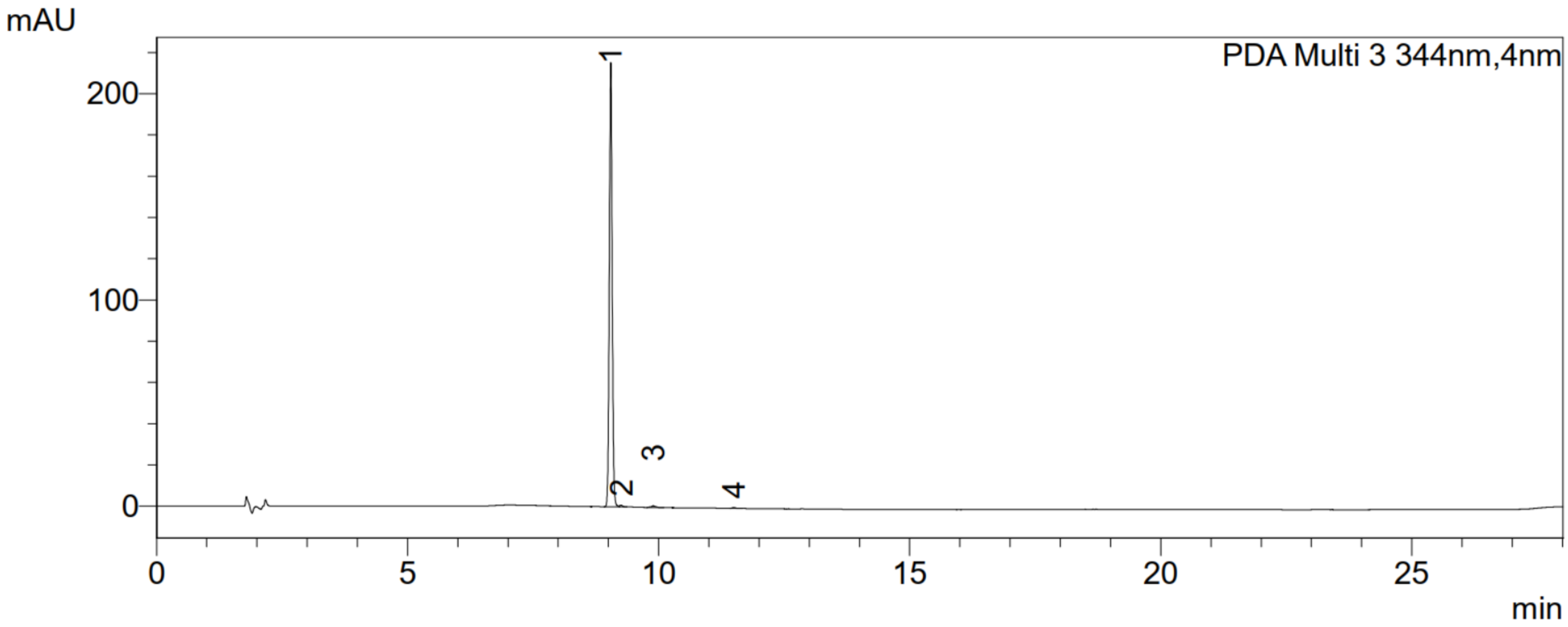


PDA Ch3 344nm

| Peak# | Ret. Time | Name | Area | Area% | Theoretical Plates/meter(USP) | Tailing Factor | Resolution(USP) | Capacity Factor(k') |
|---|---|---|---|---|---|---|---|---|
| 1 | 9,05 | | 855907 | 98,9 | 776688 | 1,033 | -- | -- |
| 2 | 9,26 | | 2890 | 0,3 | 557573 | -- | 1,857 | 0,024 |
| 3 | 9,89 | | 4959 | 0,6 | 497826 | 1,045 | 4,603 | 0,093 |
| 4 | 11,50 | | 1677 | 0,2 | 967519 | 1,329 | 12,088 | 0,271 |
| Total | | | 865432 | 100,0 | | | | |

**Figure S3.** HPLC-UV of **2a.**

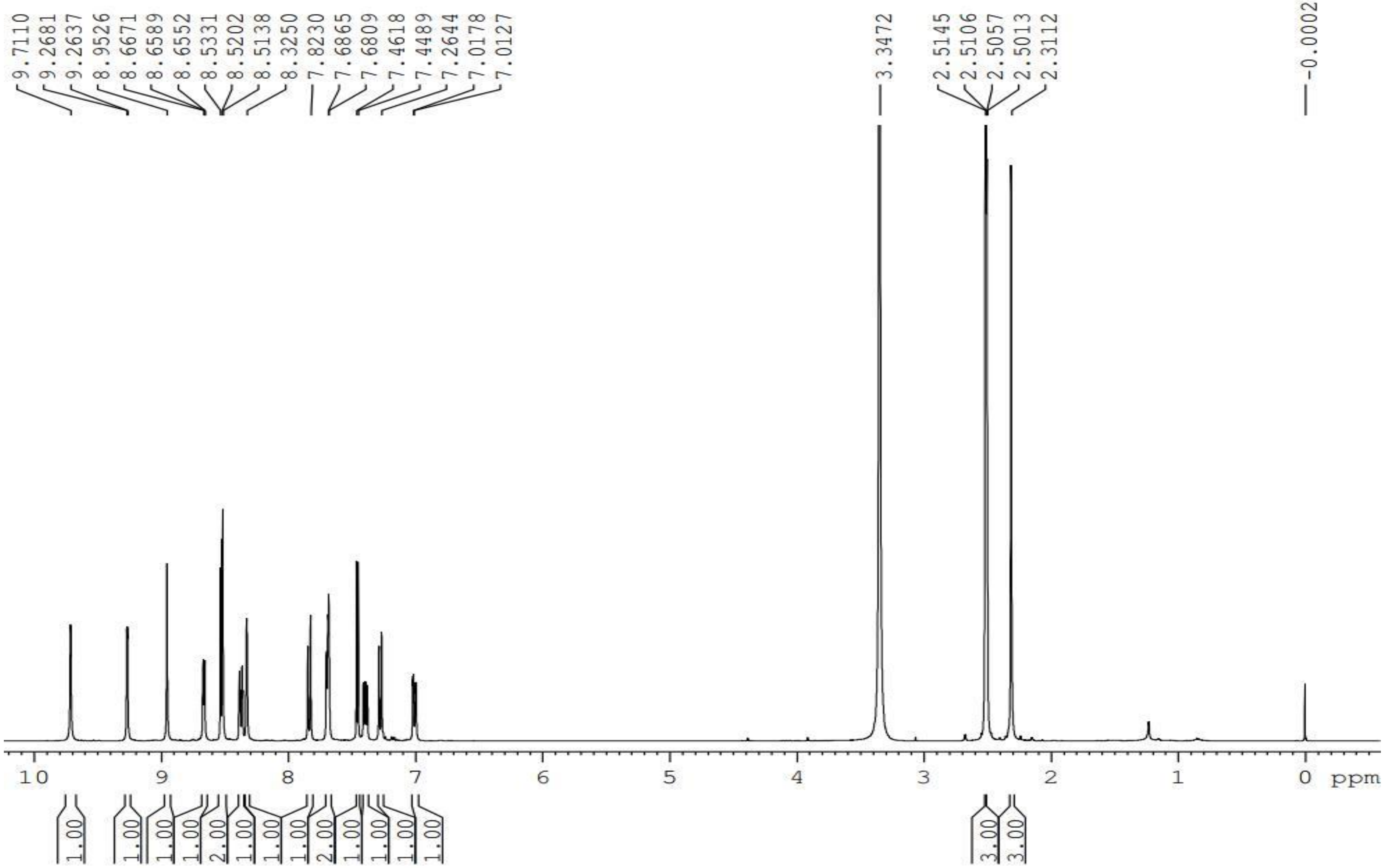


**Figure S4.** $^{1}H$ NMR of **2a**

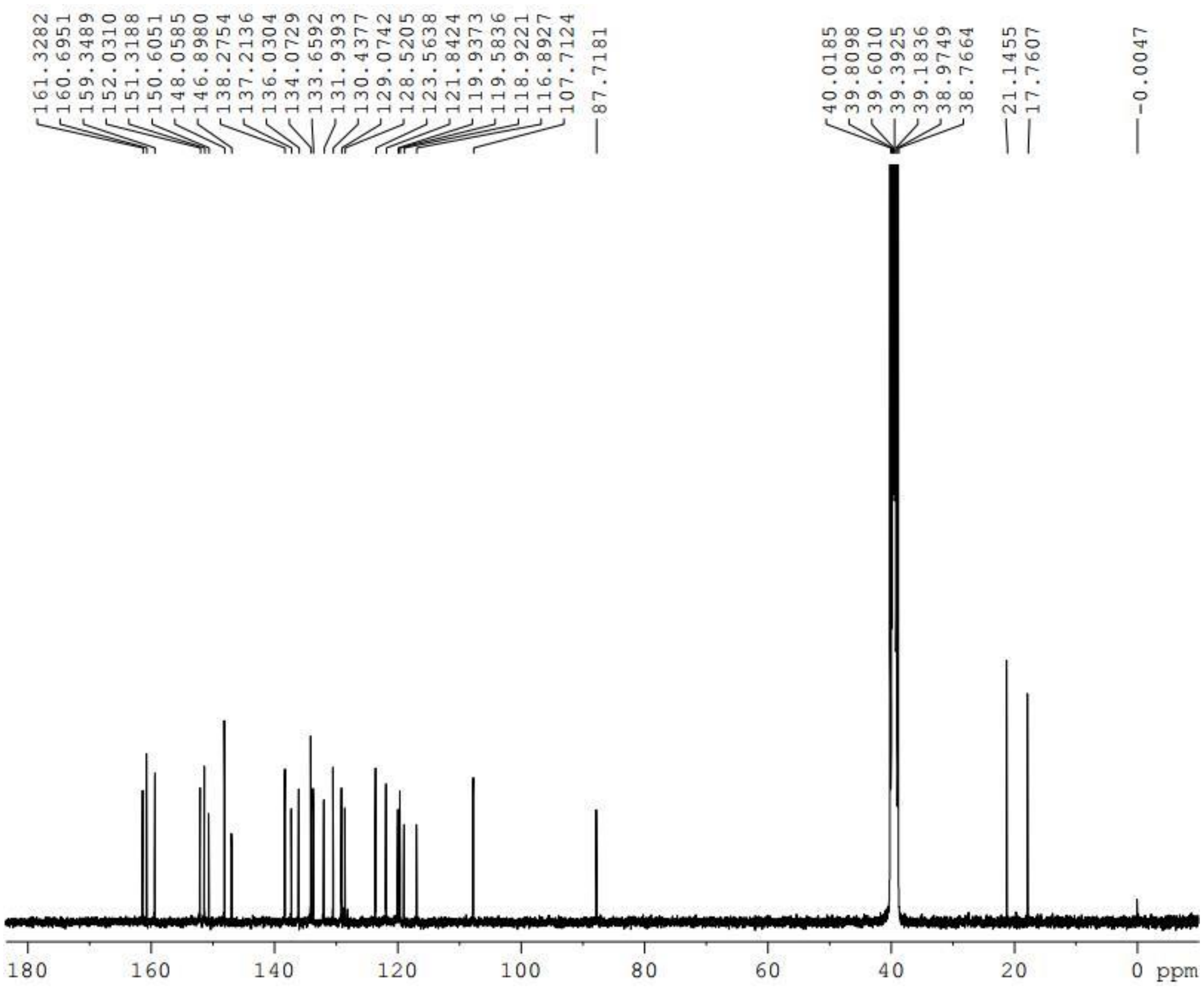


**Figure S5.** $^{13}C$ NMR of **2a**

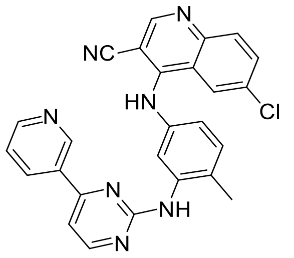


**2b**

6-chloro-4-((4-methyl-3-((4-(pyridin-3-yl)pyrimidin-2-yl)amino)phenyl)amino)quinoline-3-carbonitrile

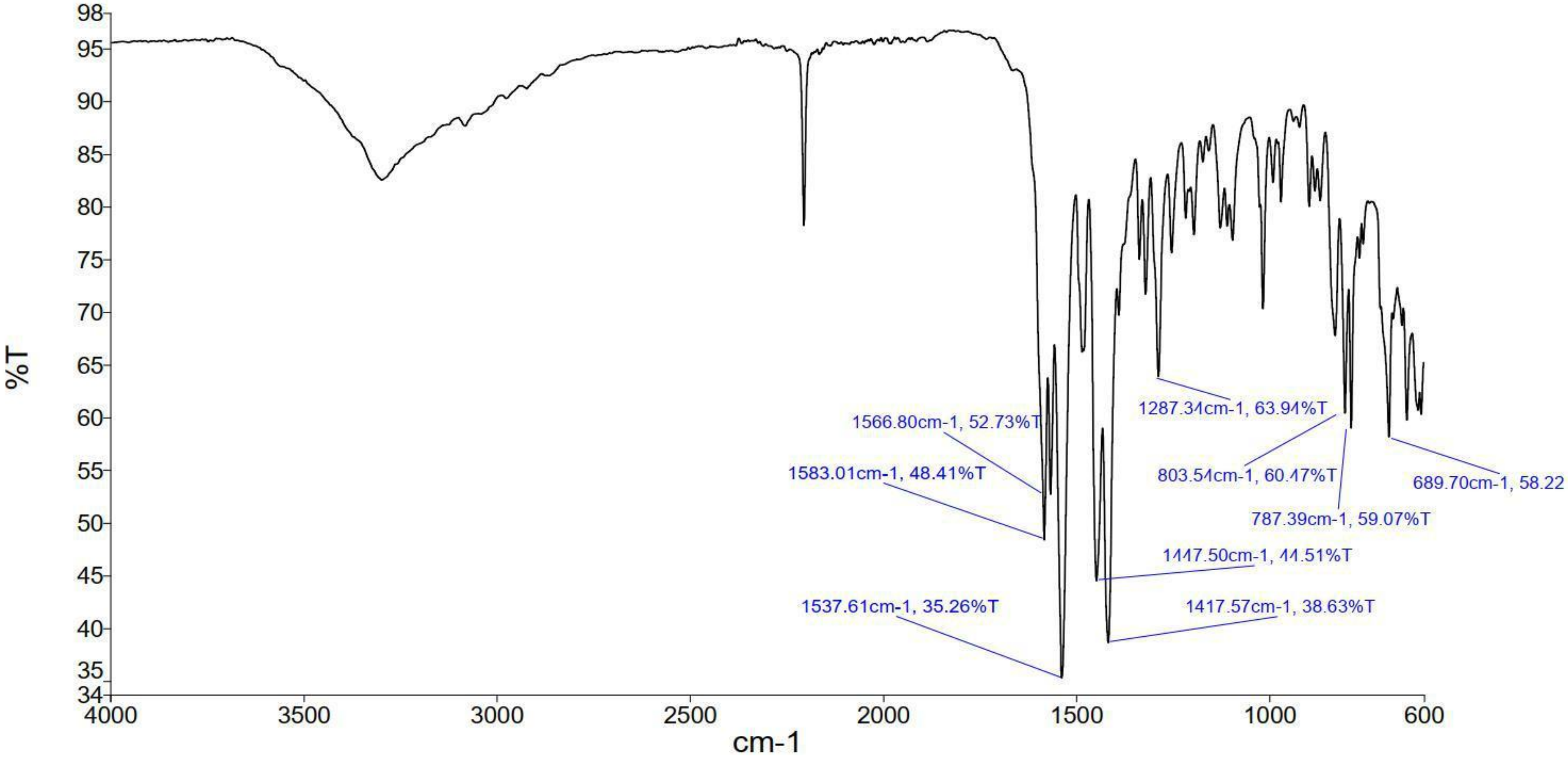


**Figure S6.** IR of **2b**

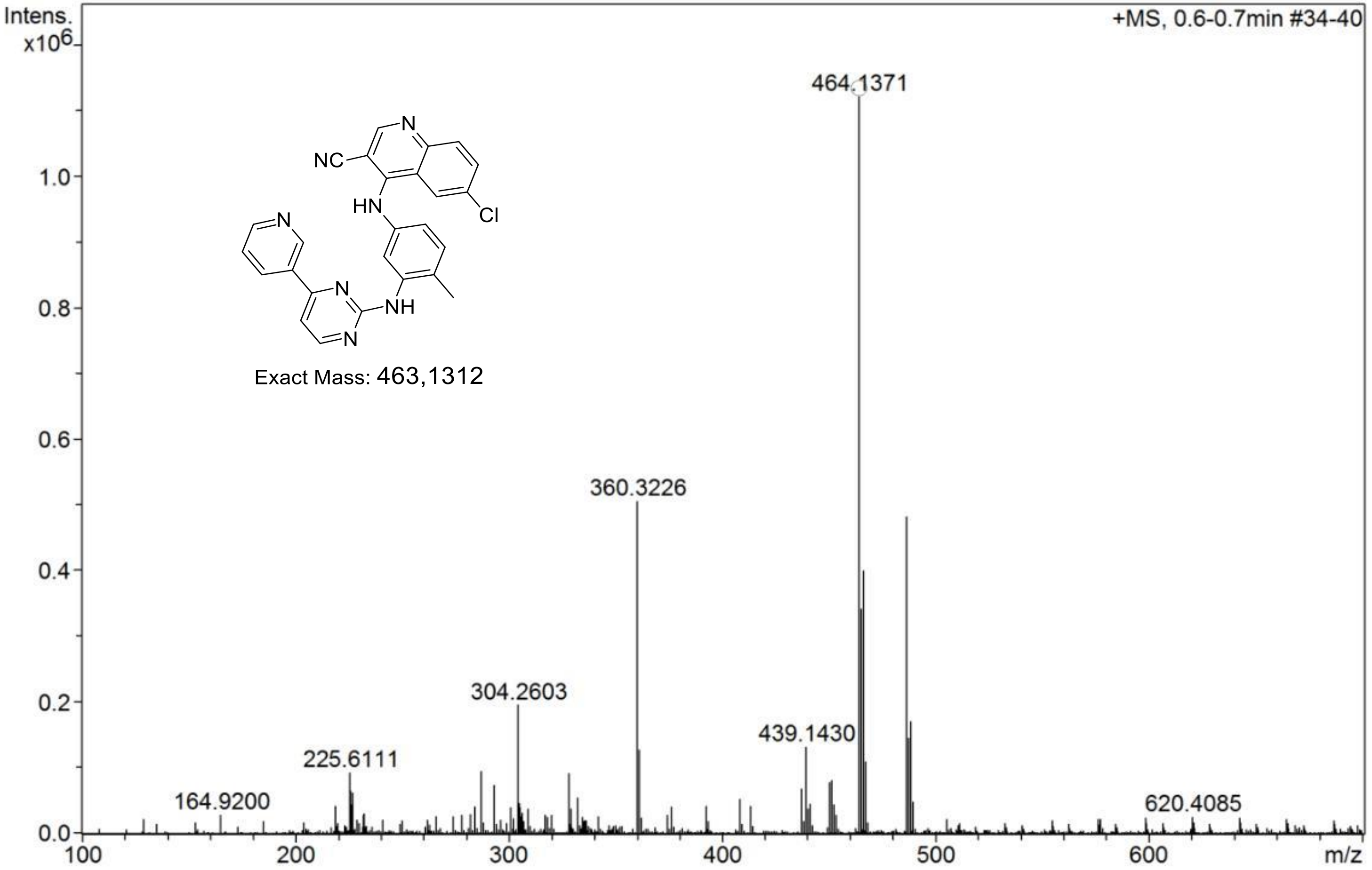


| Meas. m/z | # | Ion Formula | m/z | err [ppm] | Mean err [ppm] | rdb | N-Rule | e⁻ Conf | mSigma | Std I | Std Mean m/z | Std I VarNorm | Std m/z Diff | Std Comb Dev |
|---|---|---|---|---|---|---|---|---|---|---|---|---|---|---|
| 464.137103 | 1 | C26H19ClN7 | 464.138498 | 3.0 | 3.1 | 20.5 | ok | even | 5.1 | 7.1 | n.a. | n.a. | n.a. | n.a. |
| | 2 | C21H19ClN9O2 | 464.134475 | -5.7 | -6.0 | 16.5 | ok | even | 19.4 | 27.2 | n.a. | n.a. | n.a. | n.a. |

**Figure S7.** HRMS of **2b**

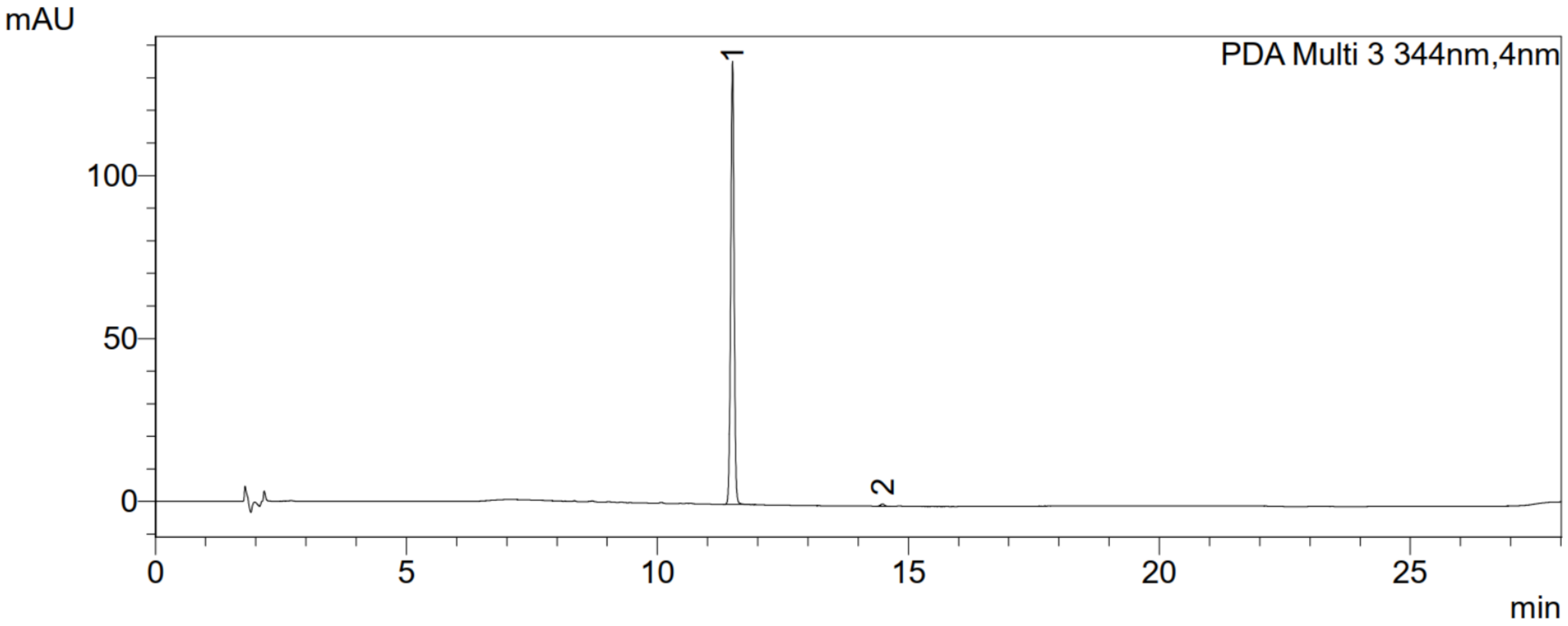


PDA Ch3 344nm

| Peak# | Ret. Time | Name | Area | Area% | Theoretical Plates/meter(USP) | Tailing Factor | Resolution(USP) | Capacity Factor(k') |
|---|---|---|---|---|---|---|---|---|
| 1 | 11,50 | | 636054 | 99,4 | 906890 | 0,980 | -- | -- |
| 2 | 14,49 | | 3673 | 0,6 | 1181877 | 0,946 | 22,804 | 0,260 |
| Total | | | 639727 | 100,0 | | | | |

**Figure S8.** HPLC-UV of **2b**

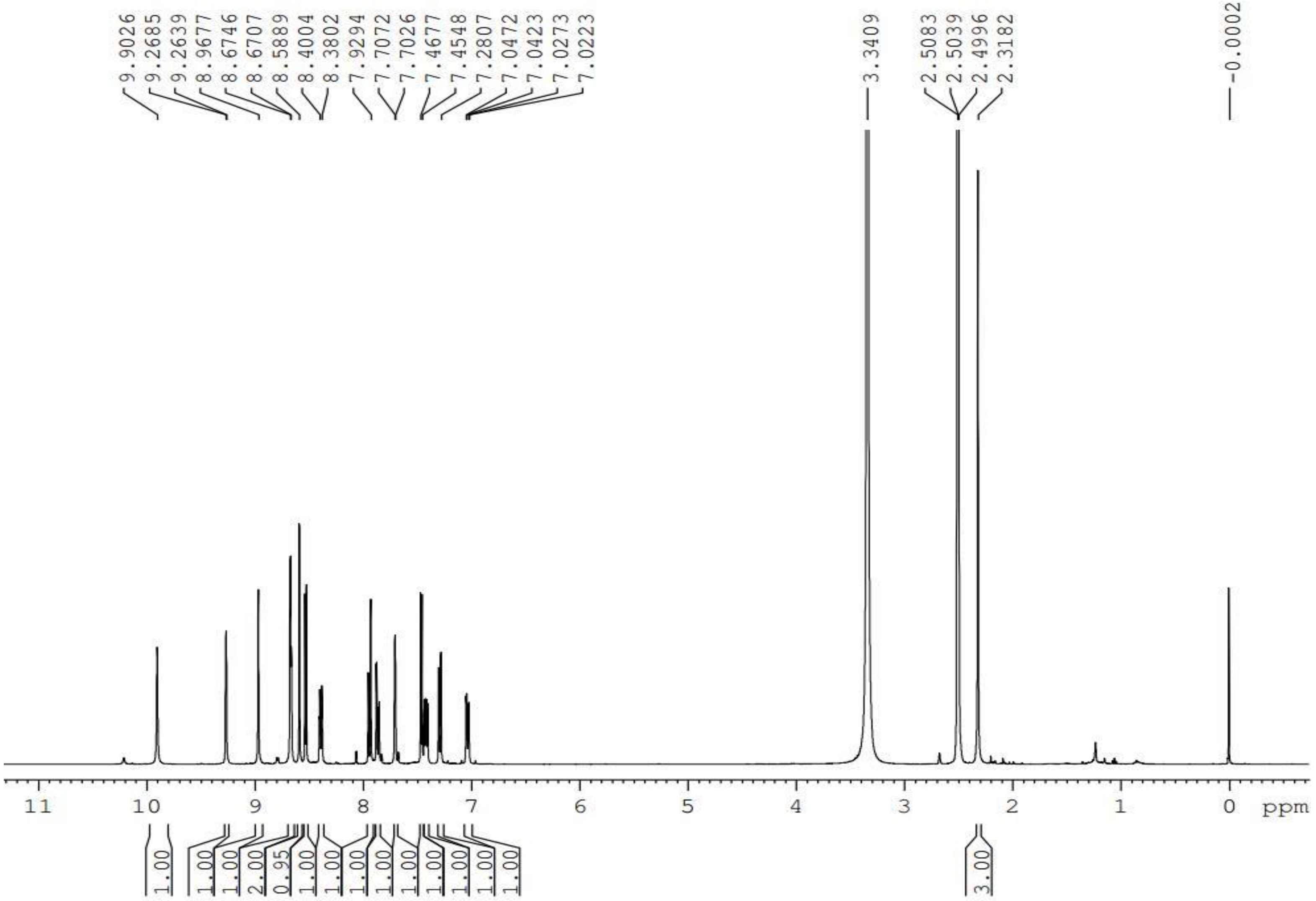


**Figure S9.** $^{1}$H NMR of **2b**

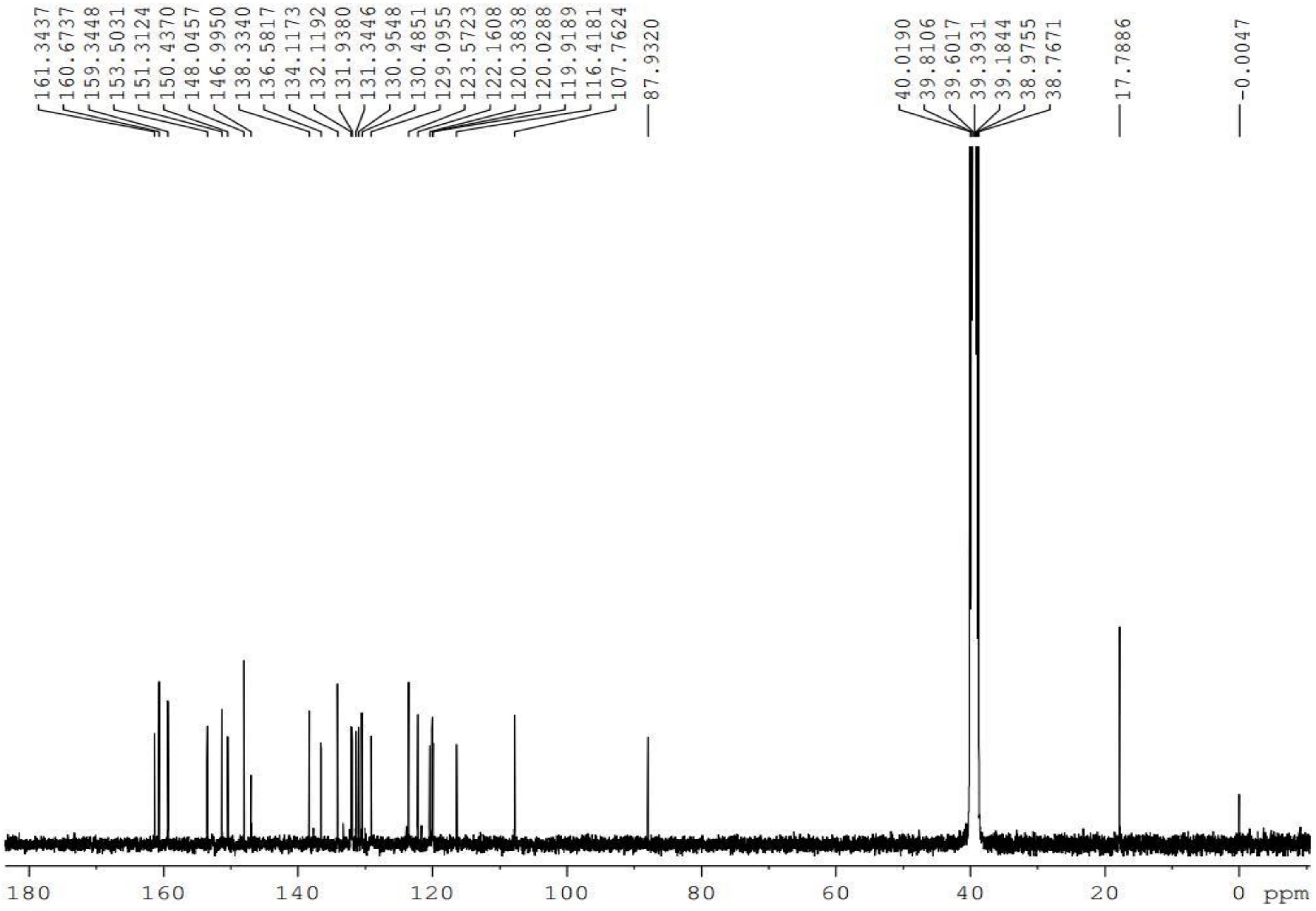


**Figure S10.** $^{13}C$ NMR of **2b**

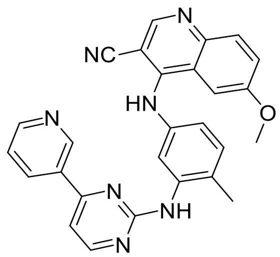


**2c**

6-methoxy-4-((4-methyl-3-((4-(pyridin-3-yl)pyrimidin-2-yl)amino)phenyl)amino)quinoline-3-carbonitrile

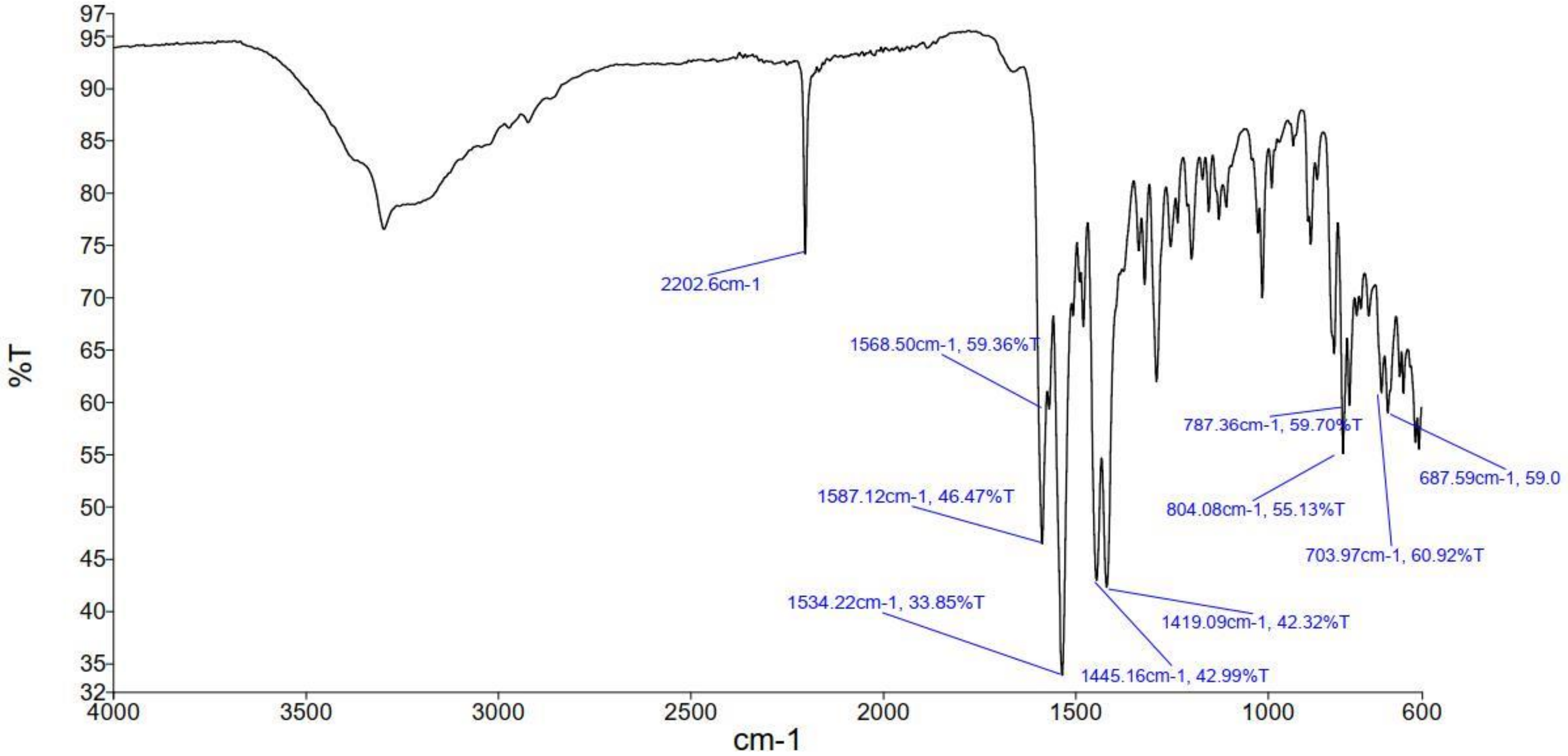


**Figure S11.** IR of **2c**

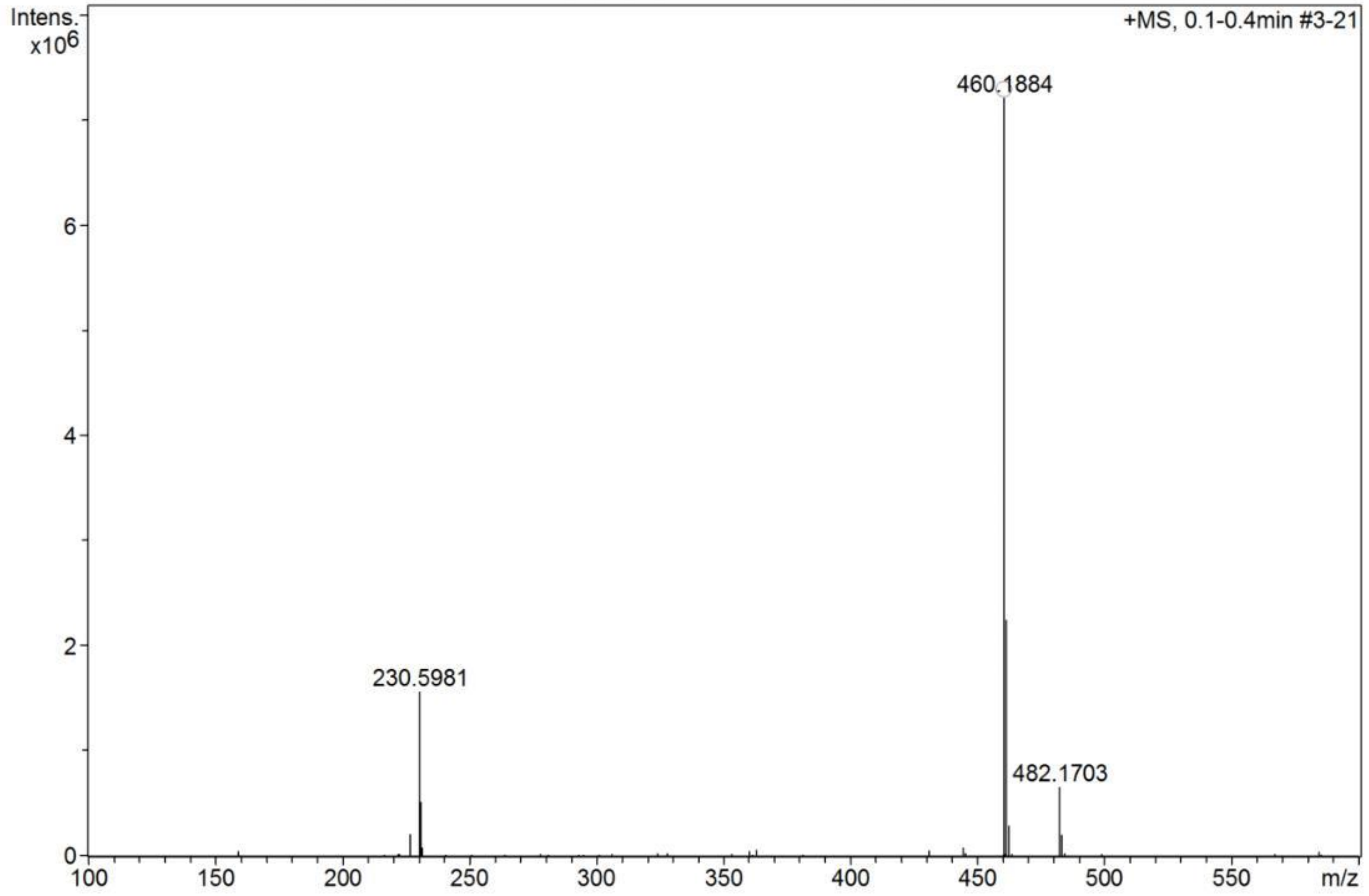


| Meas. m/z | # | Ion Formula | m/z | err [ppm] | Mean err [ppm] | rdb | N-Rule | e$^-$ Conf | mSigma | Std I | Std Mean m/z | Std I VarNorm | Std m/z Diff | Std Comb Dev |
|---|---|---|---|---|---|---|---|---|---|---|---|---|---|---|
| 460.188355 | 1 | $C_{27}H_{22}N_7O$ | 460.188035 | -0.7 | -0.8 | 20.5 | ok | even | 8.2 | 13.5 | n.a. | n.a. | n.a. | n.a. |
| | 2 | $C_{26}H_{26}N_3O_5$ | 460.186697 | -3.6 | -2.0 | 15.5 | ok | even | 9.8 | 14.7 | n.a. | n.a. | n.a. | n.a. |

**Figure S12.** HRMS of **2c**

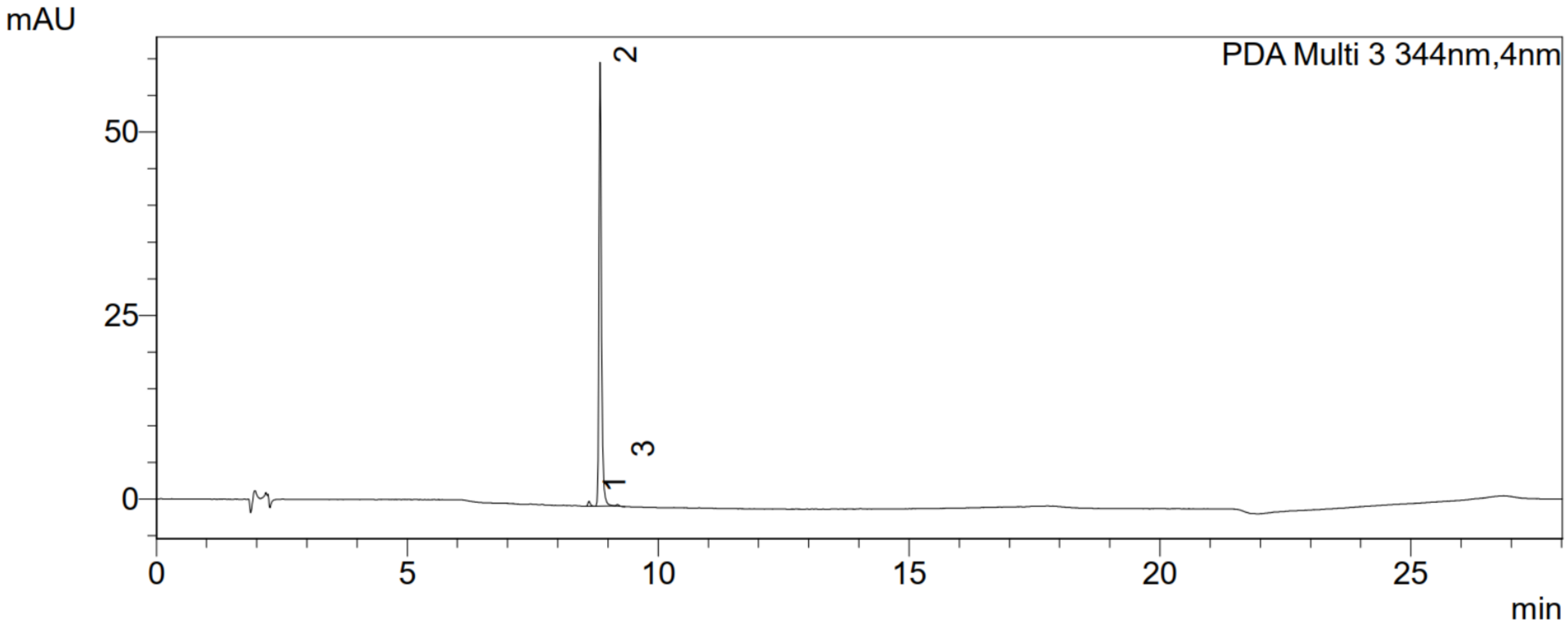


PDA Ch3 344nm

| Peak# | Ret. Time | Name | Area | Area% | Theoretical Plates/meter(USP) | Tailing Factor | Resolution(USP) | Capacity Factor(k') |
|---|---|---|---|---|---|---|---|---|
| 1 | 8,62 | | 2306 | 1,1 | 757420 | 1,237 | -- | -- |
| 2 | 8,84 | | 208720 | 98,6 | 810792 | 1,294 | 2,161 | 0,026 |
| 3 | 9,19 | | 695 | 0,3 | 816422 | 1,313 | 3,348 | 0,066 |
| Total | | | 211721 | 100,0 | | | | |

**Figure S13.** HPLC-UV of **2c**

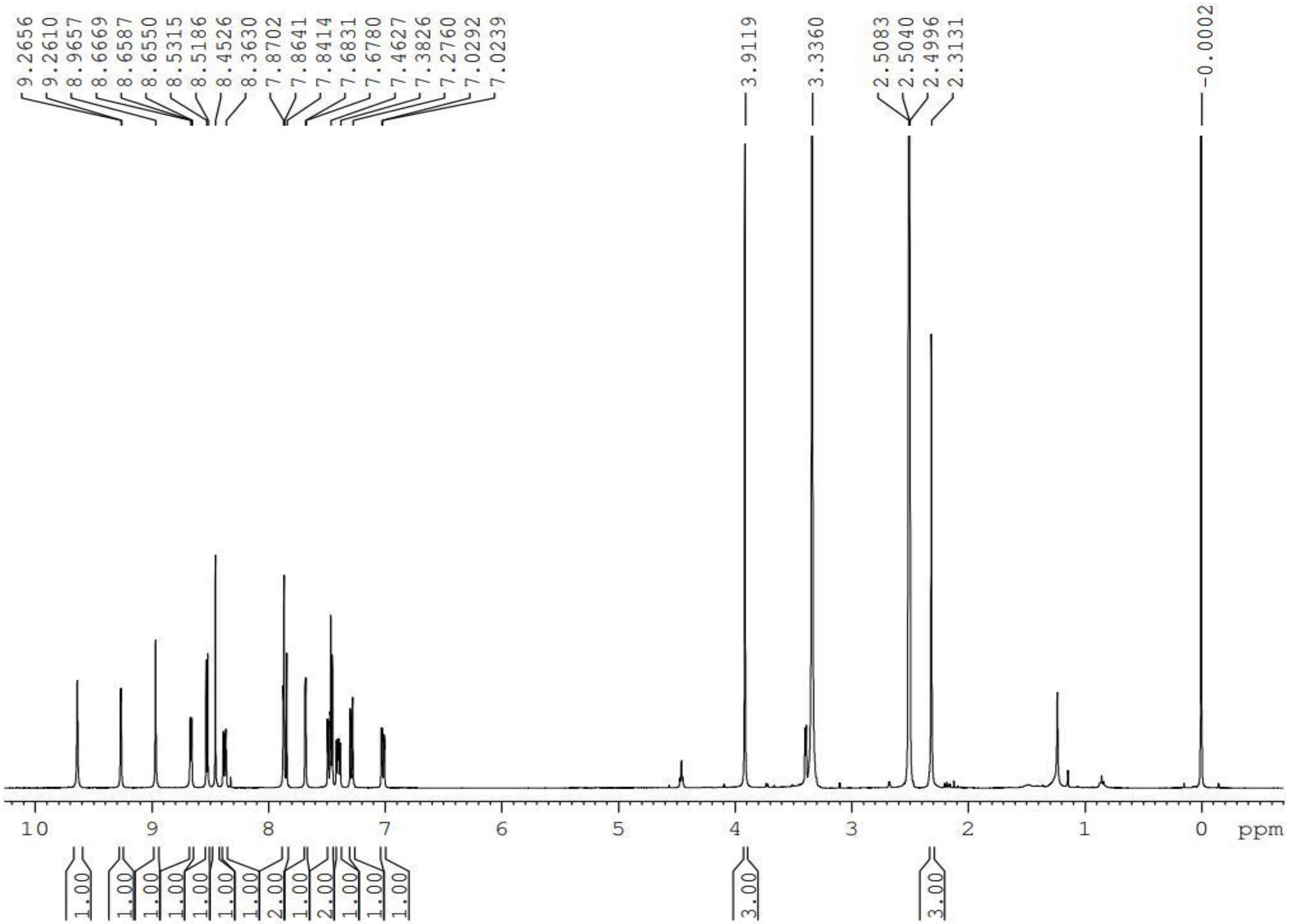


**Figure S14.** $^{1}H$ NMR of **2c**

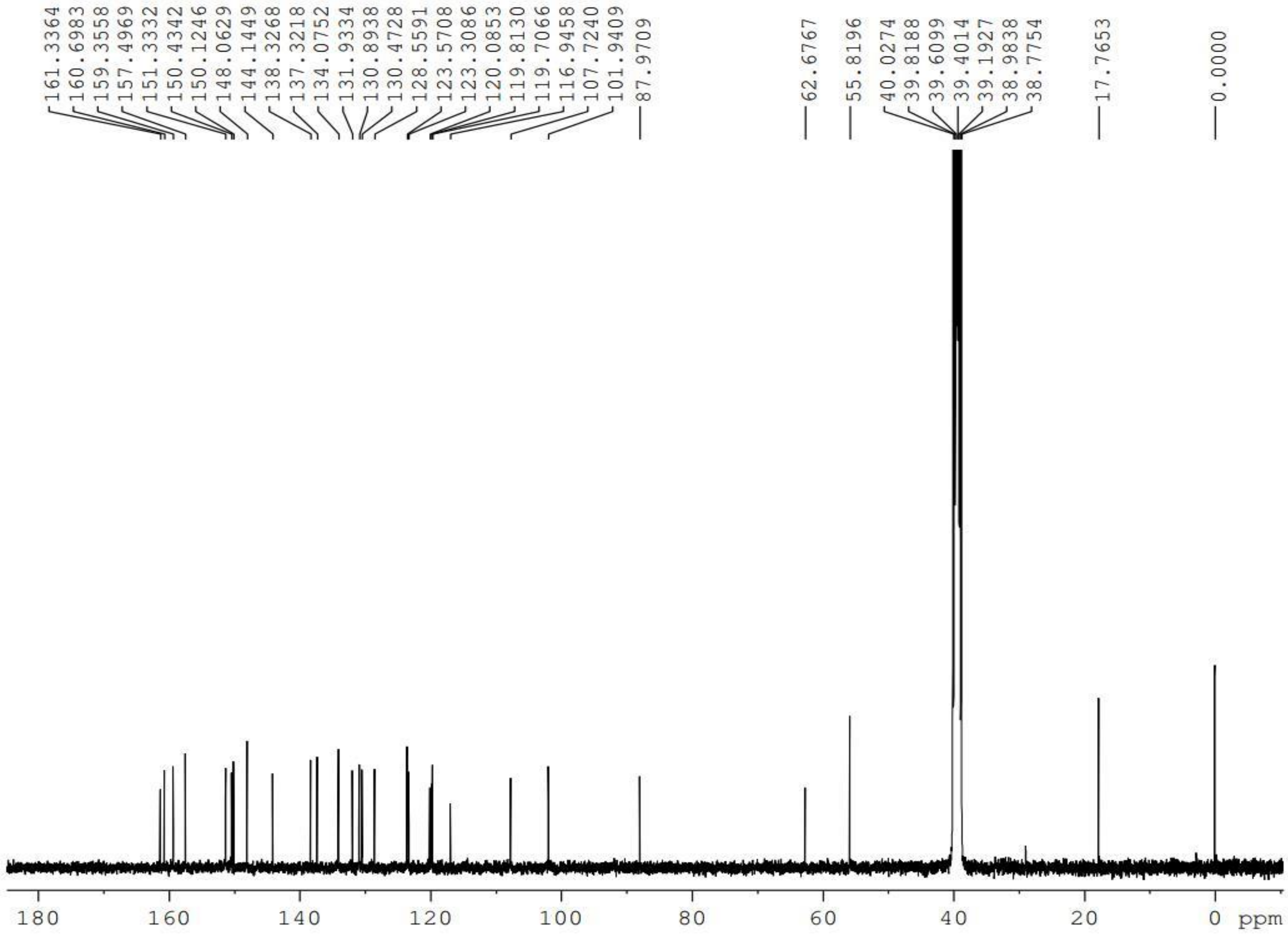


**Figure S15.** $^{13}C$ NMR of **2c**

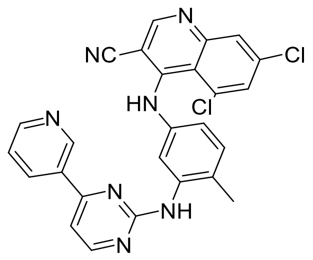


**2d**

5,7-dichloro-4-((4-methyl-3-((4-(pyridin-3-yl)pyrimidin-2-yl)amino)phenyl)amino)quinoline-3-carbonitrile

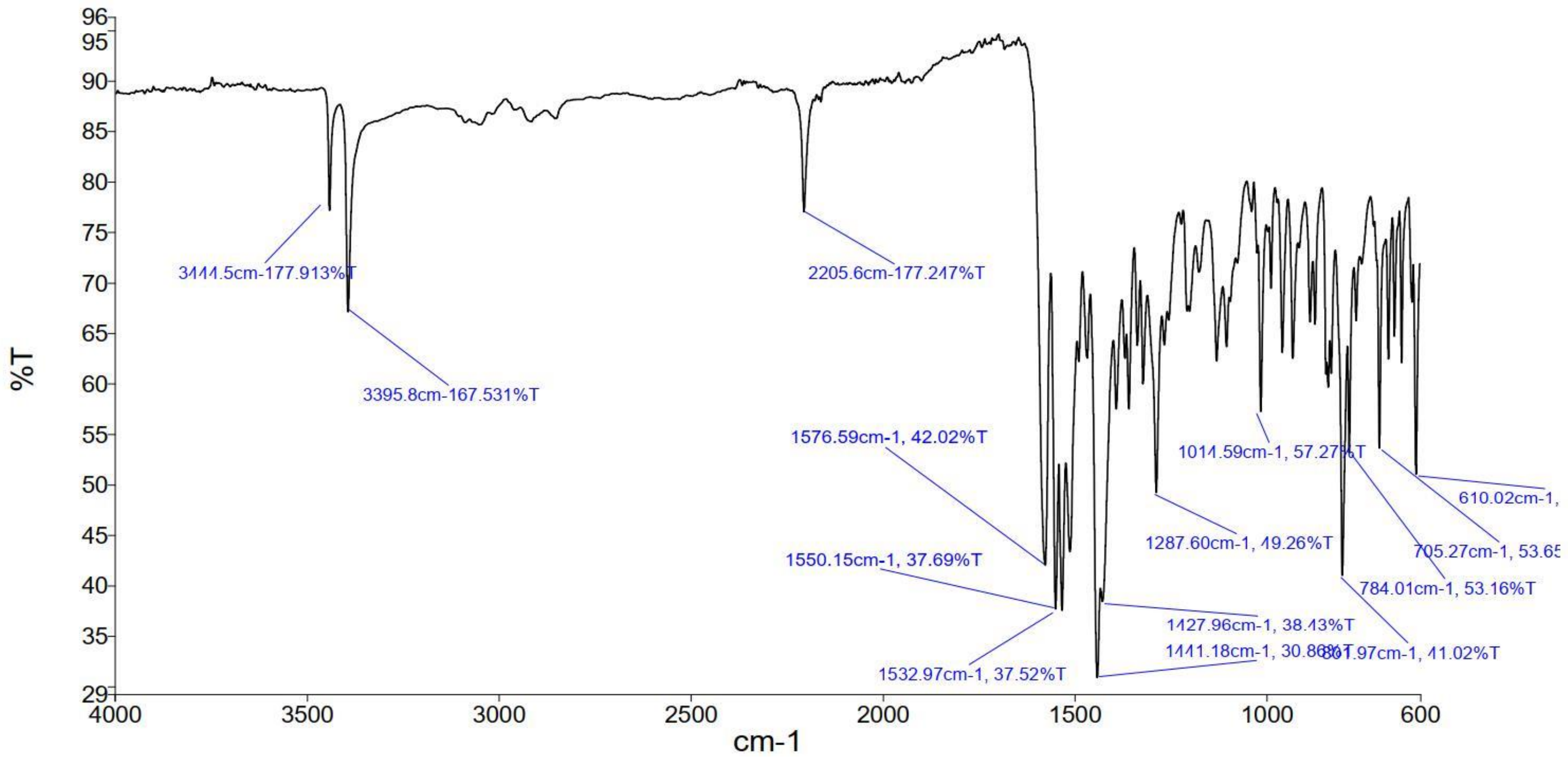


**Figure S16.** IR of **2d**

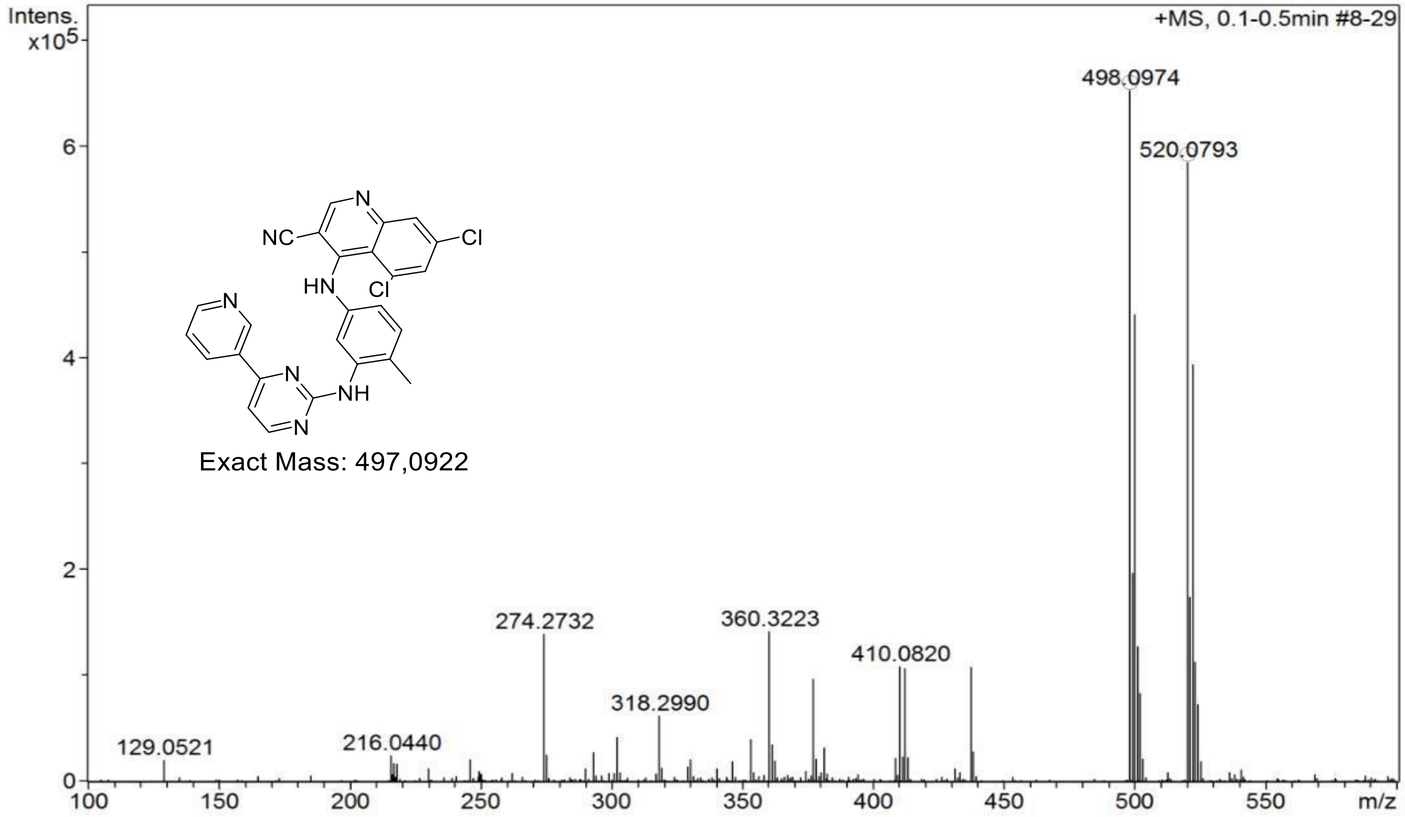


| Meas. m/z | # | Ion Formula | m/z | err [ppm] | Mean err [ppm] | rdb | N-Rule | e⁻ Conf | mSigma | Std I | Std Mean m/z | Std I VarNorm | Std m/z Diff | Std Comb Dev |
|---|---|---|---|---|---|---|---|---|---|---|---|---|---|---|
| 498.097398 | 1 | C26H18Cl2N7 | 498.099525 | 4.3 | 4.5 | 20.5 | ok | even | 6.2 | 6.1 | n.a. | n.a. | n.a. | n.a. |
| | 2 | C25H22Cl2N3O4 | 498.098188 | 1.6 | 2.1 | 15.5 | ok | even | 9.2 | 9.9 | n.a. | n.a. | n.a. | n.a. |
| | 3 | C21H18Cl2N9O2 | 498.095503 | -3.8 | -4.0 | 16.5 | ok | even | 18.8 | 22.1 | n.a. | n.a. | n.a. | n.a. |
| 520.079254 | 1 | C26H17Cl2N7Na | 520.081470 | 4.3 | 4.5 | 20.5 | ok | even | 8.3 | 8.6 | n.a. | n.a. | n.a. | n.a. |
| | 2 | C25H21Cl2N3NaO4 | 520.080132 | 1.7 | 2.3 | 15.5 | ok | even | 8.7 | 9.0 | n.a. | n.a. | n.a. | n.a. |
| | 3 | C21H17Cl2N9NaO2 | 520.077447 | -3.5 | -3.6 | 16.5 | ok | even | 17.0 | 19.9 | n.a. | n.a. | n.a. | n.a. |

**Figure S17.** HRMS of **2d**

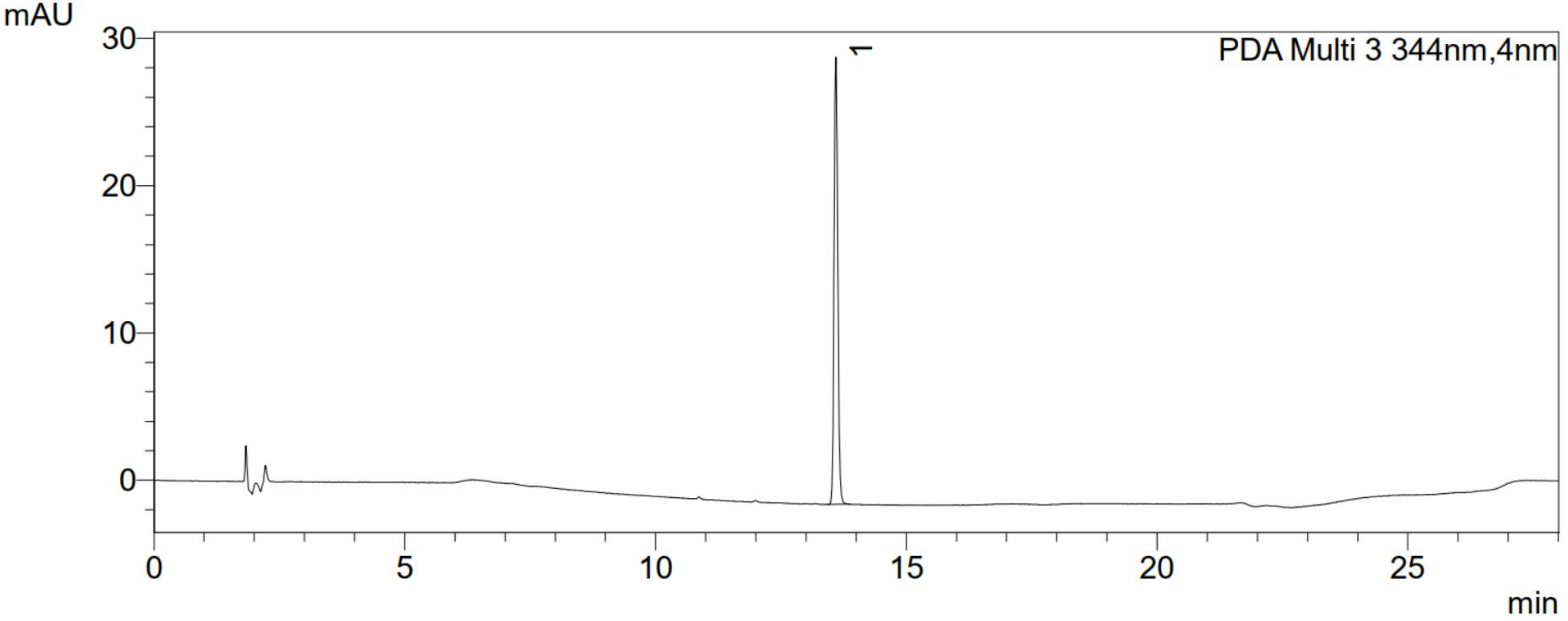


PDA Ch3 344nm

| Peak# | Ret. Time | Name | Area | Area% | Theoretical Plates/meter(USP) | Tailing Factor | Resolution(USP) | Capacity Factor(k') |
|---|---|---|---|---|---|---|---|---|
| 1 | 13,59 | | 157598 | 100,0 | 822150 | 1,114 | -- | -- |
| Total | | | 157598 | 100,0 | | | | |

**Figure S18.** HPLC-UV of **2d**

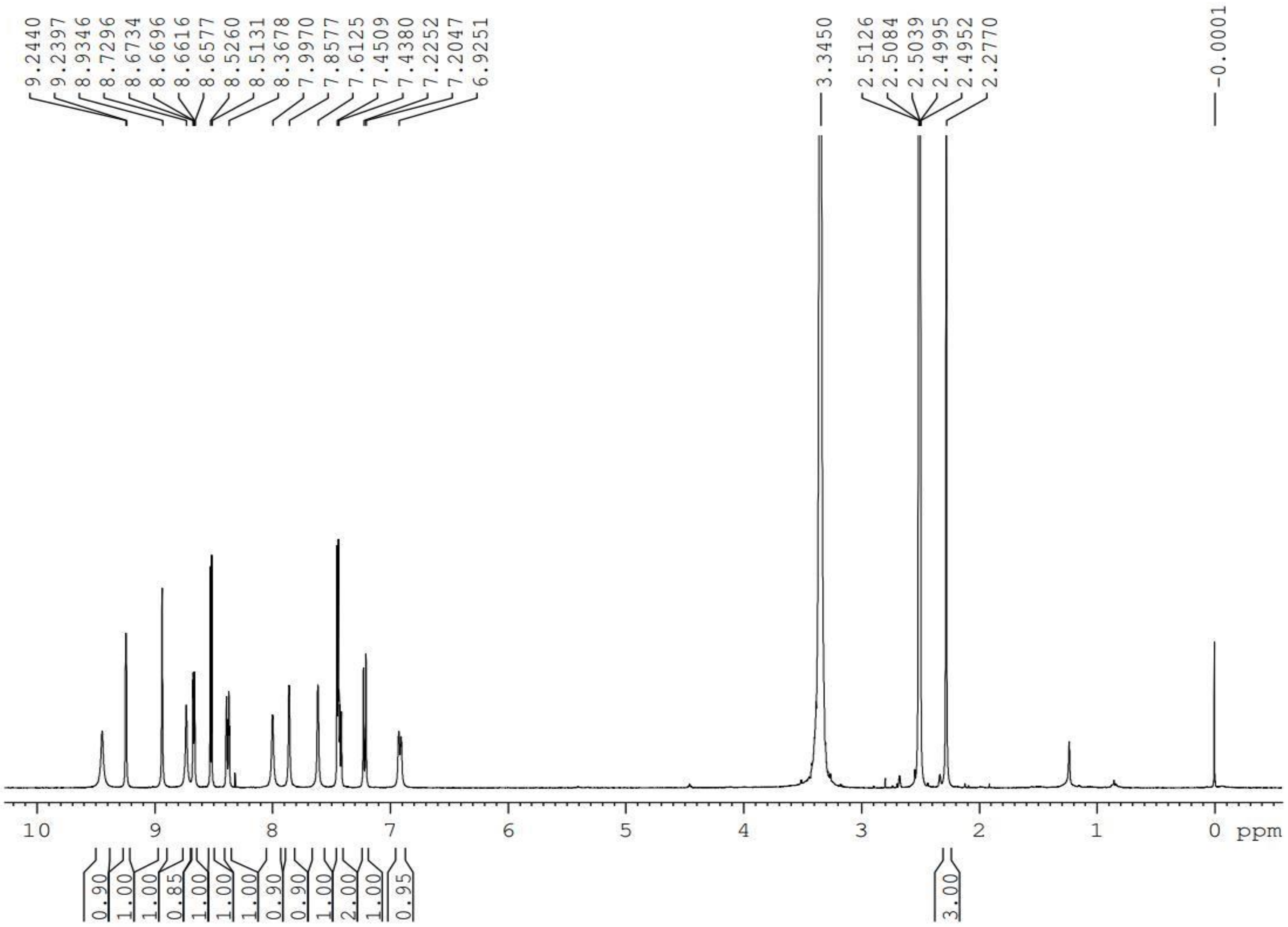

Figure S19. $^{1}$H NMR of **2d**

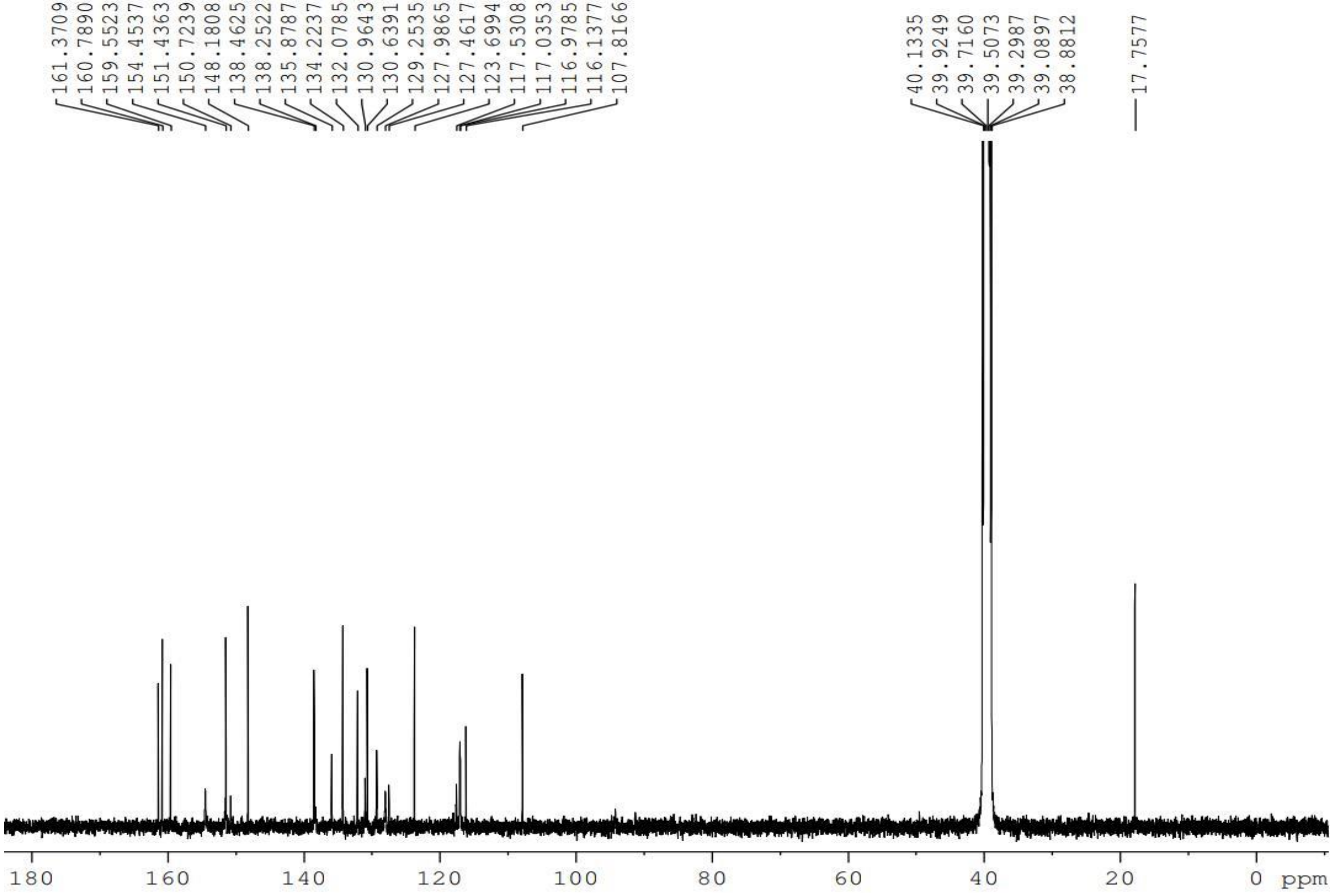


**Figure S20.** $^{13}C$ NMR of **2d**

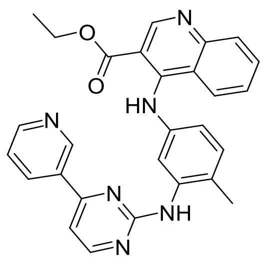

**2e**

ethyl 4-((4-methyl-3-((4-(pyridin-3-yl)pyrimidin-2-yl)amino)phenyl)amino)quinoline-3-carboxylate

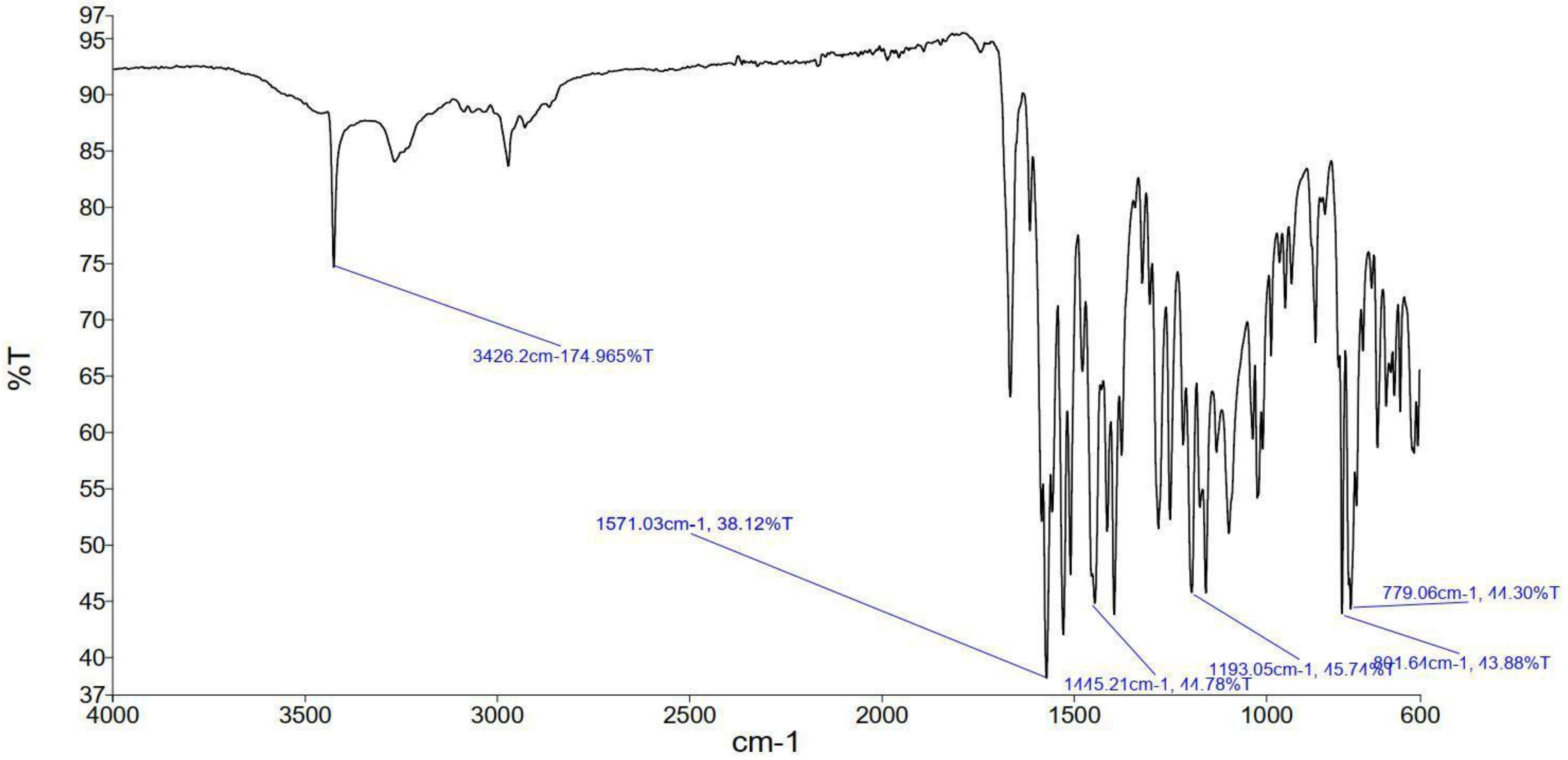


**Figure S21.** IR of **2e**

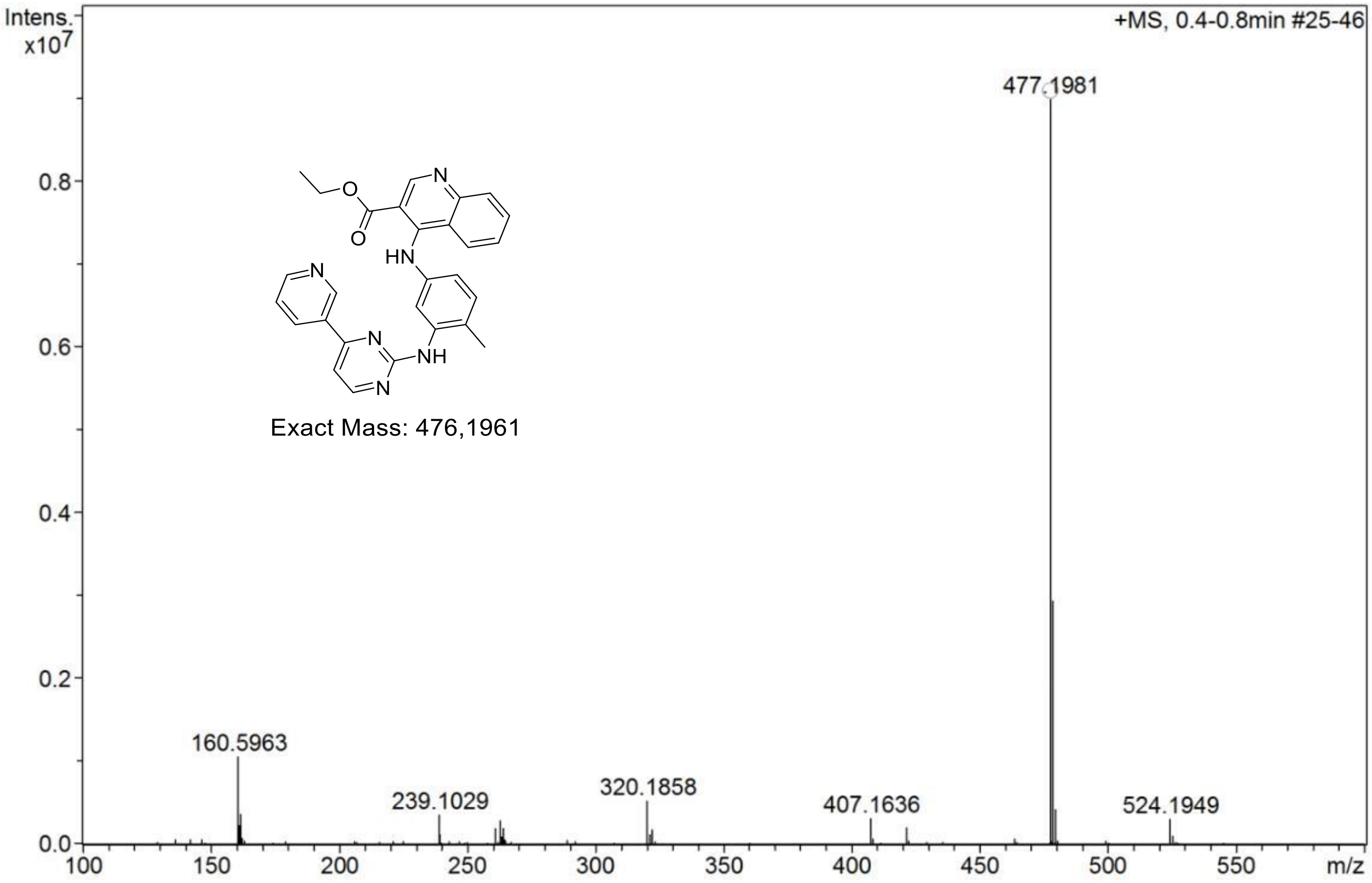


| Meas. m/z | # | Ion Formula | m/z | err [ppm] | Mean err [ppm] | rdb | N-Rule | e⁻ Conf | mSigma | Std I | Std Mean m/z | Std I VarNorm | Std m/z Diff | Std Comb Dev |
|---|---|---|---|---|---|---|---|---|---|---|---|---|---|---|
| 477.198111 | 1 | $C_{23}H_{25}N_8O_4$ | 477.199328 | 2.5 | 2.3 | 15.5 | ok | even | 25.8 | 35.6 | n.a. | n.a. | n.a. | n.a. |

**Figure S22.** HRMS of **2e**

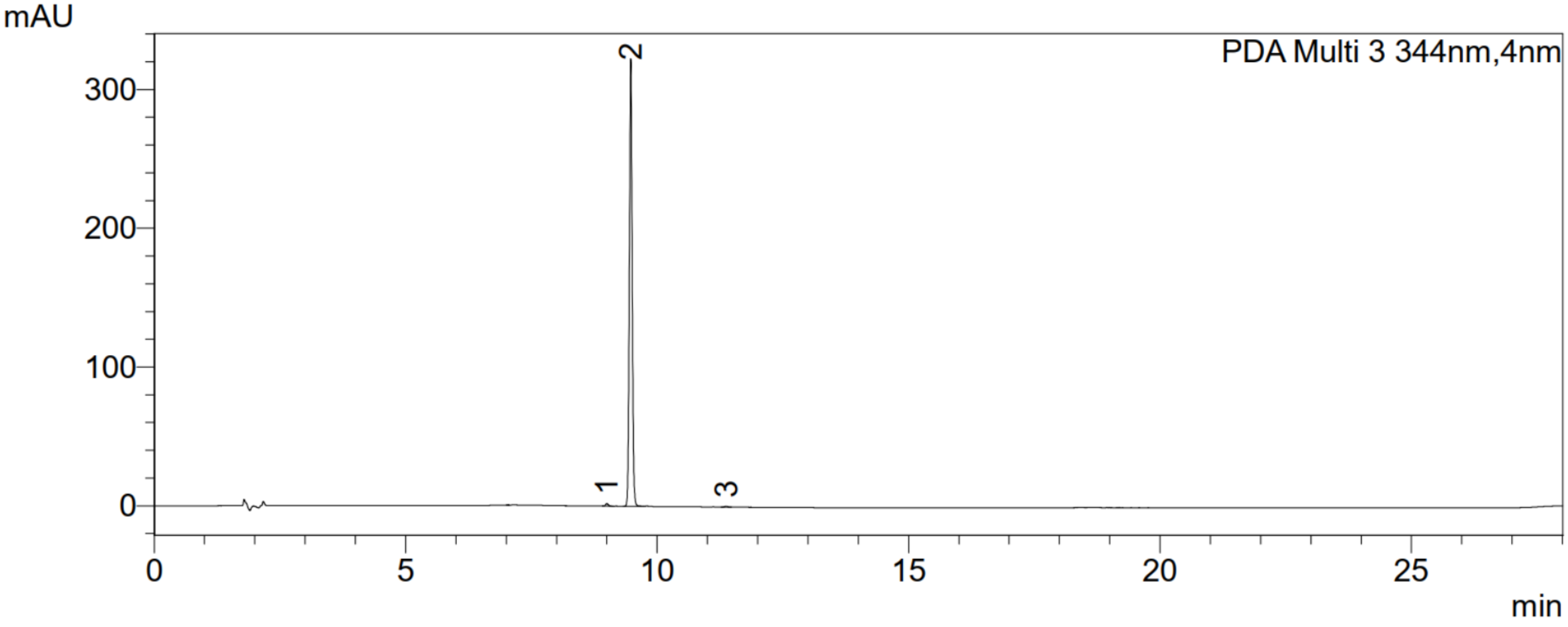


PDA Ch3 344nm

| Peak# | Ret. Time | Name | Area | Area% | Theoretical Plates/meter(USP) | Tailing Factor | Resolution(USP) | Capacity Factor(k') |
|---|---|---|---|---|---|---|---|---|
| 1 | 9,00 | | 6976 | 0,6 | 855954 | 1,180 | -- | -- |
| 2 | 9,48 | | 1250053 | 99,2 | 891655 | 1,038 | 4,672 | 0,053 |
| 3 | 11,37 | | 2502 | 0,2 | 1219149 | 1,039 | 18,030 | 0,263 |
| Total | | | 1259530 | 100,0 | | | | |

**Figure S23.** HPLC-UV of **2e**

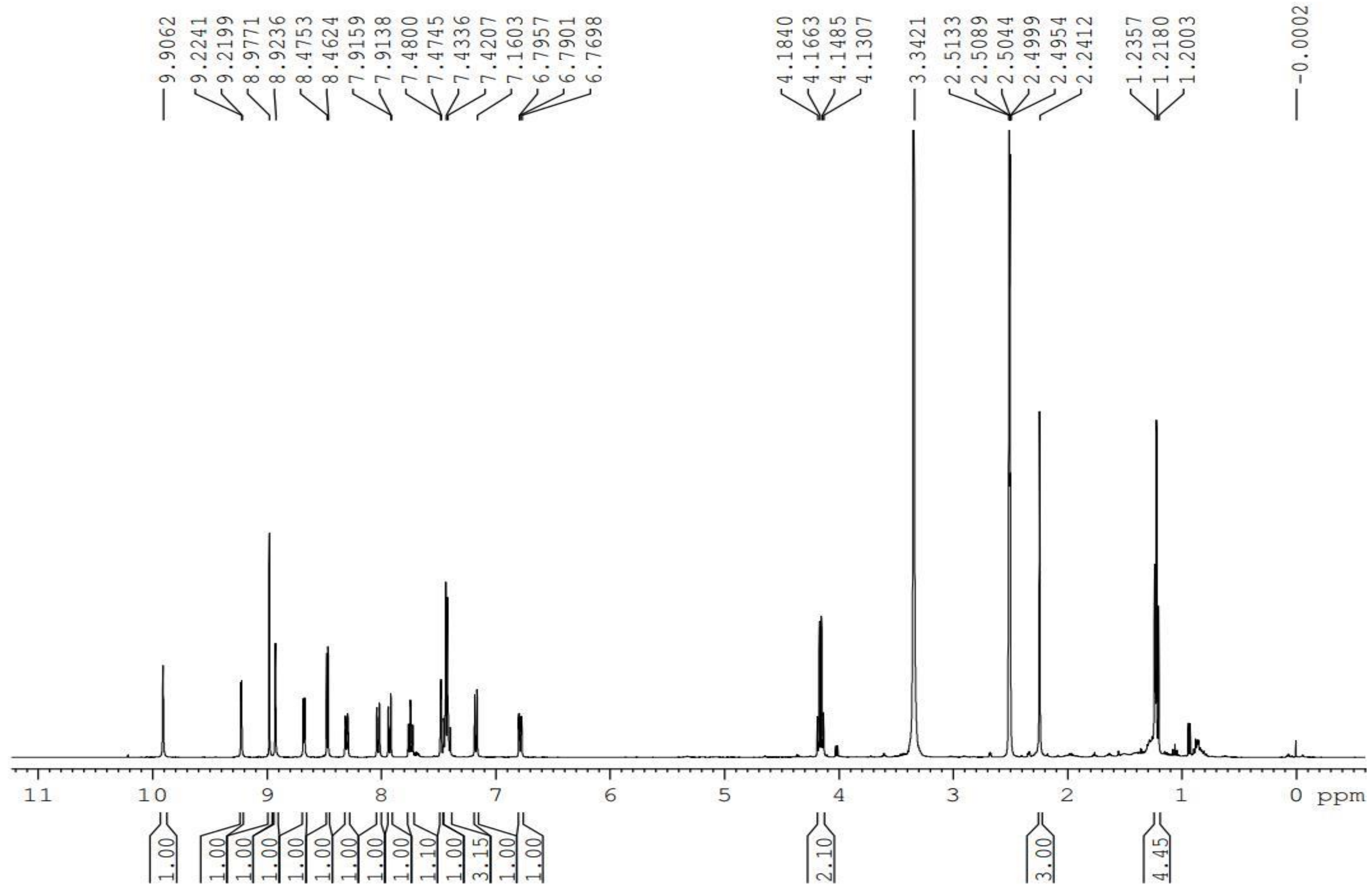


**Figure S24.** $^{1}H$ NMR of **2e**

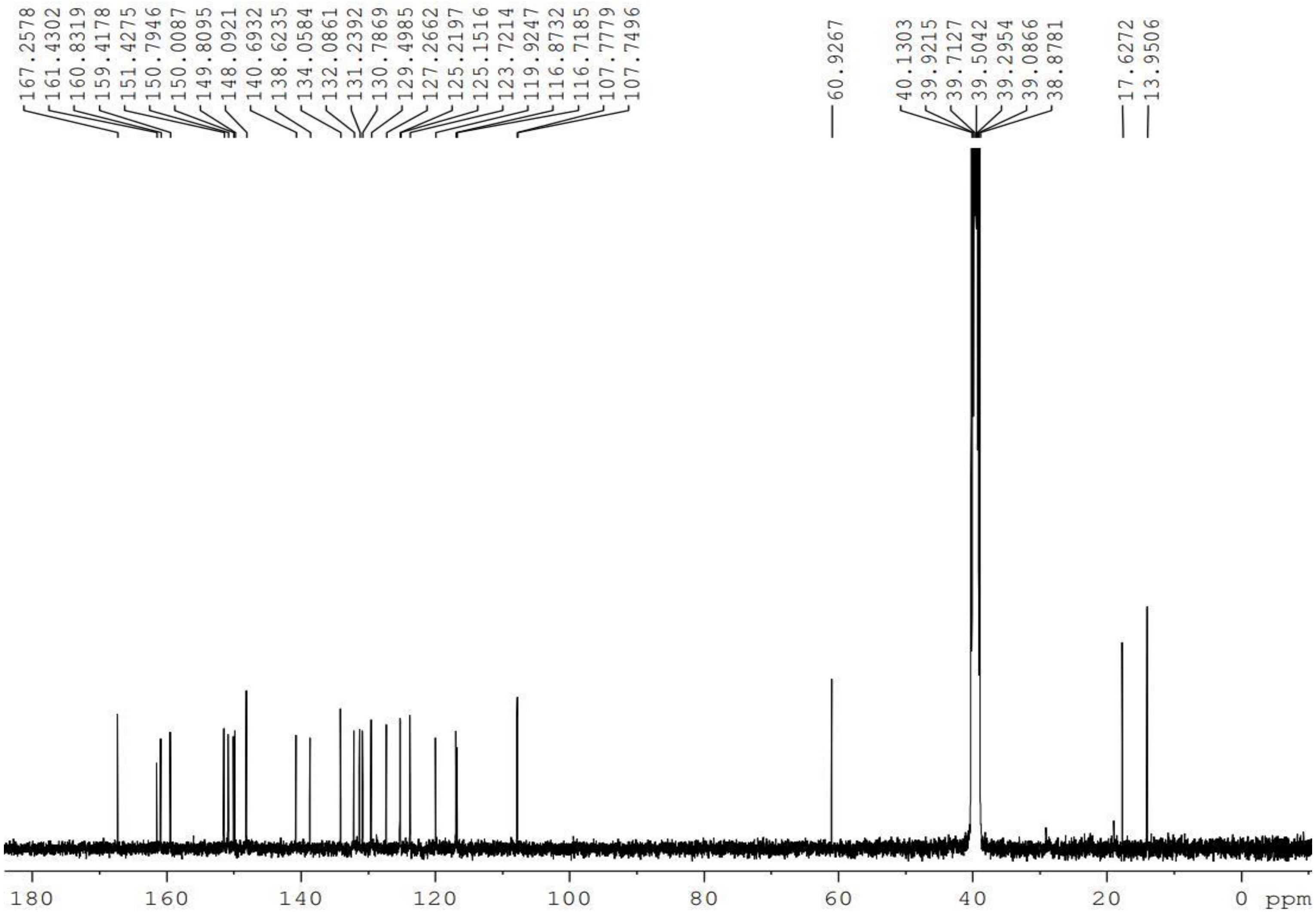


**Figure S25.** $^{13}C$ NMR of **2e**

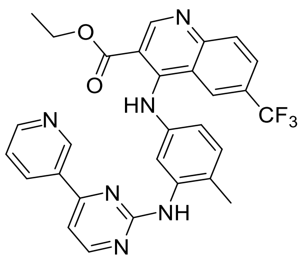

**2f**

ethyl 4-((4-methyl-3-((4-(pyridin-3-yl)pyrimidin-2-yl)amino)phenyl)amino)-6-(trifluoromethyl)quinoline-3-carboxylate

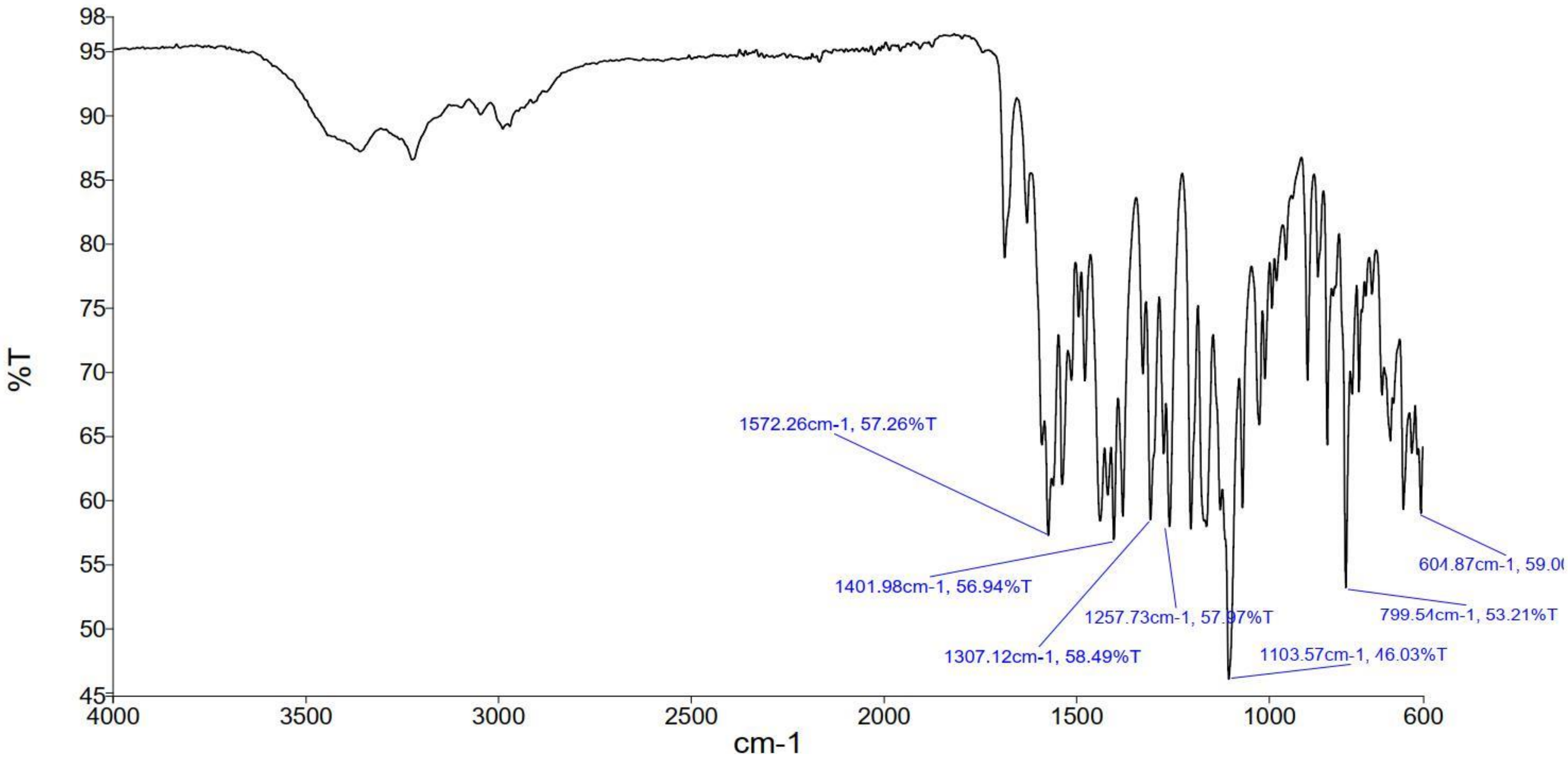


**Figure S26.** IR of **2f**

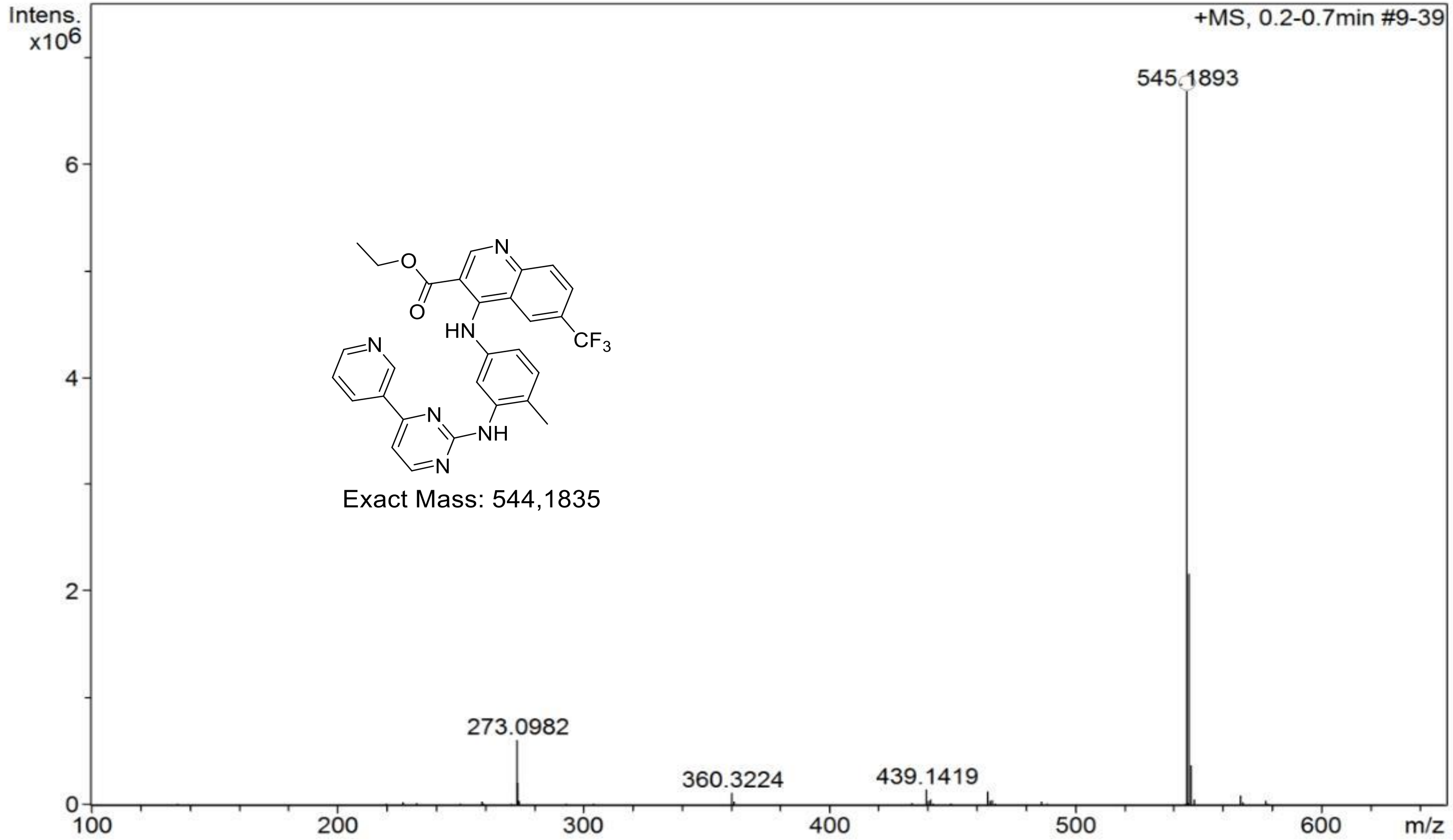


| Meas. m/z | # | Ion Formula | m/z | err [ppm] | Mean err [ppm] | rdb | N-Rule | e⁻ Conf | mSigma | Std I | Std Mean m/z | Std I VarNorm | Std m/z Diff | Std Comb Dev |
|---|---|---|---|---|---|---|---|---|---|---|---|---|---|---|
| 545.189250 | 1 | C29H24F3N6O2 | 545.190735 | 2.7 | 2.9 | 19.5 | ok | even | 7.7 | 12.7 | n.a. | n.a. | n.a. | n.a. |

**Figure S27.** HRMS of **2f**

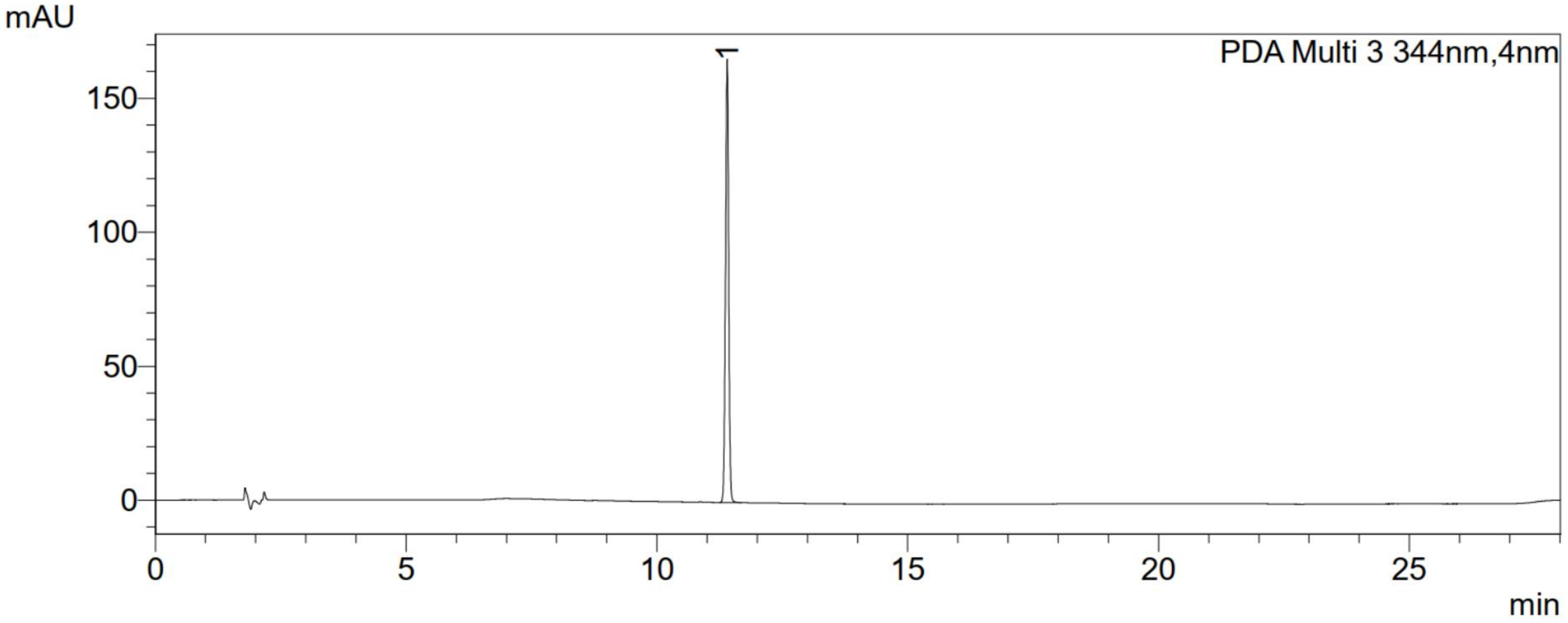


PDA Ch3 344nm

| Peak# | Ret. Time | Name | Area | Area% | Theoretical Plates/meter(USP) | Tailing Factor | Resolution(USP) | Capacity Factor(k') |
|---|---|---|---|---|---|---|---|---|
| 1 | 11,40 | | 721170 | 100,0 | 1012661 | 0,981 | -- | -- |
| Total | | | 721170 | 100,0 | | | | |

**Figure S28.** HPLC-UV of **2f**

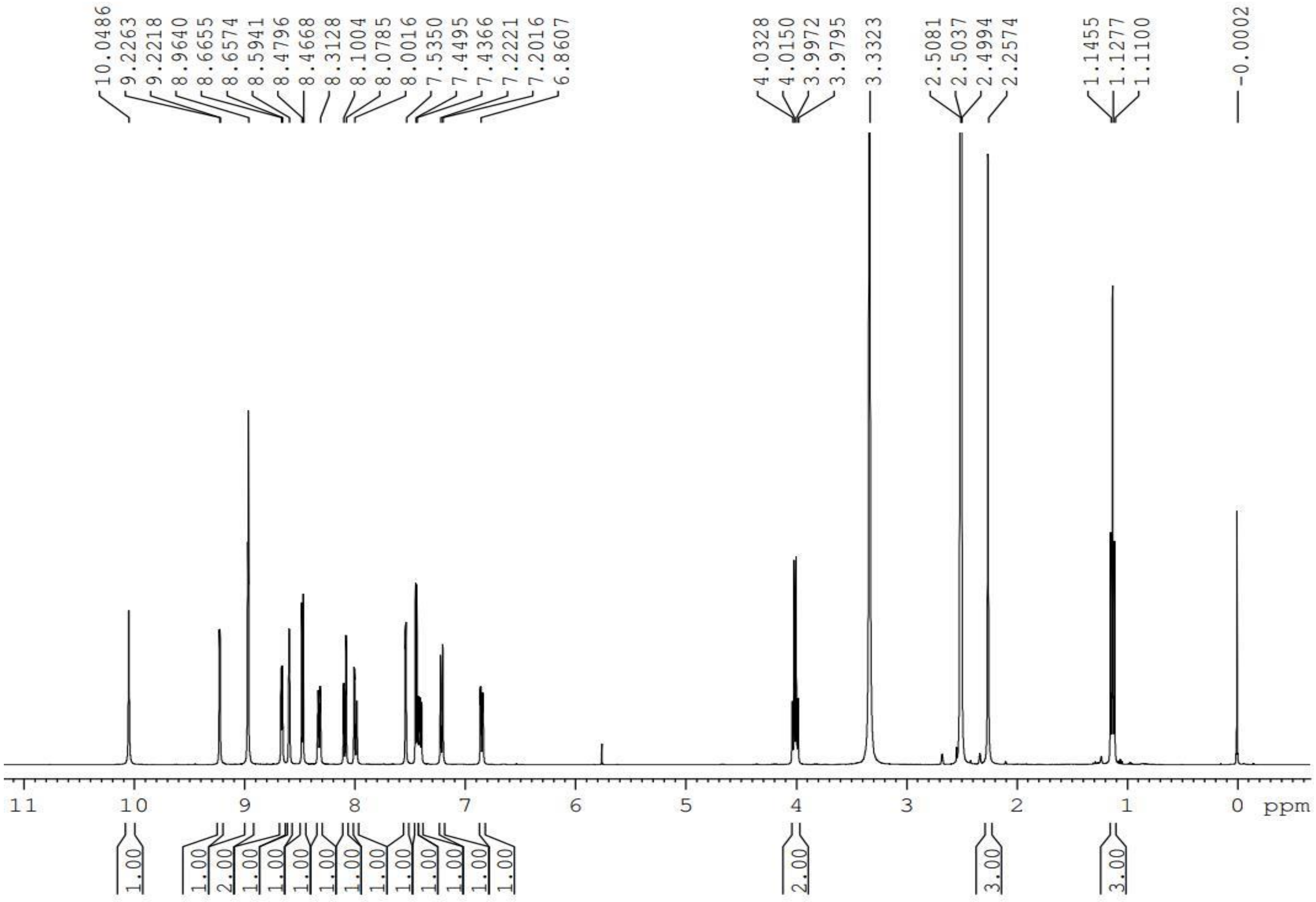

**Figure S29.** $^{1}$H NMR of **2f**

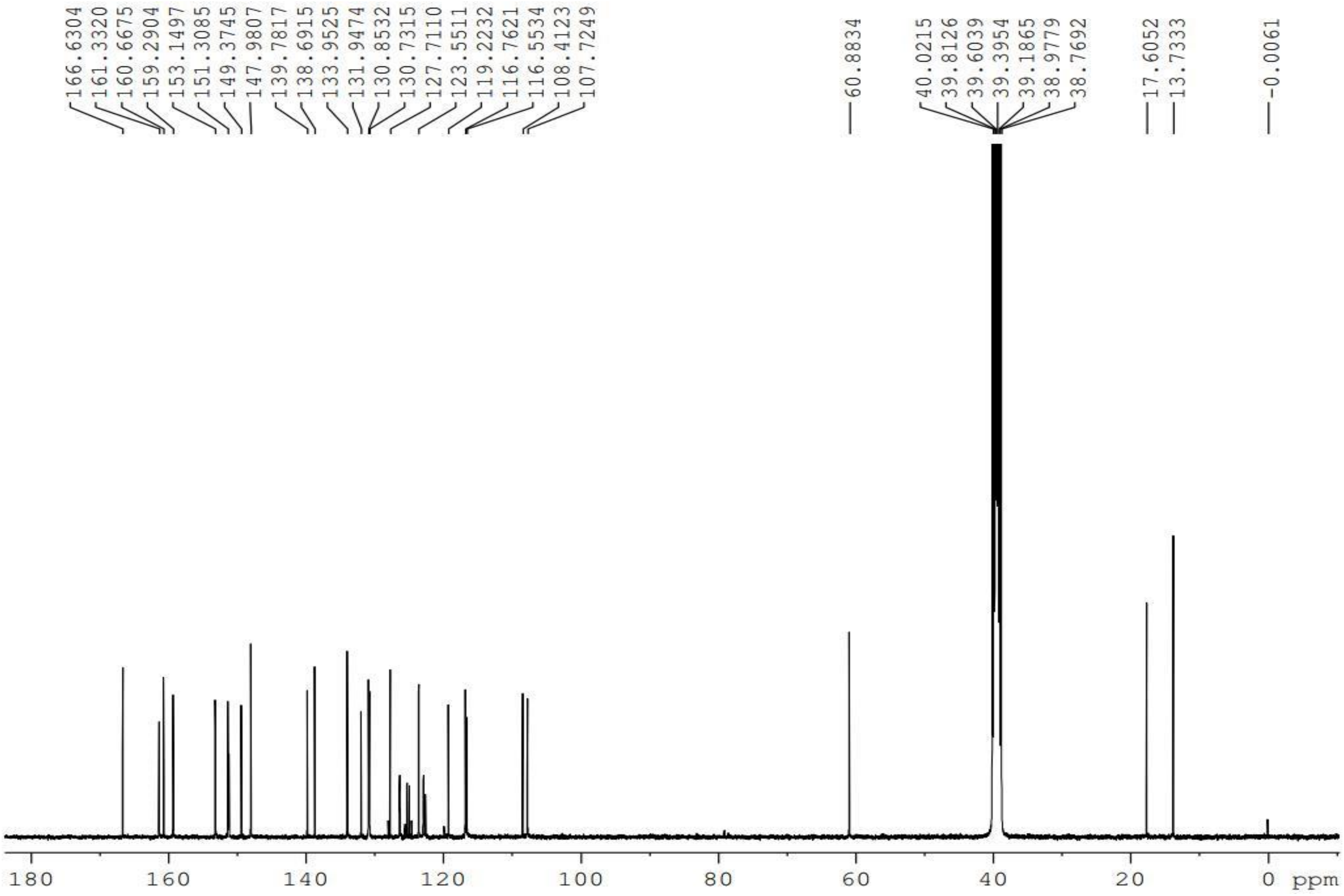


**Figure S30.** $^{13}$C NMR of **2f**

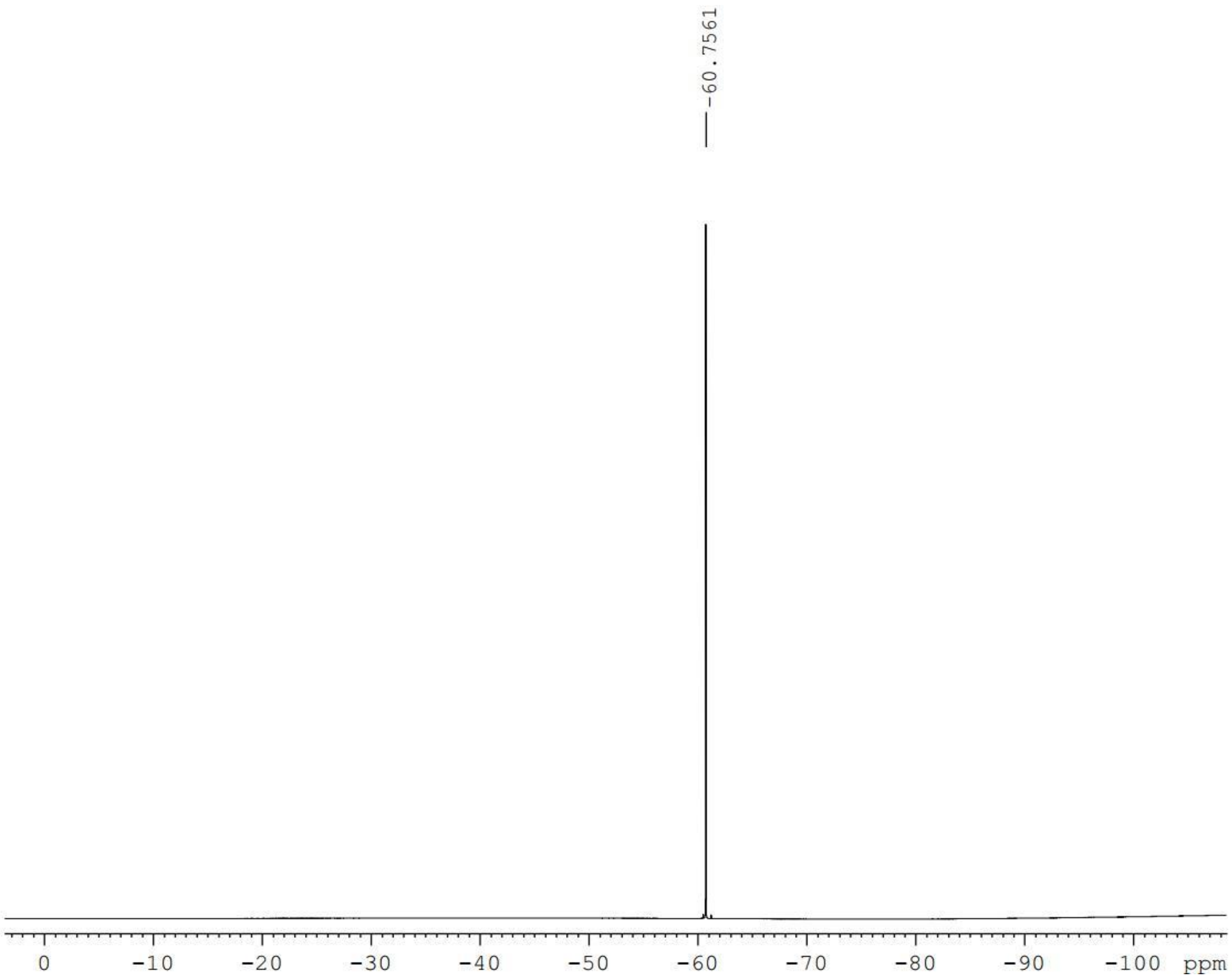


**Figure S31.** $^{19}$F NMR of **2f**

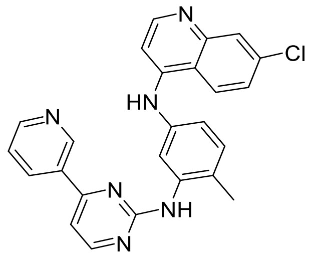

**2g**

*N*[1]-(7-chloroquinolin-4-yl)-4-methyl-*N*[3]-(4-(pyridin-3-yl)pyrimidin-2-yl)benzene-1,3-diamine

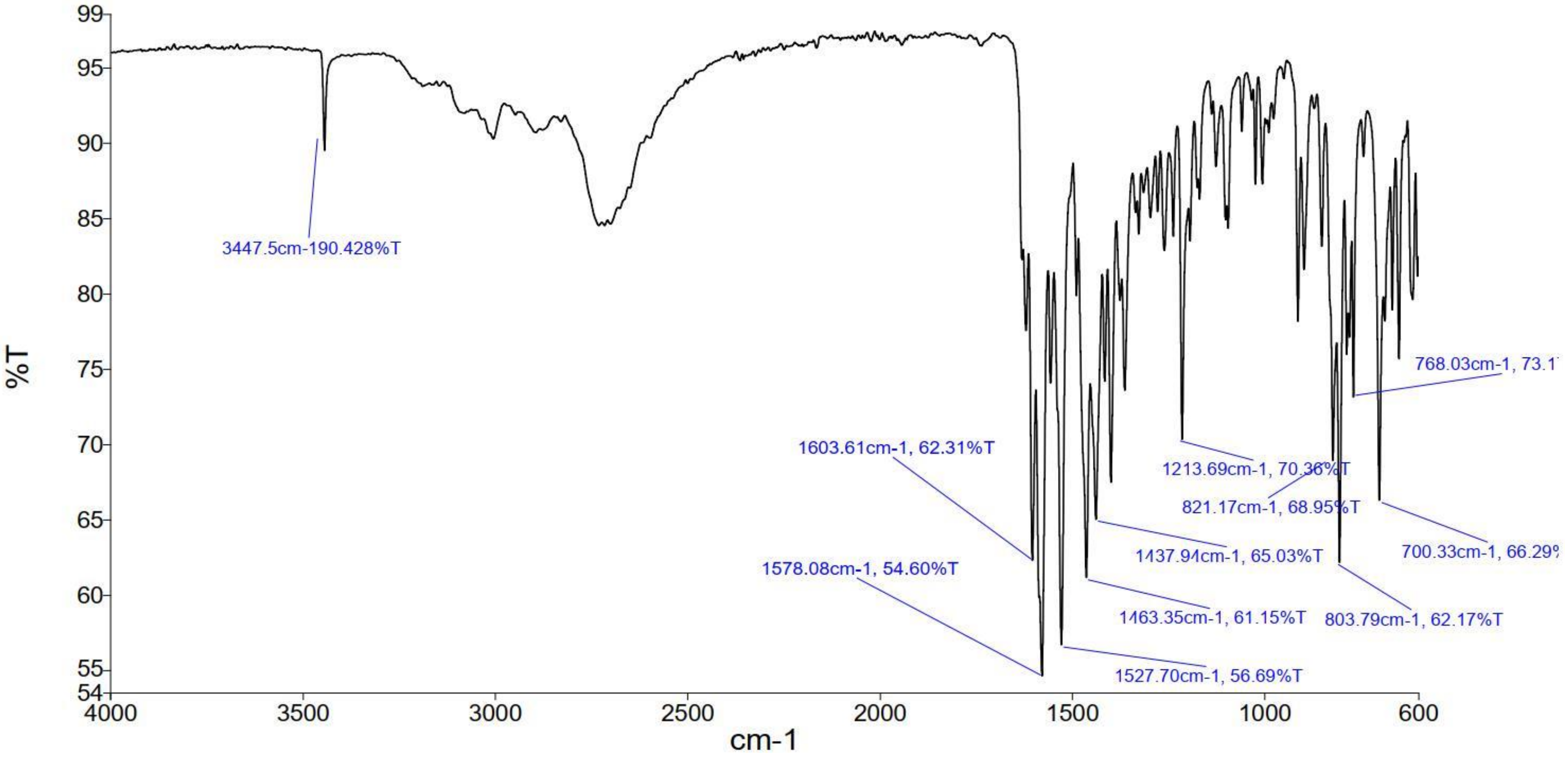


**Figure S32.** IR of **2g**

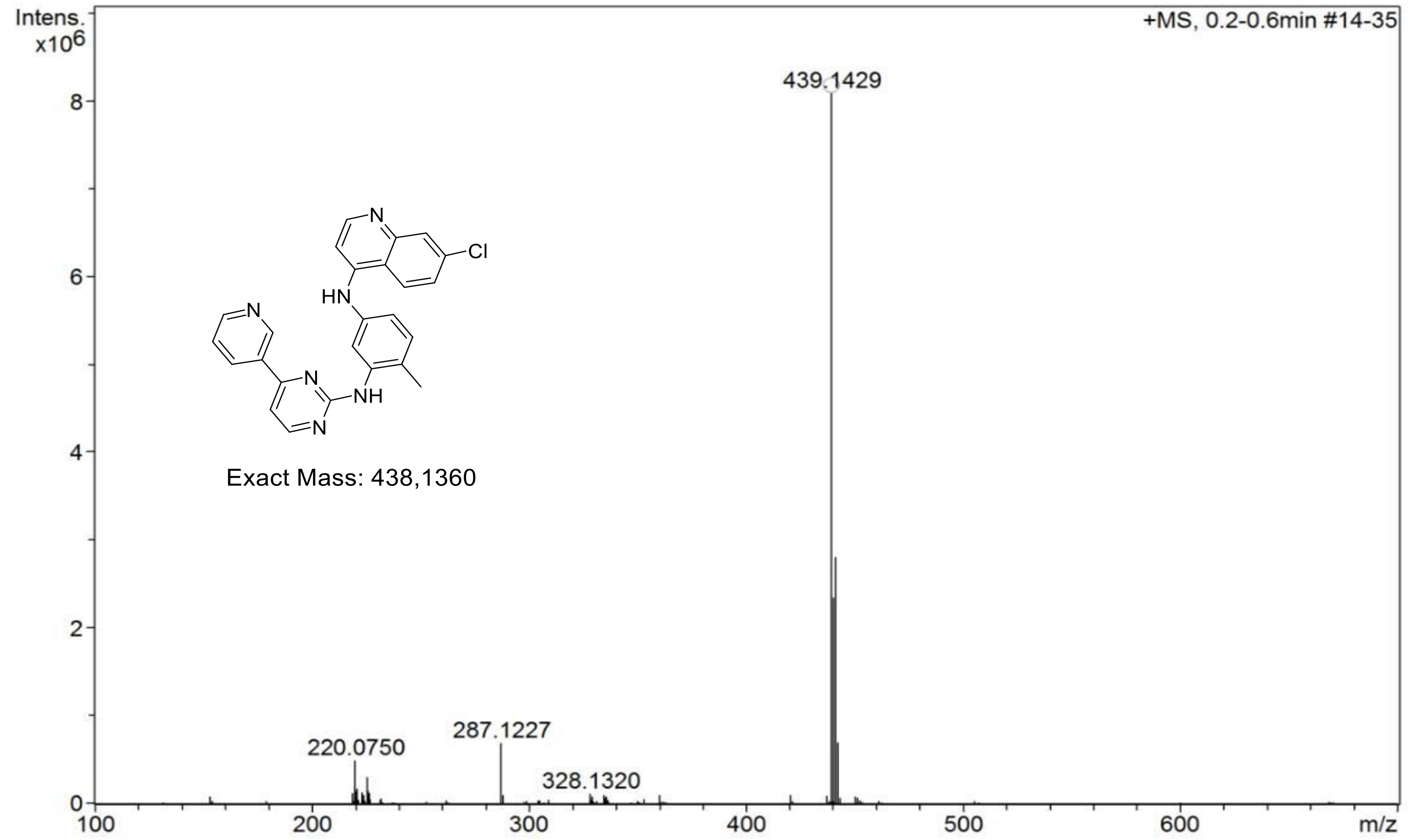


| Meas. m/z | # | Ion Formula | m/z | err [ppm] | Mean err [ppm] | rdb | N-Rule | $e^-$ Conf | mSigma | Std I | Std Mean m/z | Std I VarNorm | Std m/z Diff | Std Comb Dev |
|---|---|---|---|---|---|---|---|---|---|---|---|---|---|---|
| 439.142908 | 1 | $C_{25}H_{20}ClN_6$ | 439.143249 | 0.8 | 1.2 | 18.5 | ok | even | 8.6 | 13.7 | n.a. | n.a. | n.a. | n.a. |
| | 2 | $C_{20}H_{20}ClN_8O_2$ | 439.139226 | -8.4 | -8.5 | 14.5 | ok | even | 19.1 | 25.8 | n.a. | n.a. | n.a. | n.a. |

**Figure S33.** HRMS of **2g**

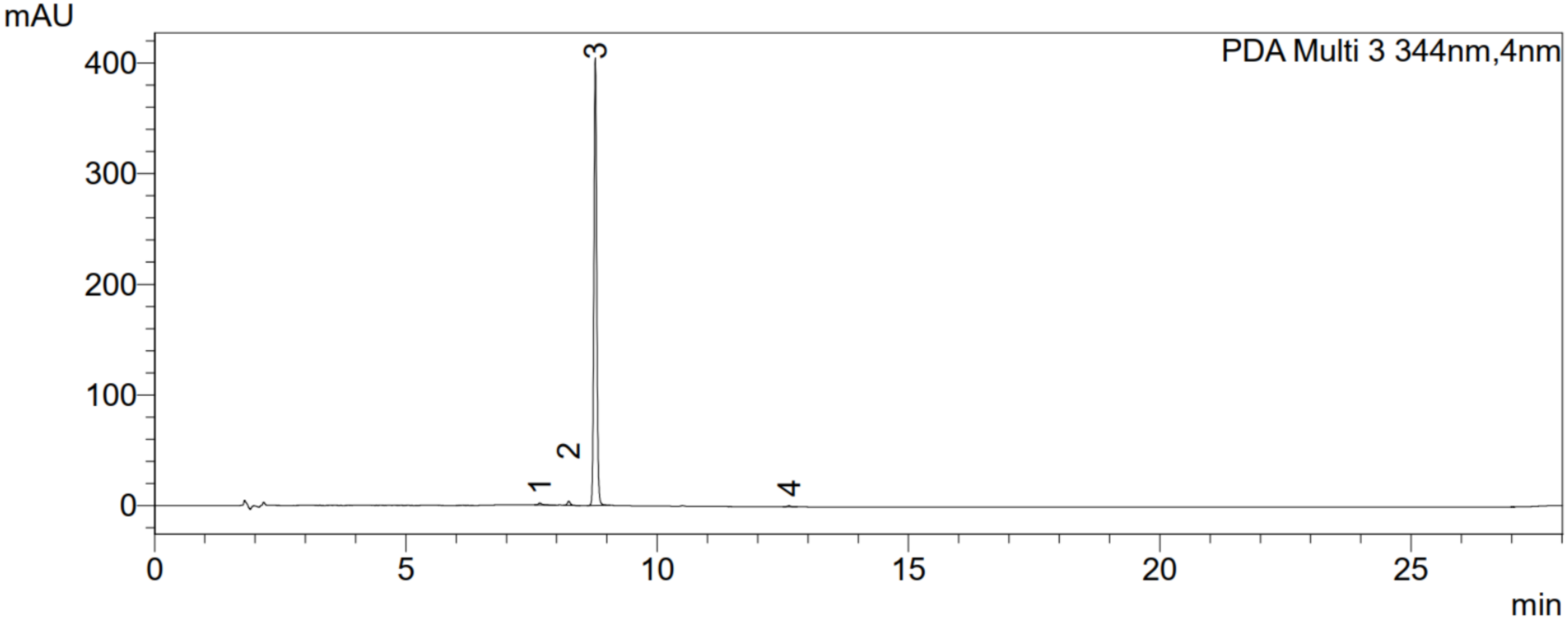


PDA Ch3 344nm

| Peak# | Ret. Time | Name | Area | Area% | Theoretical Plates/meter(USP) | Tailing Factor | Resolution(USP) | Capacity Factor(k') |
|---|---|---|---|---|---|---|---|---|
| 1 | 7,66 | | 10177 | 0,6 | 423830 | 1,795 | -- | -- |
| 2 | 8,24 | | 15299 | 1,0 | 661130 | 1,222 | 5,102 | 0,075 |
| 3 | 8,77 | | 1564097 | 98,1 | 773384 | 1,055 | 5,106 | 0,144 |
| 4 | 12,62 | | 4578 | 0,3 | 1103482 | 0,990 | 33,925 | 0,647 |
| Total | | | 1594151 | 100,0 | | | | |

**Figure S34.** HPLC-UV of **2g**

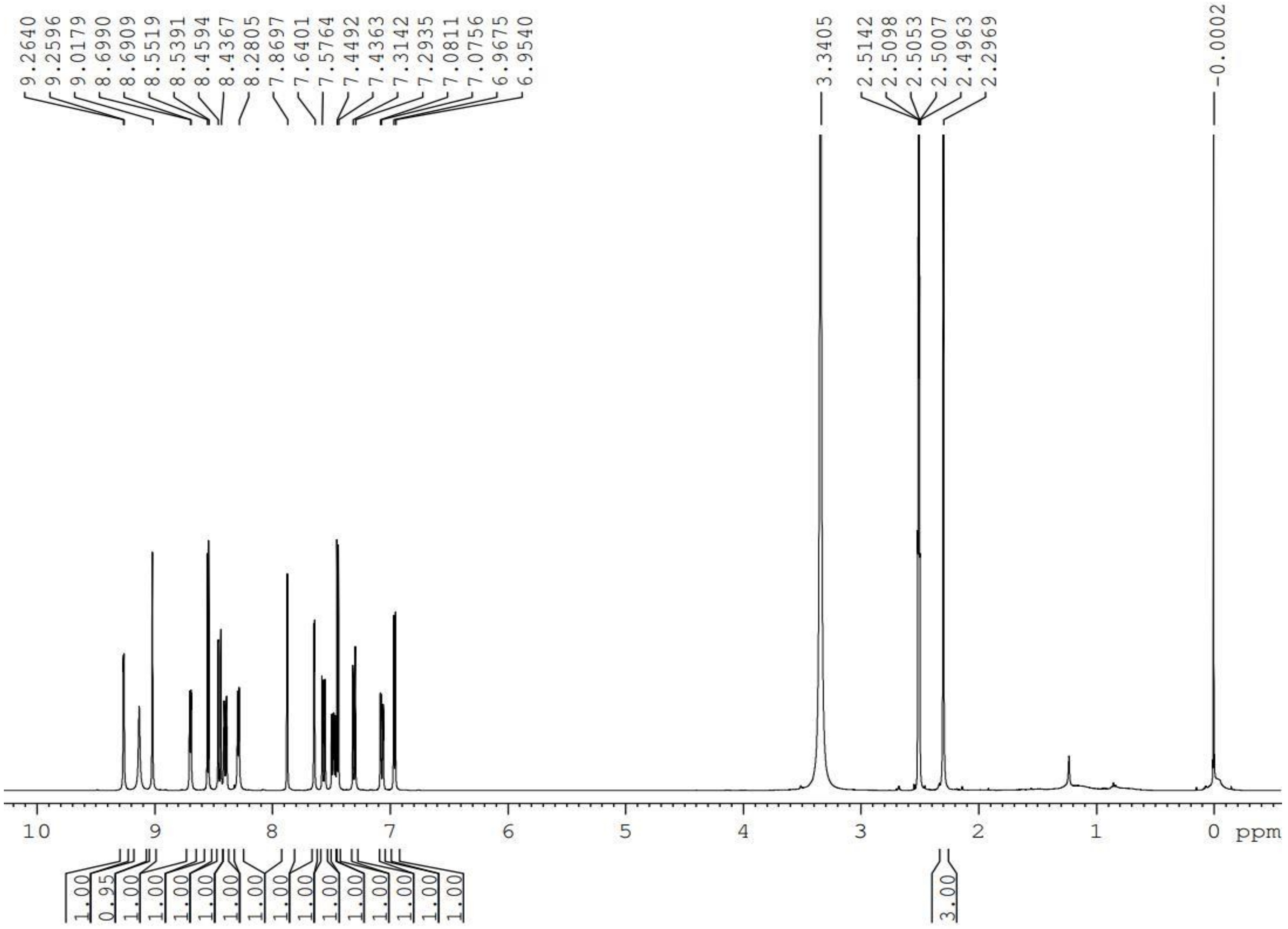


**Figure S35.** $^{1}H$ NMR of **2g**

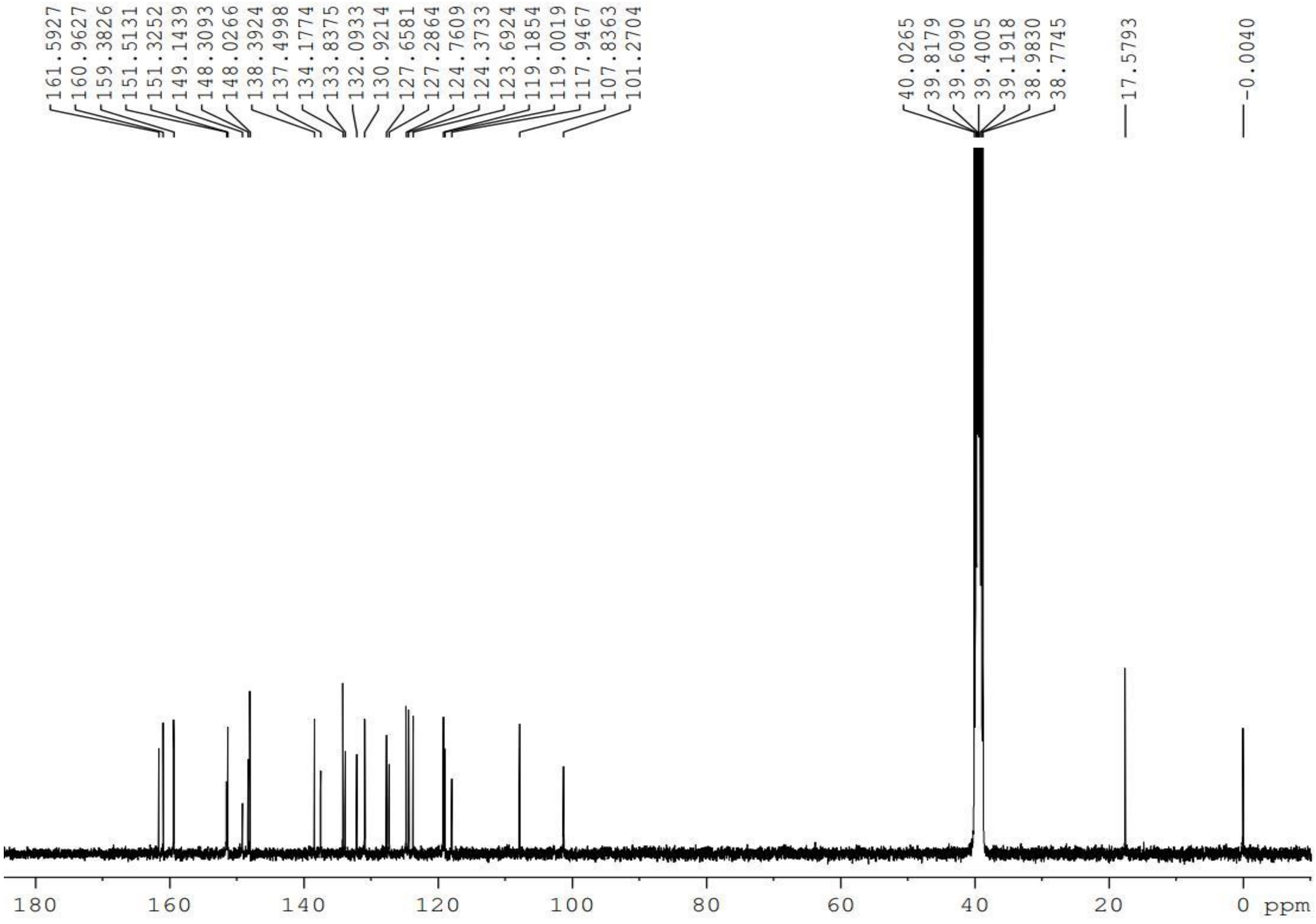


**Figure S36.** $^{13}$C NMR of **2g**

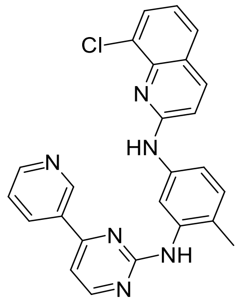


**2h**

*N*[1]-(8-chloroquinolin-2-yl)-4-methyl-*N*[3]-(4-(pyridin-3-yl)pyrimidin-2-yl)benzene-1,3-diamine

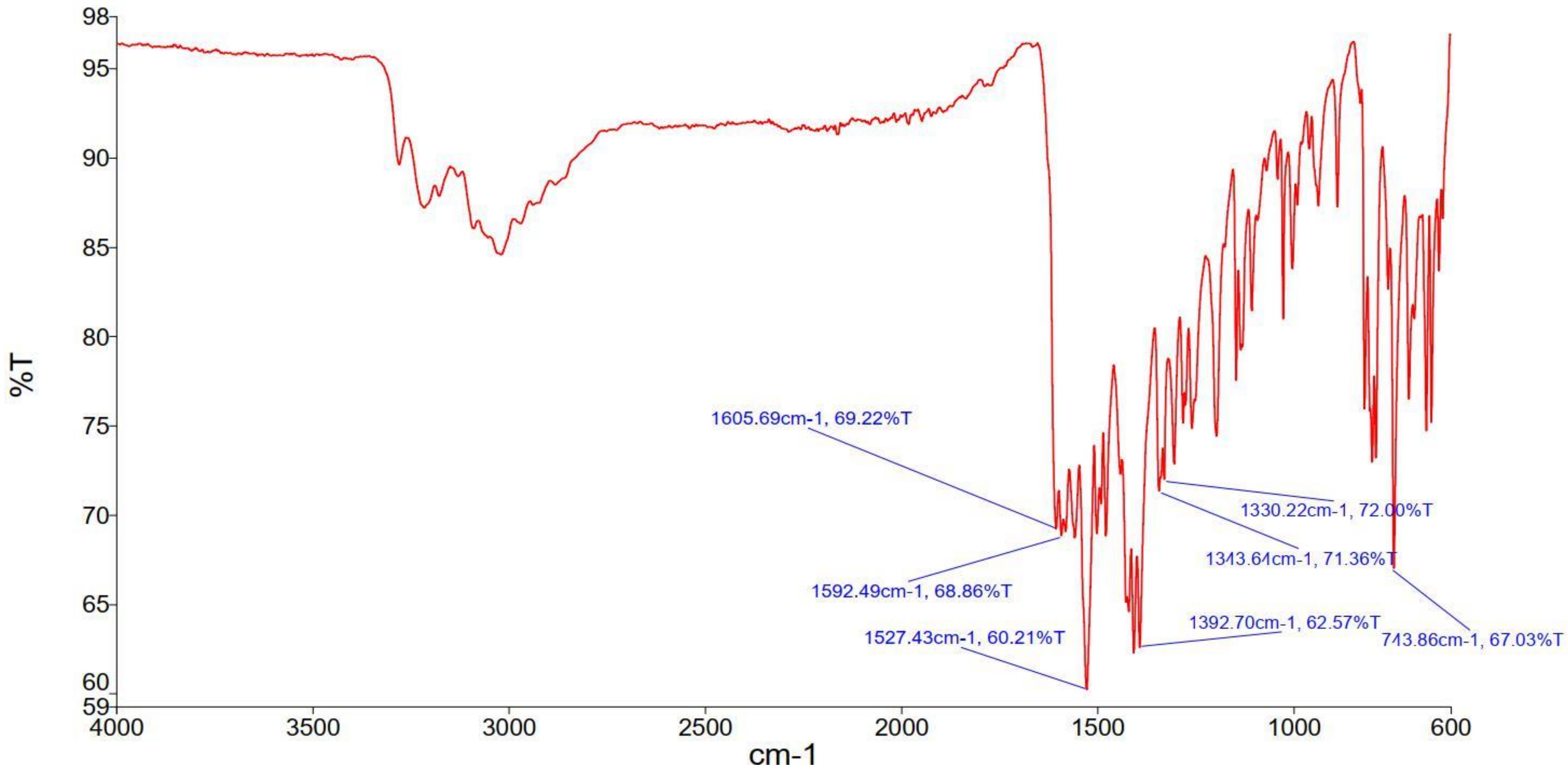


**Figure S37.** IR of **2h**

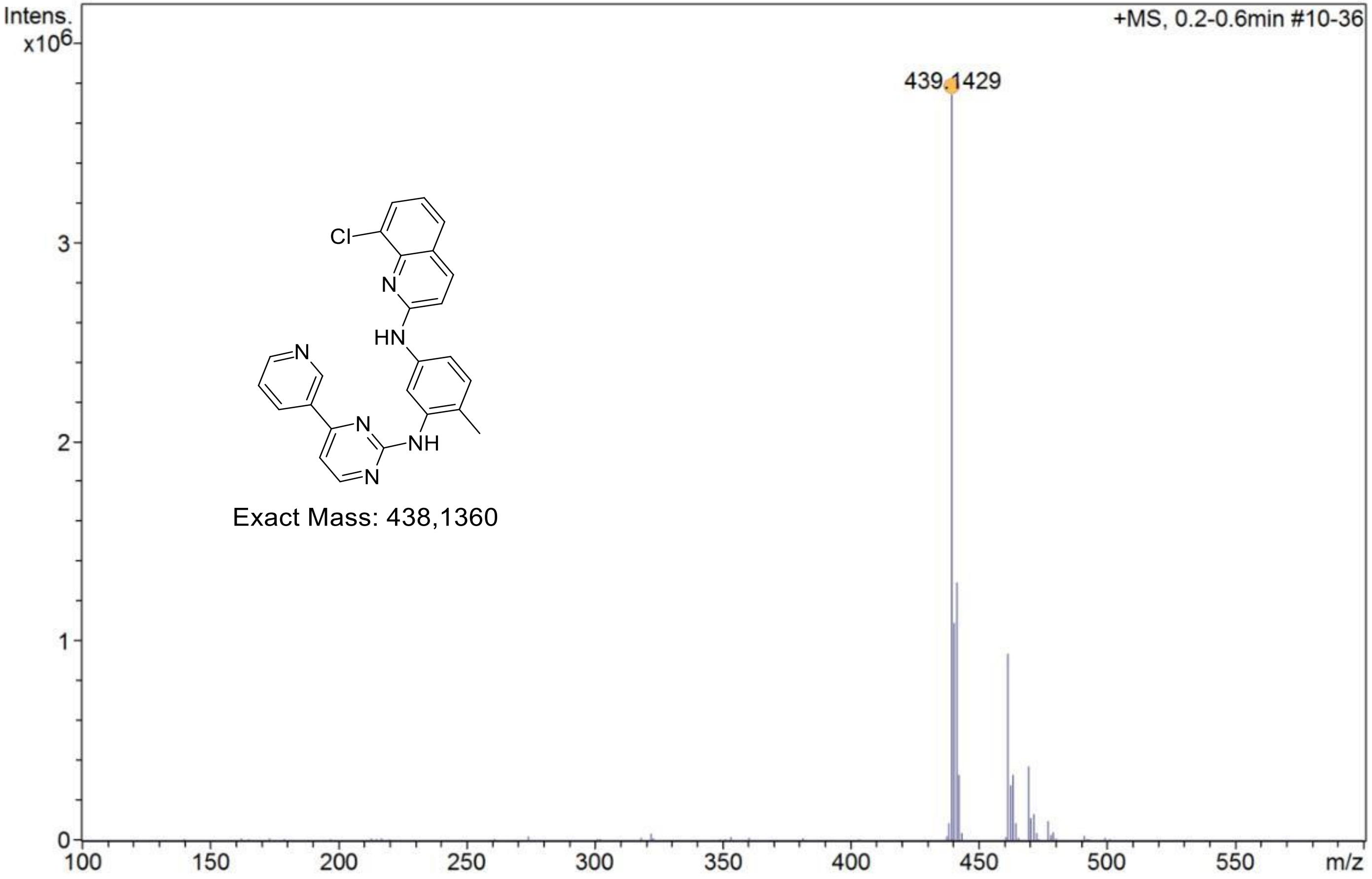


| Meas. m/z | # | Ion Formula | m/z | err [ppm] | Mean err [ppm] | rdb | N-Rule | e⁻ Conf | mSigma | Std I | Std Mean m/z | Std I VarNorm | Std m/z Diff | Std Comb Dev |
|---|---|---|---|---|---|---|---|---|---|---|---|---|---|---|
| 439.142923 | 1 | C25H20ClN6 | 439.143249 | 0.7 | 1.1 | 18.5 | ok | even | 8.7 | 12.9 | n.a. | n.a. | n.a. | n.a. |
| | 2 | C24H24ClN2O4 | 439.141911 | -2.3 | -1.5 | 13.5 | ok | even | 12.5 | 16.2 | n.a. | n.a. | n.a. | n.a. |

**Figure S38.** HRMS of **2h**

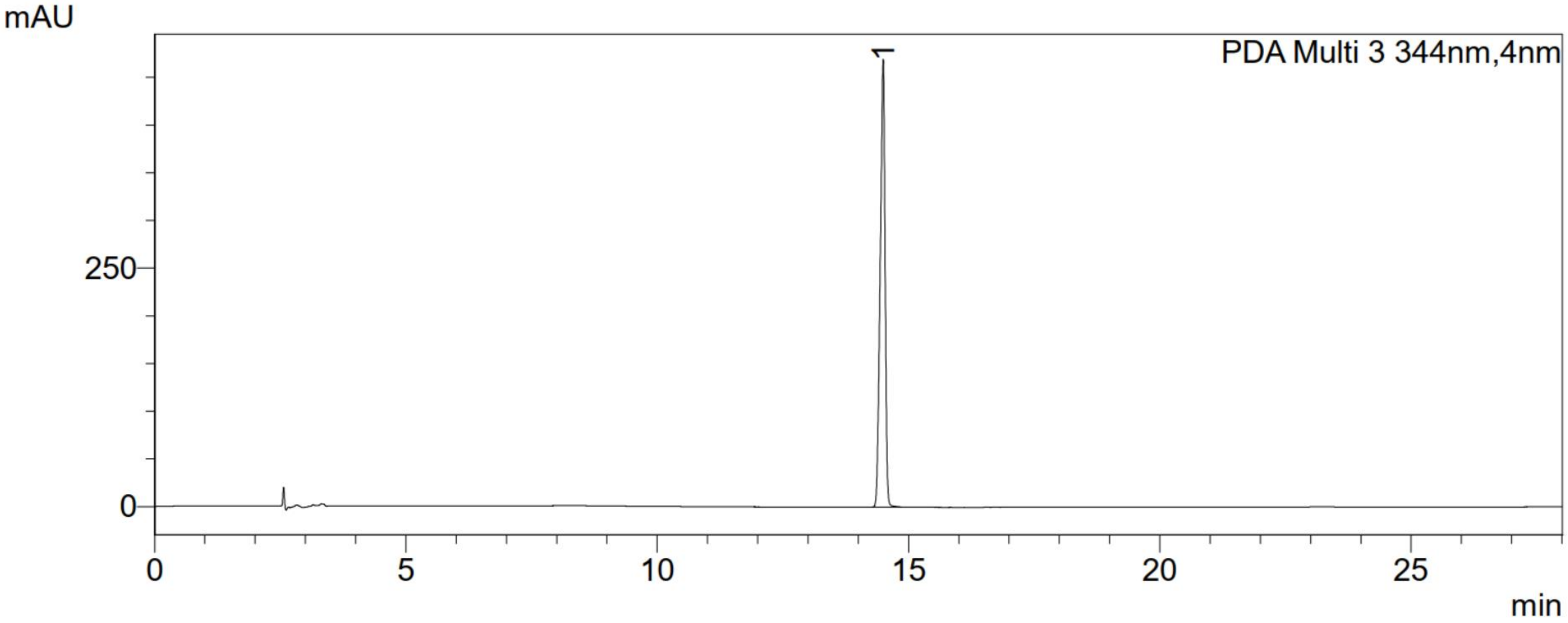


PDA Ch3 344nm

| Peak# | Ret. Time | Name | Area | Area% | Theoretical Plates/meter(USP) | Tailing Factor | Resolution(USP) | Capacity Factor(k') |
|---|---|---|---|---|---|---|---|---|
| 1 | 14,49 | | 3172518 | 100,0 | 610456 | 0,852 | -- | -- |
| Total | | | 3172518 | 100,0 | | | | |

**Figure S39.** HPLC-UV of **2h**

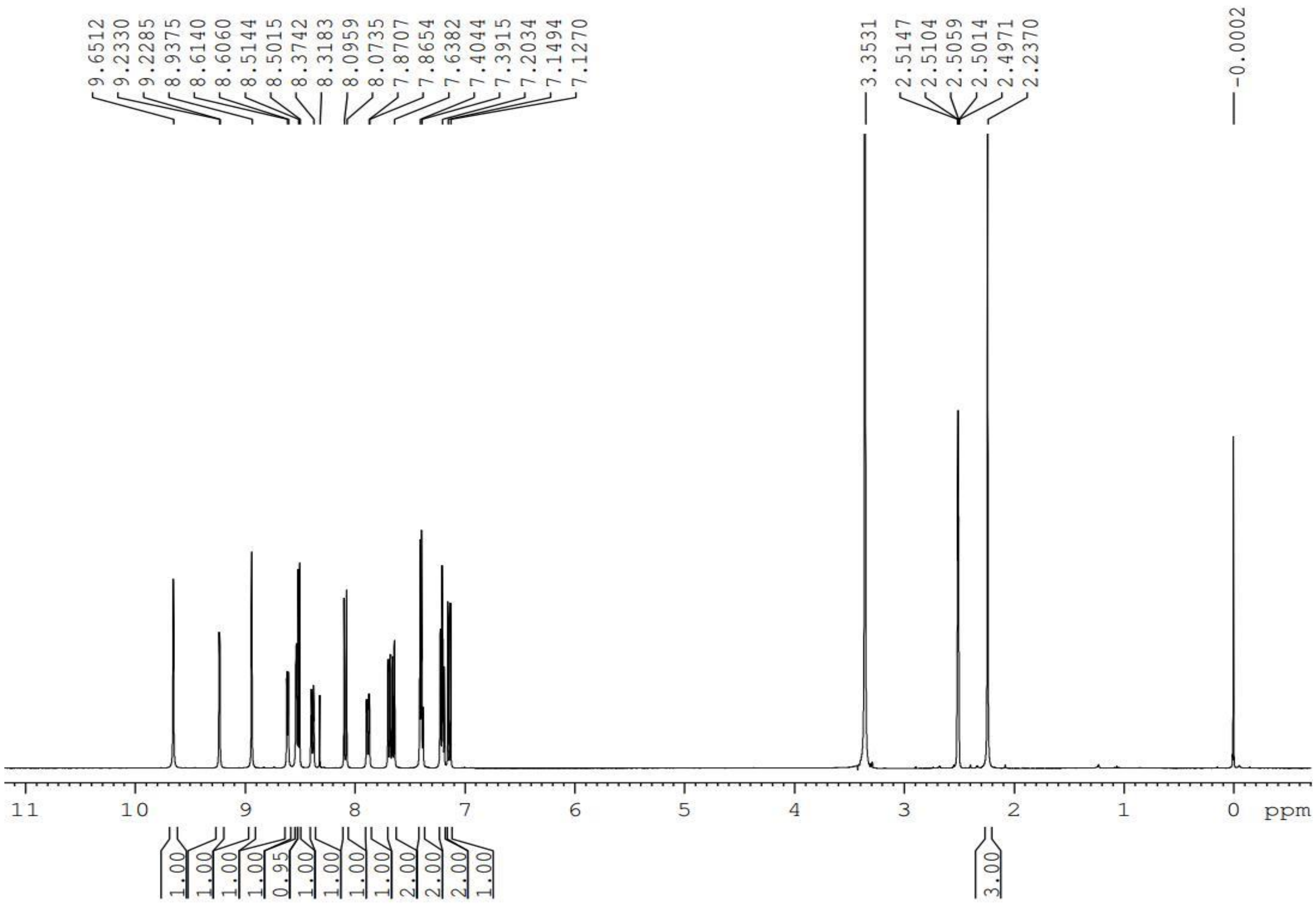


**Figure S40.** $^{1}$H NMR of **2h**

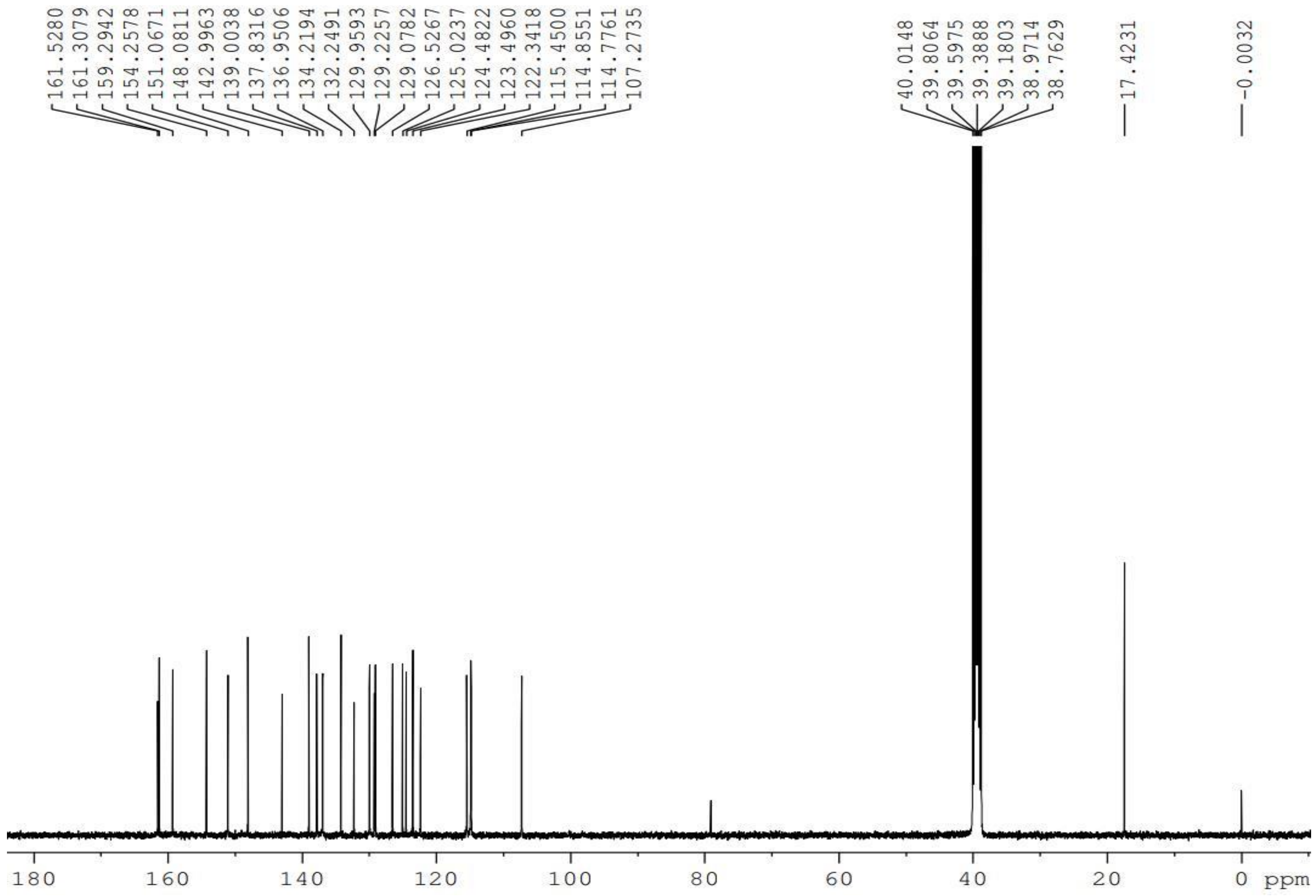


**Figure S41.** $^{13}C$ NMR of **2h**

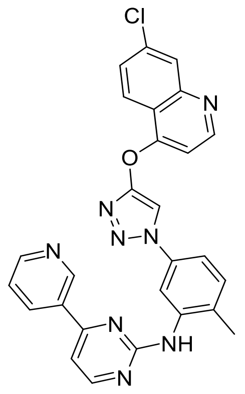

**3a**

*N*-(5-(4-((7-chloroquinolin-4-yl)oxy)-1H-1,2,3-triazol-1-yl)-2-methylphenyl)-4-(pyridin-3-yl)pyrimidin-2-amine

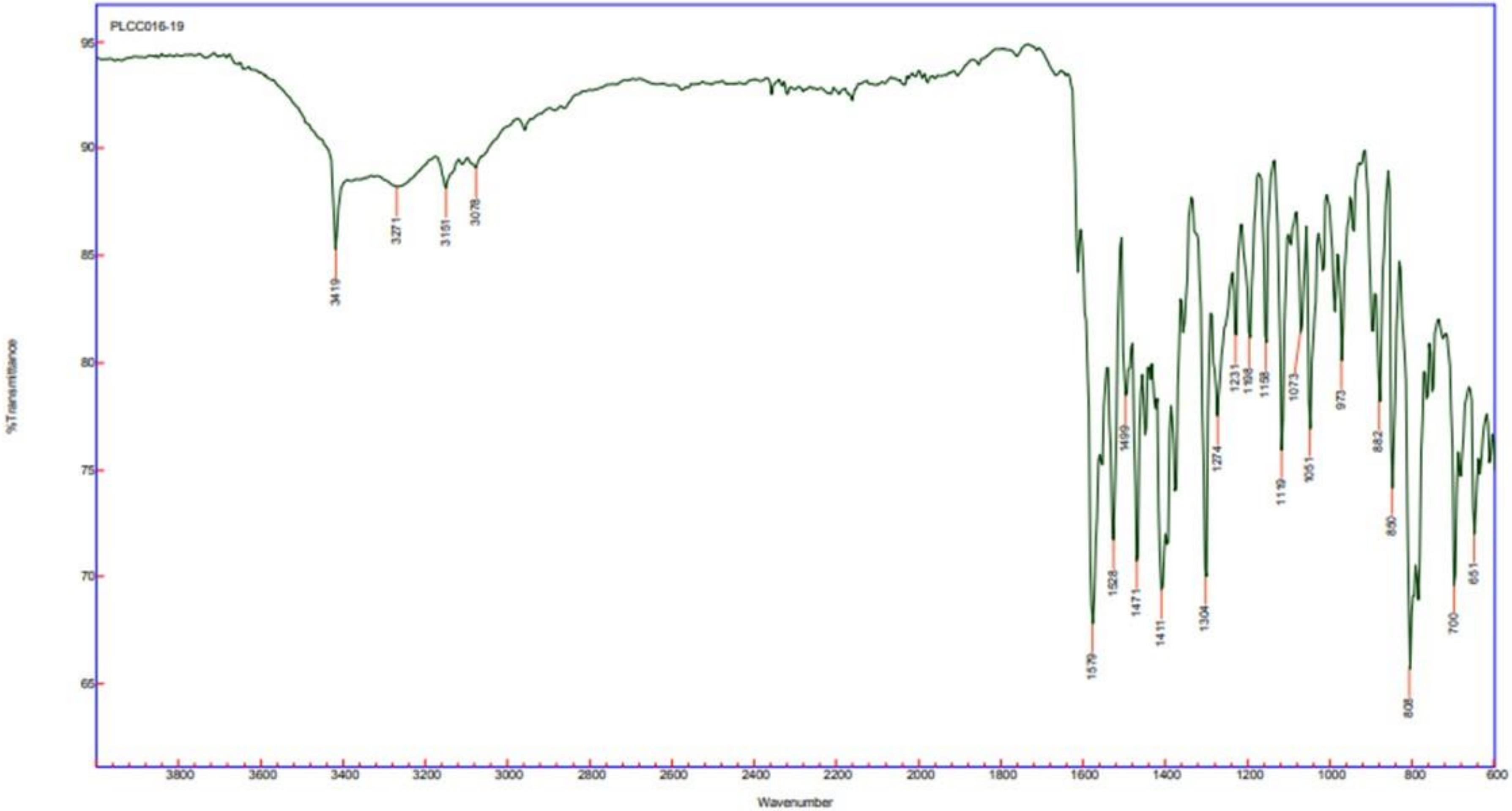


**Figure S42.** IR of **3a**

| | | | |
|---|---|---|---|
| **Analysis Info** | | Acquisition Date | 3/20/2019 8:43:49 AM |
| Analysis Name | D:\Data\2019\sintese 1\INFUSAO\132612 - PLCC 016-19.d | | |
| Method | Tune_pos_Standard.m | Operator | Vinicius |
| Sample Name | 132612 - PLCC 016-19 | Instrument | compact 8255754.10035 |
| Comment | | | |

**Acquisition Parameter**

| | | | | | |
|---|---|---|---|---|---|
| Source Type | ESI | Ion Polarity | Positive | Set Nebulizer | 0.4 Bar |
| Focus | Not active | Set Capillary | 4000 V | Set Dry Heater | 200 °C |
| Scan Begin | 80 m/z | Set End Plate Offset | -400 V | Set Dry Gas | 4.0 l/min |
| Scan End | 1000 m/z | Set Charging Voltage | 2000 V | Set Divert Valve | Source |
| | | Set Corona | 0 nA | Set APCI Heater | 0 °C |

+MS, 0.4-0.7min #24-39

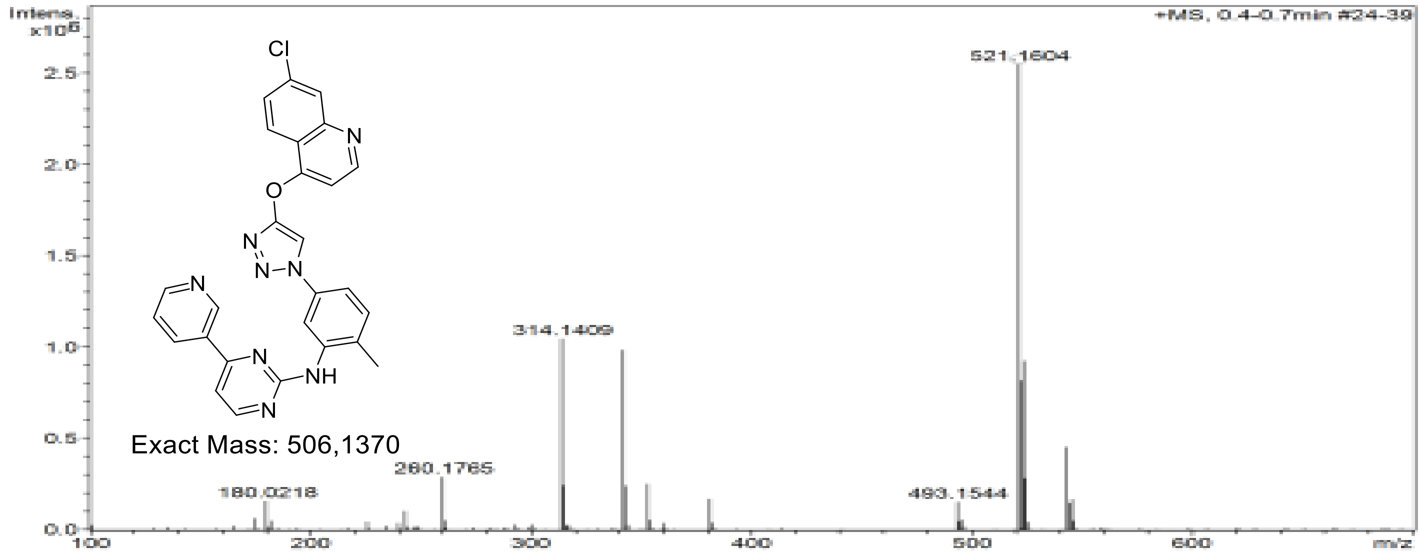


| Meas. m/z | # | Ion Formula | m/z | err [ppm] | Mean err [ppm] | rdb | N-Rule | e⁻ Conf | mSigma | Std I | Std Mean m/z | Std I VarNorm | Std m/z Diff | Std Comb Dev |
|---|---|---|---|---|---|---|---|---|---|---|---|---|---|---|
| 521.160353 | 1 | C28H22ClN8O | 521.159961 | -0.8 | -0.7 | 21.5 | ok | even | 8.4 | 10.7 | n.a. | n.a. | n.a. | n.a. |

**Figure S43.** HRMS of **3a**

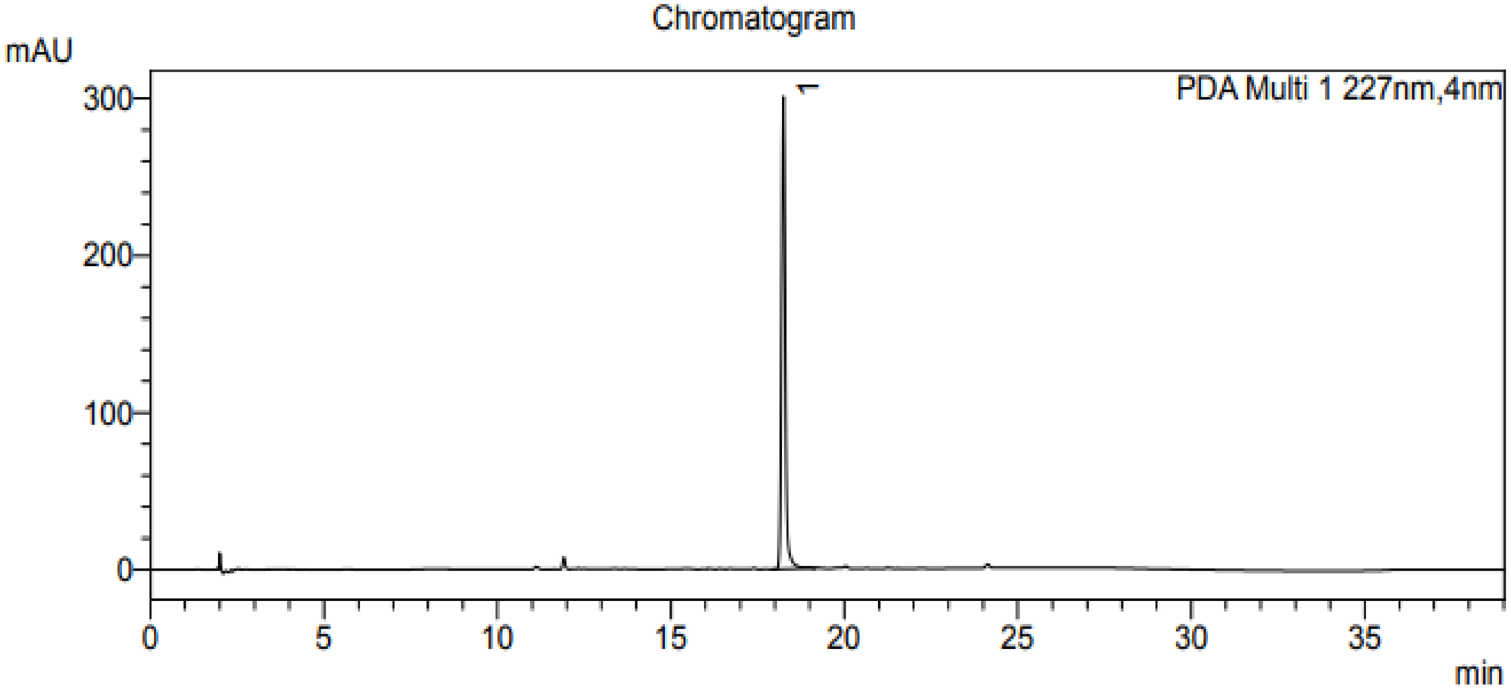


Peak Table

PDA Ch1 227nm

| Peak# | Ret. Time | Name | Area | Area% | Theoretical Plates/meter(USP) | Tailing Factor | Resolution(USP) | Capacity Factor(k') |
|---|---|---|---|---|---|---|---|---|
| 1 | 18,25 | | 2286223 | 100,0 | 825445 | 1,136 | -- | -- |
| Total | | | 2286223 | 100,0 | | | | |

**Figure S44.** HPLC-UV of **3a**

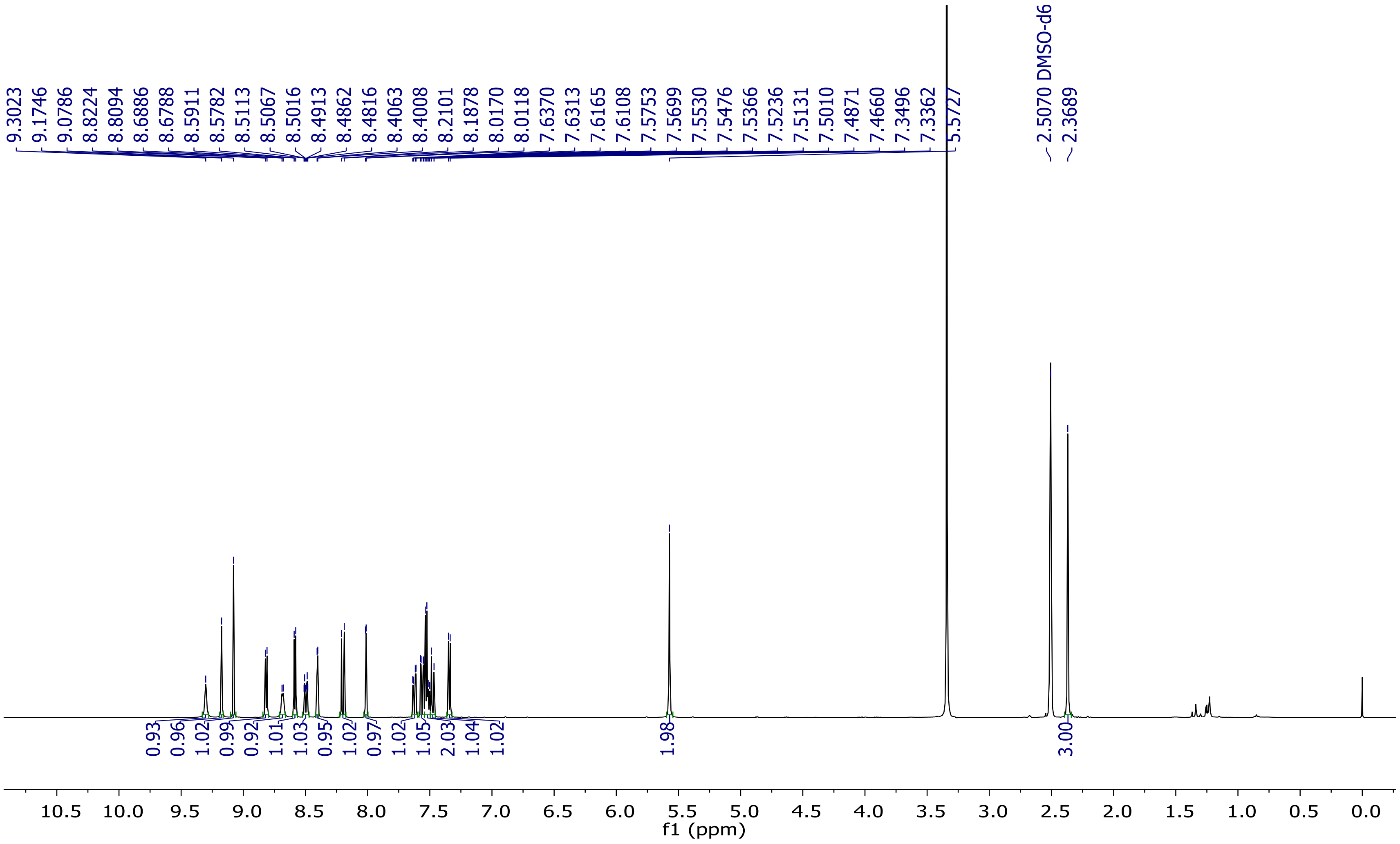


**Figure S45.** $^{1}H$ NMR of **3a**

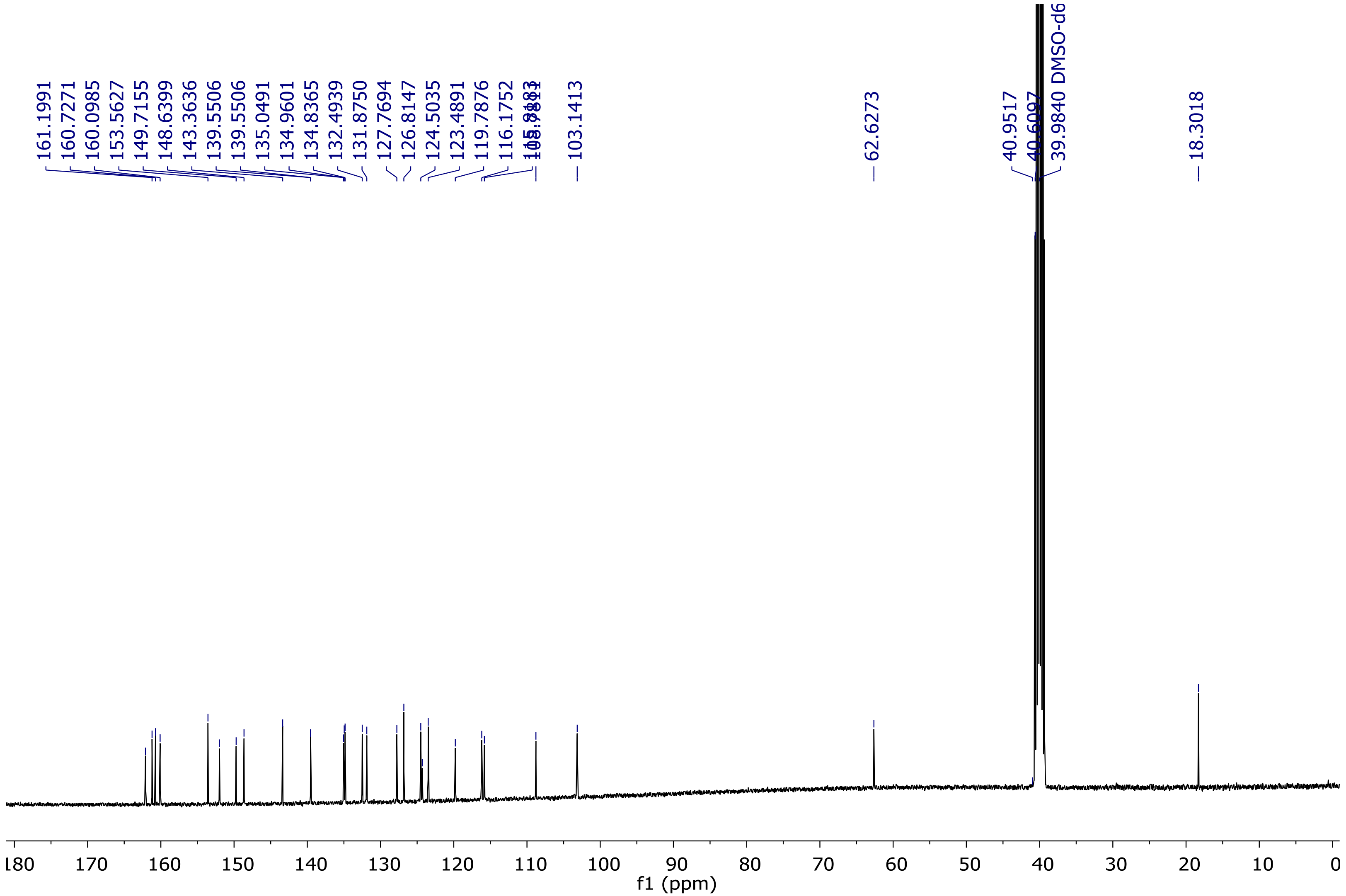


**Figure S46.** $^{13}$C NMR of **3a**

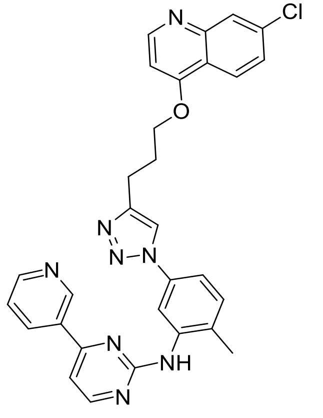


**3b**

*N*-(5-(4-(3-((7-chloroquinolin-4-yl)oxy)propyl)-1*H*-1,2,3-triazol-1-yl)-2-methylphenyl)-4-(pyridin-3-yl)pyrimidin-2-amine

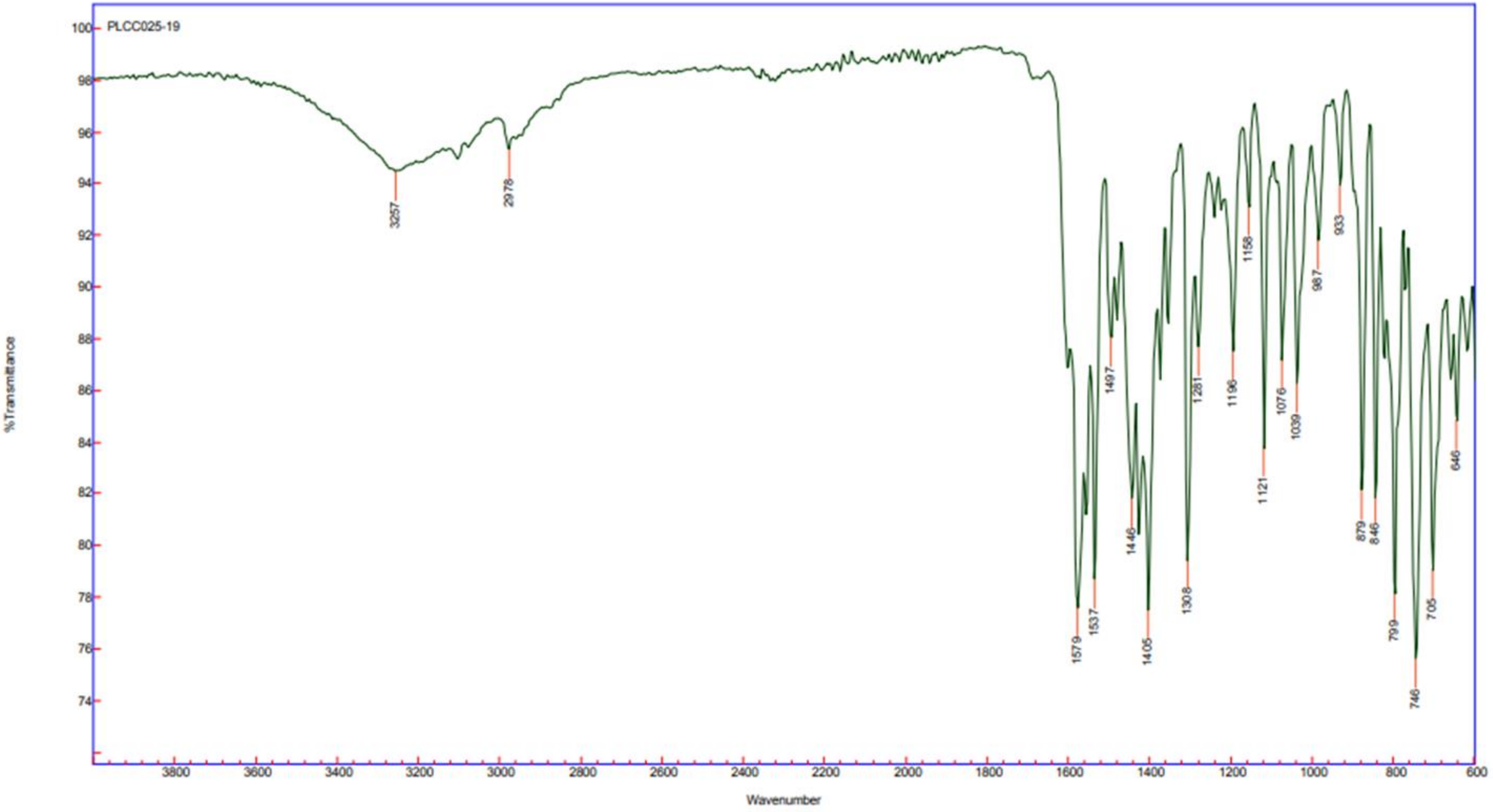


**Figure S47.** IR of **3b**

**Acquisition Parameter**

| | | | | | |
|---|---|---|---|---|---|
| Source Type | ESI | Ion Polarity | Positive | Set Nebulizer | 0.4 Bar |
| Focus | Not active | Set Capillary | 4000 V | Set Dry Heater | 200 °C |
| Scan Begin | 80 m/z | Set End Plate Offset | -400 V | Set Dry Gas | 4.0 l/min |
| Scan End | 1000 m/z | Set Charging Voltage | 2000 V | Set Divert Valve | Source |
| | | Set Corona | 0 nA | Set APCI Heater | 0 °C |

**+MS, 0.2-0.6min #9-32**

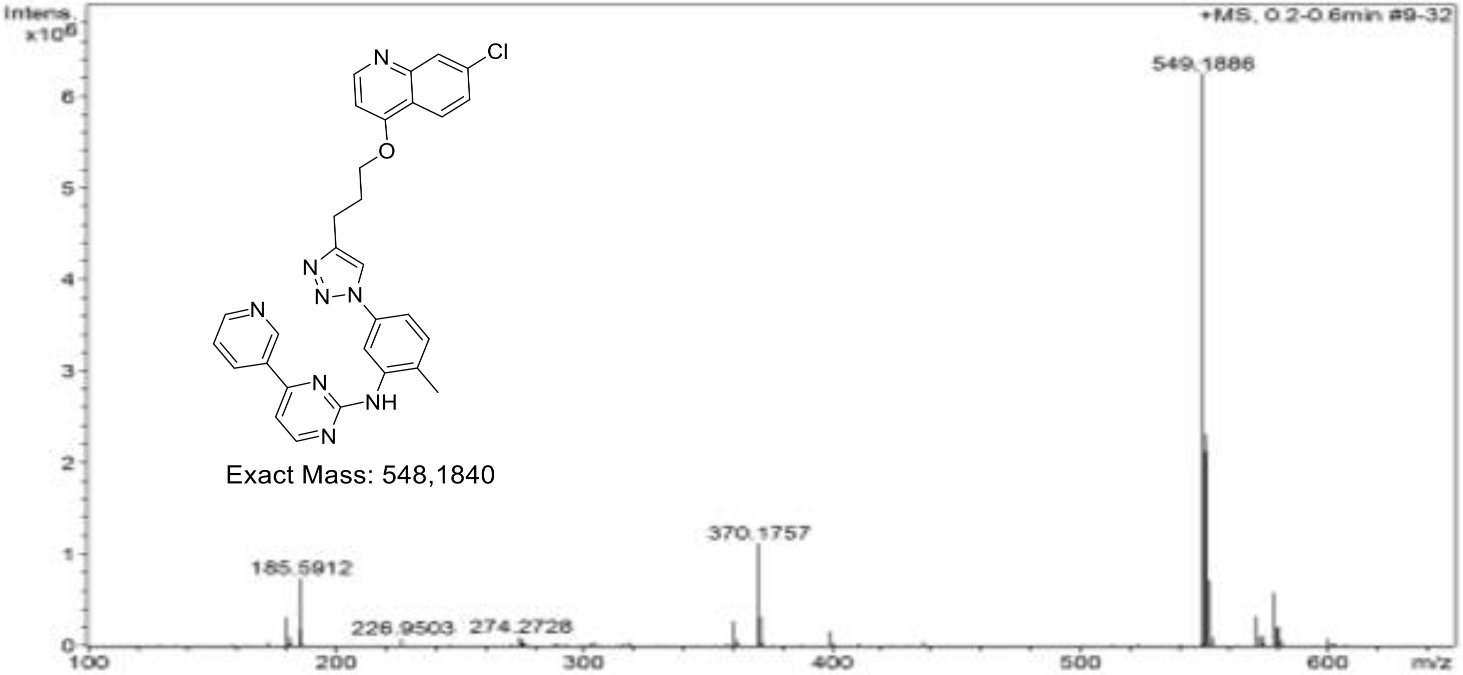


| Meas. m/z | # | Ion Formula | m/z | err [ppm] | Mean err [ppm] | rdb | N-Rule | e⁻ Conf | mSigma | Std I | Std Mean m/z | Std I VarNorm | Std m/z Diff | Std Comb Dev |
|---|---|---|---|---|---|---|---|---|---|---|---|---|---|---|
| 549.188577 | 1 | C30H26ClN8O | 549.191262 | 4.9 | 4.9 | 21.5 | ok | even | 9.7 | 13.0 | n.a. | n.a. | n.a. | n.a. |
| | 2 | C34H30ClN2O3 | 549.193947 | 9.8 | 10.4 | 20.5 | ok | even | 22.1 | 28.7 | n.a. | n.a. | n.a. | n.a. |
| | 3 | C36H26ClN4 | 549.184051 | -8.2 | -7.7 | 25.5 | ok | even | 34.4 | 42.8 | n.a. | n.a. | n.a. | n.a. |

**Figure S48.** HRMS of **3b**

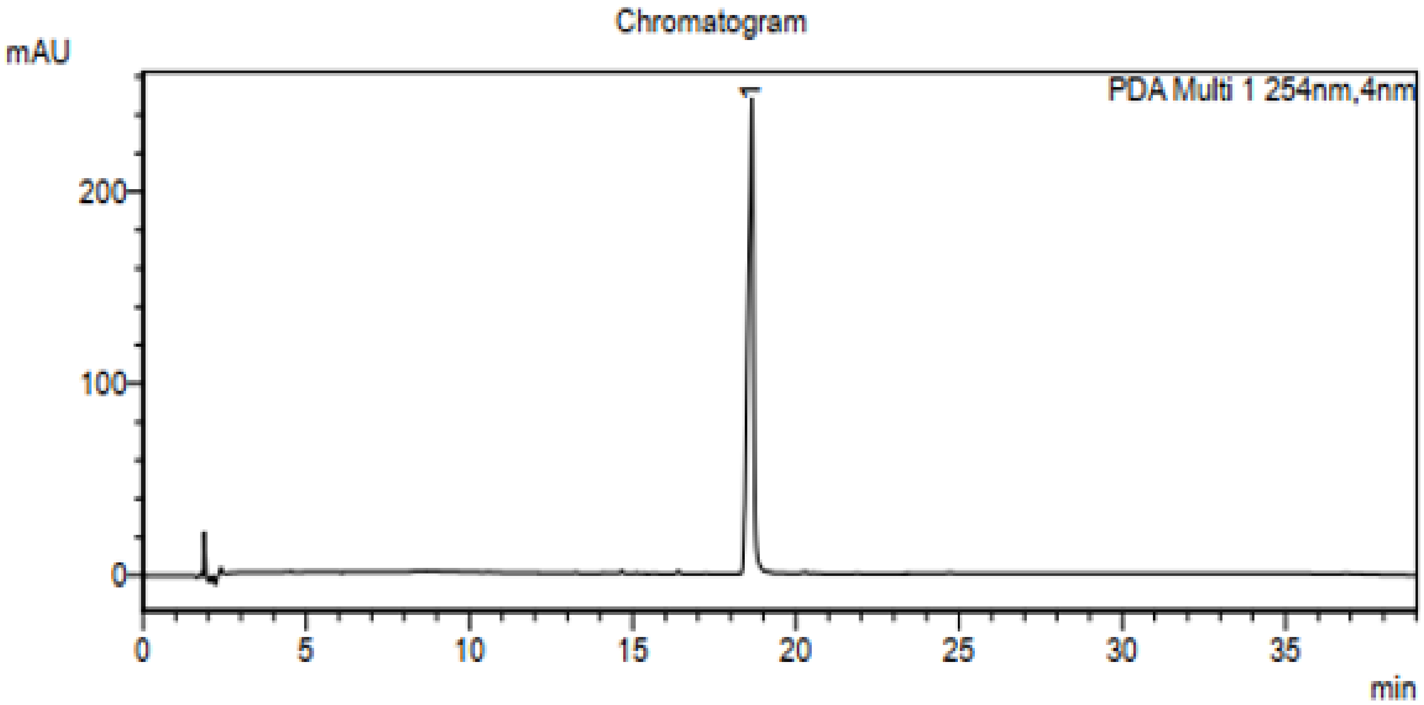


Peak Table

PDA Ch1 254nm

| Peak# | Ret. Time | Name | Area | Area% | Theoretical Plates/meter(USP) | Tailing Factor | Resolution(USP) | Capacity Factor(k') |
|---|---|---|---|---|---|---|---|---|
| 1 | 18,65 | | 2865815 | 100,0 | 346012 | 0,795 | -- | -- |
| Total | | | 2865815 | 100,0 | | | | |

**Figure S49.** HPLC-UV of **3b**

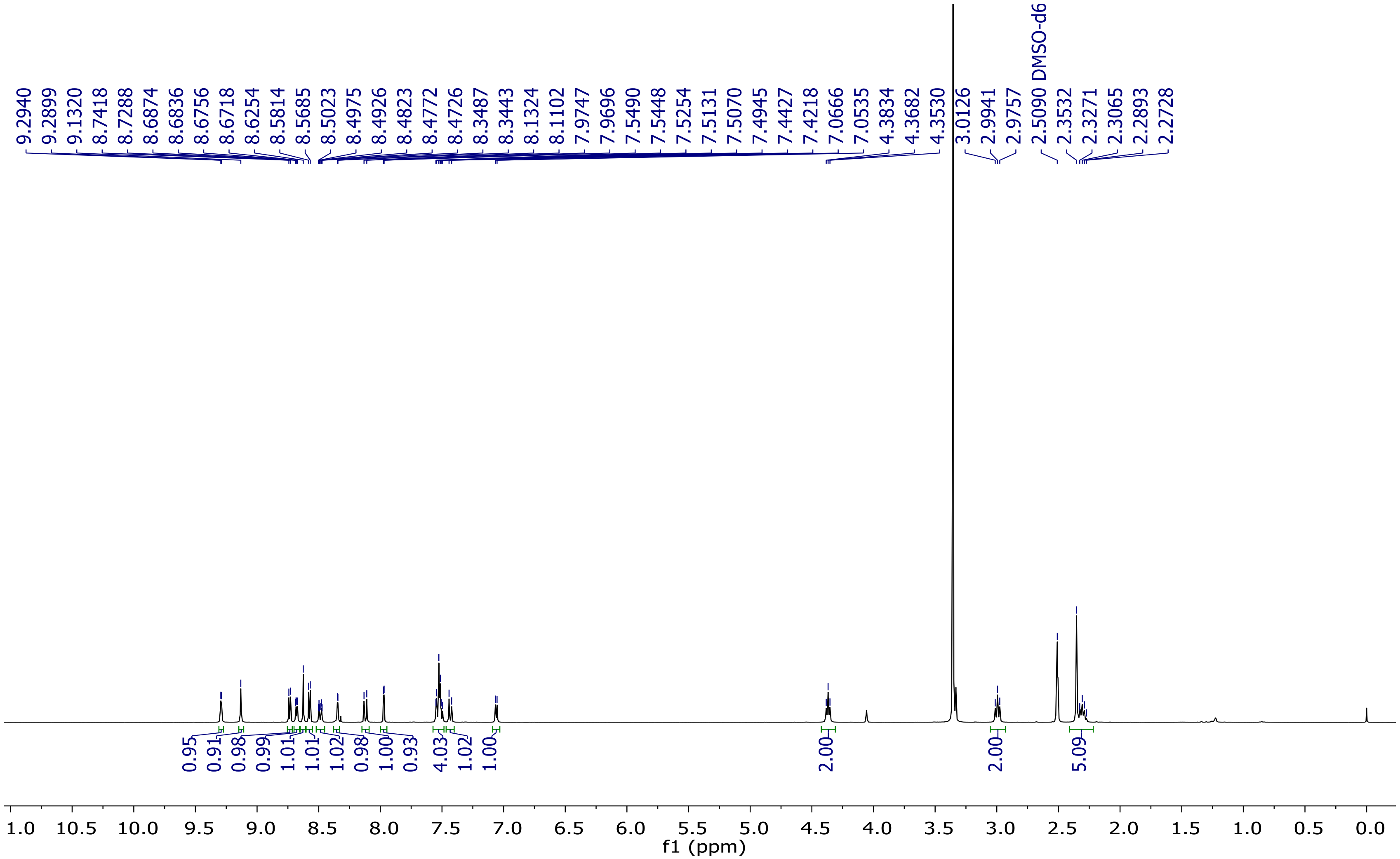


**Figure S50.** $^{1}$H NMR of **3b**

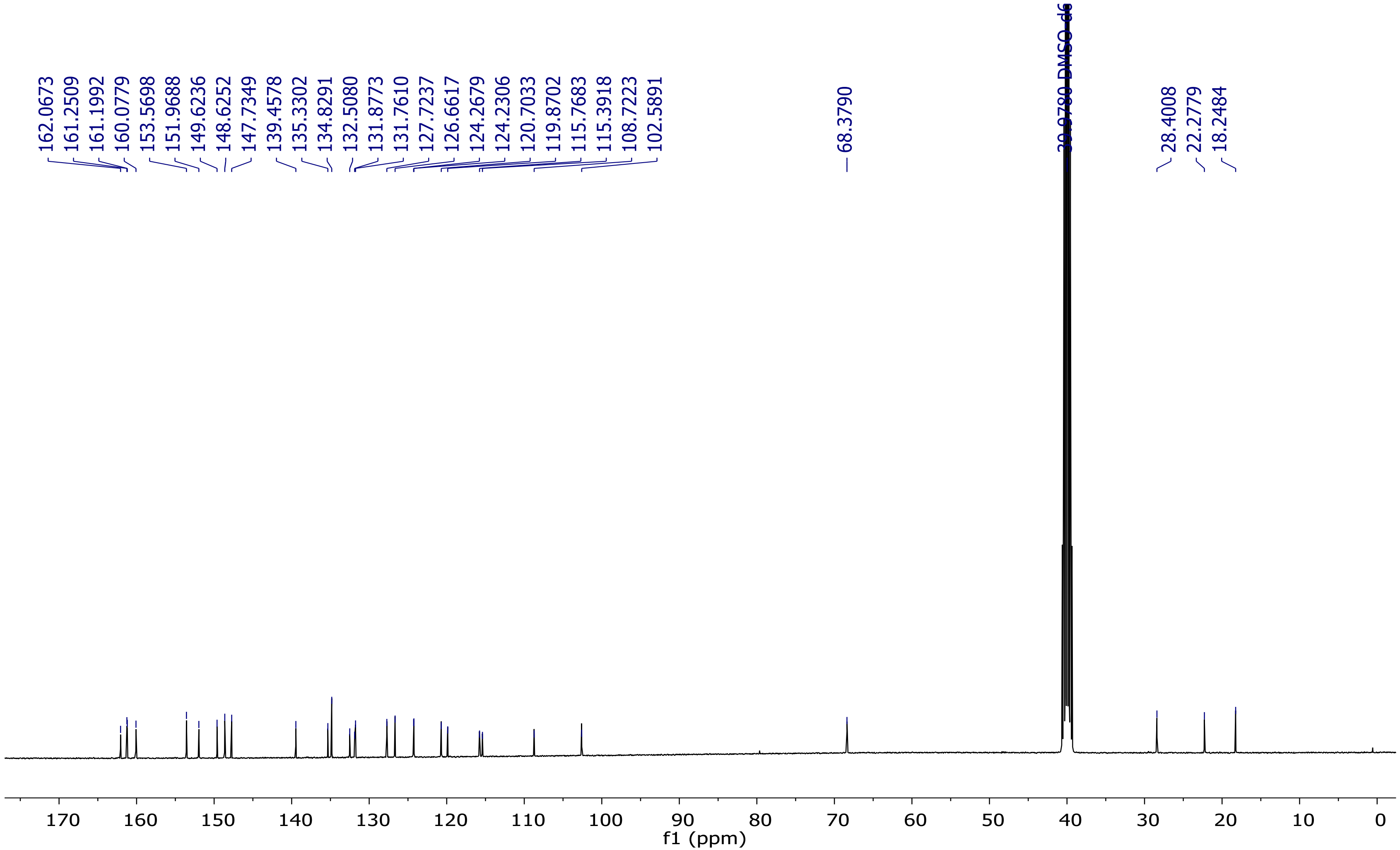


**Figure S51.** $^{13}$C NMR of **3b**

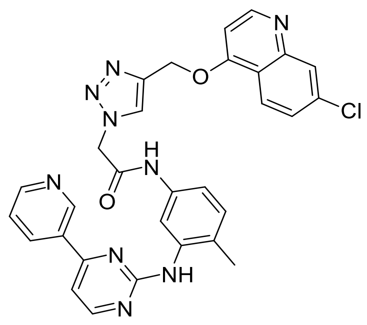

**4a**

2-(4-(((7-chloroquinolin-4-yl)oxy)methyl)-1*H*-1,2,3-triazol-1-yl)-*N*-(4-methyl-3-((4-(pyridin-3-yl)pyrimidin-2-yl)amino)phenyl)acetamide

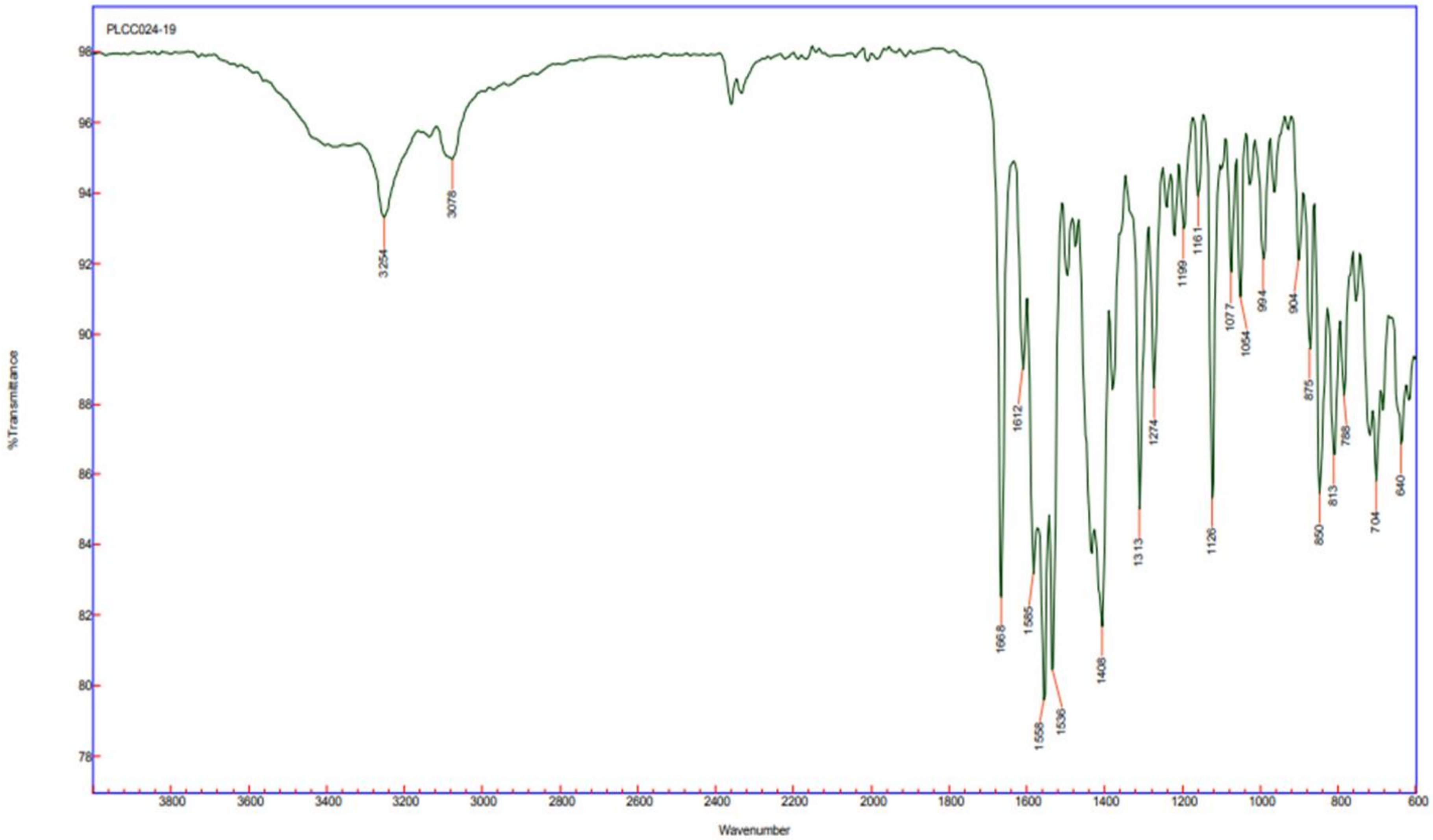


**Figure S52.** IR of **4a**

Comment

**Acquisition Parameter**

| | | | | | |
|---|---|---|---|---|---|
| Source Type | ESI | Ion Polarity | Positive | Set Nebulizer | 0.4 Bar |
| Focus | Not active | Set Capillary | 4000 V | Set Dry Heater | 200 °C |
| Scan Begin | 80 m/z | Set End Plate Offset | -400 V | Set Dry Gas | 4.0 l/min |
| Scan End | 1000 m/z | Set Charging Voltage | 2000 V | Set Divert Valve | Source |
| | | Set Corona | 0 nA | Set APCI Heater | 0 °C |

**+MS, 0.1-0.5min #5-27**

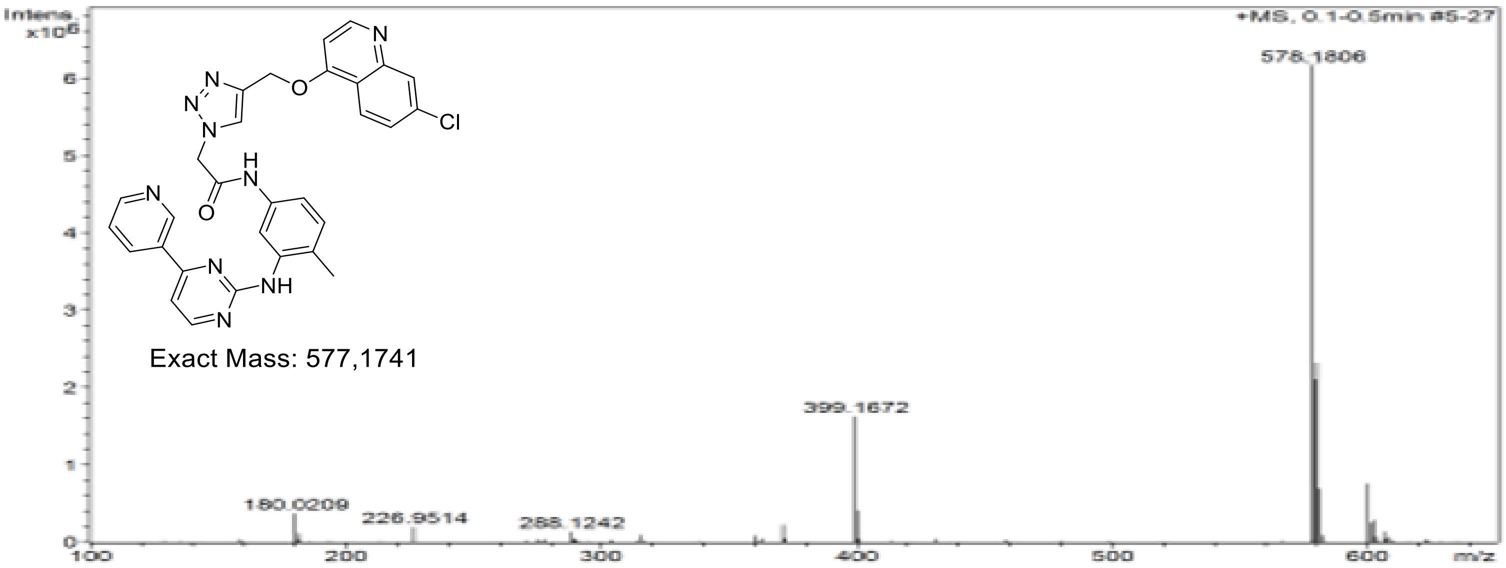


| Meas. m/z | # | Ion Formula | m/z | err [ppm] | Mean err [ppm] | rdb | N-Rule | e⁻ Conf | mSigma | Std I | Std Mean m/z | Std I VarNorm | Std m/z Diff | Std Comb Dev |
|---|---|---|---|---|---|---|---|---|---|---|---|---|---|---|
| 578.180594 | 1 | C30H25ClN9O2 | 578.181425 | 1.4 | 1.7 | 22.5 | ok | even | 10.6 | 14.6 | n.a. | n.a. | n.a. | n.a. |

**Figure S53.** HRMS of **4a**

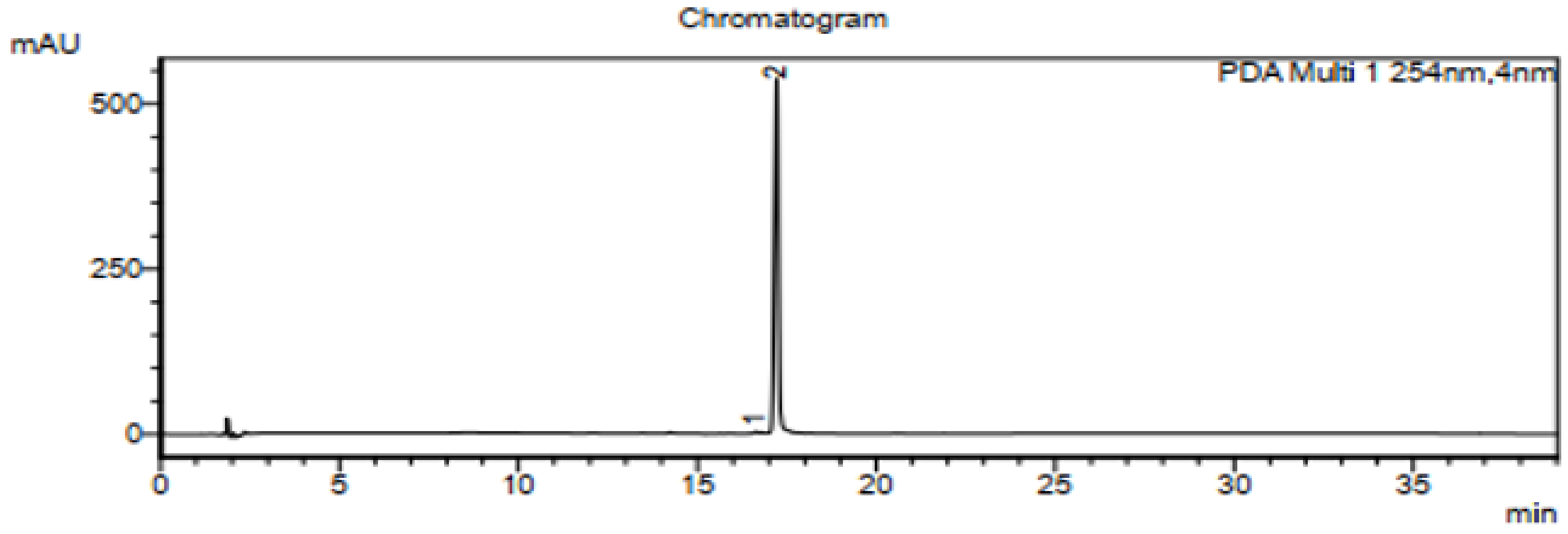


Peak Table

PDA Ch1 254nm

| Peak# | Ret. Time | Name | Area | Area% | Theoretical Plates/meter(USP) | Tailing Factor | Resolution(USP) | Capacity Factor(k') |
|---|---|---|---|---|---|---|---|---|
| 1 | 16,65 | | 23646 | 0,6 | 751290 | -- | -- | -- |
| 2 | 17,23 | | 4171933 | 99,4 | 637565 | 0,912 | 2,759 | 0,035 |
| Total | | | 4195579 | 100,0 | | | | |

**Figure S54.** HPLC-UV of **4a**

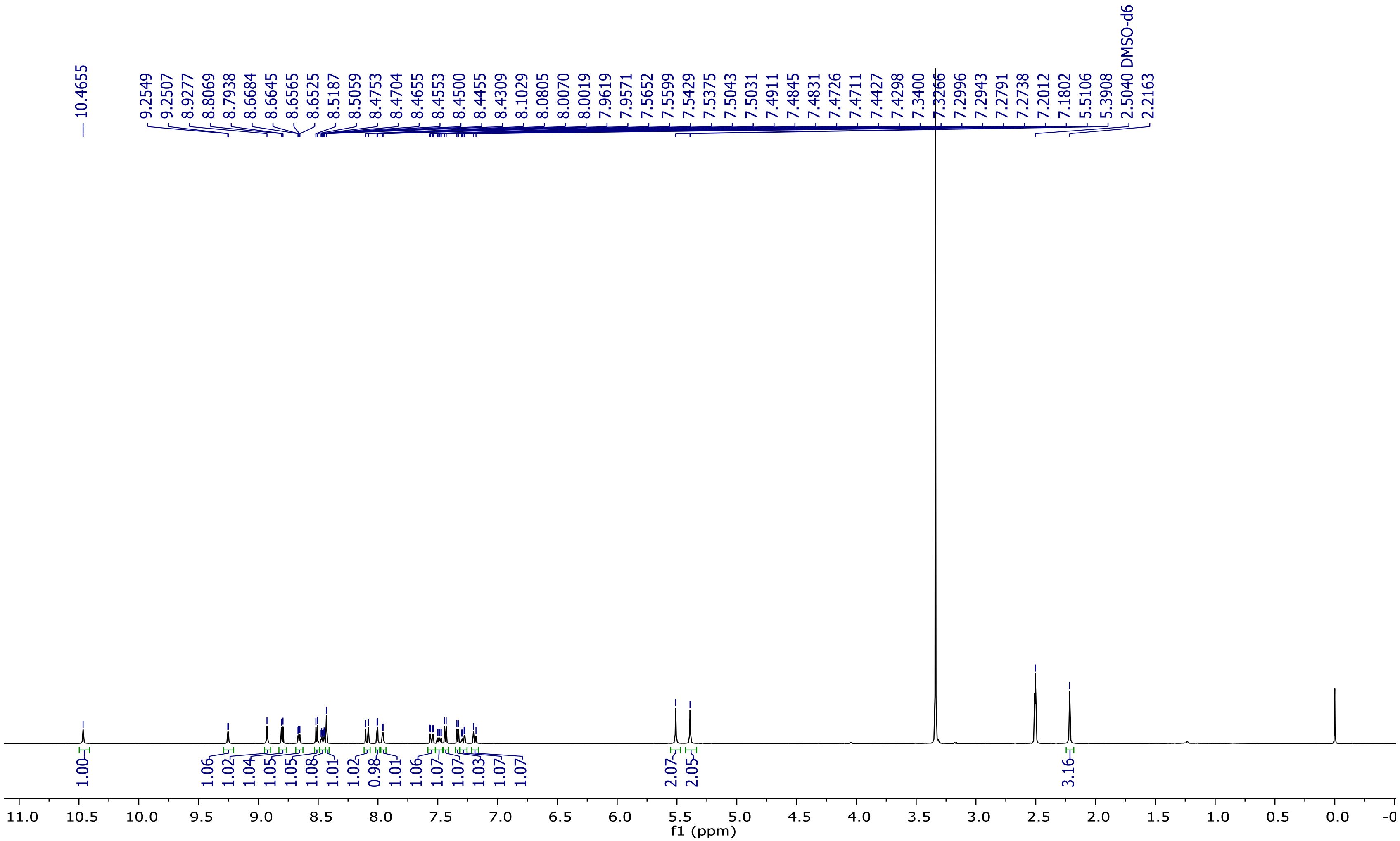

**Figure S55.** $^1$H NMR of **4a**

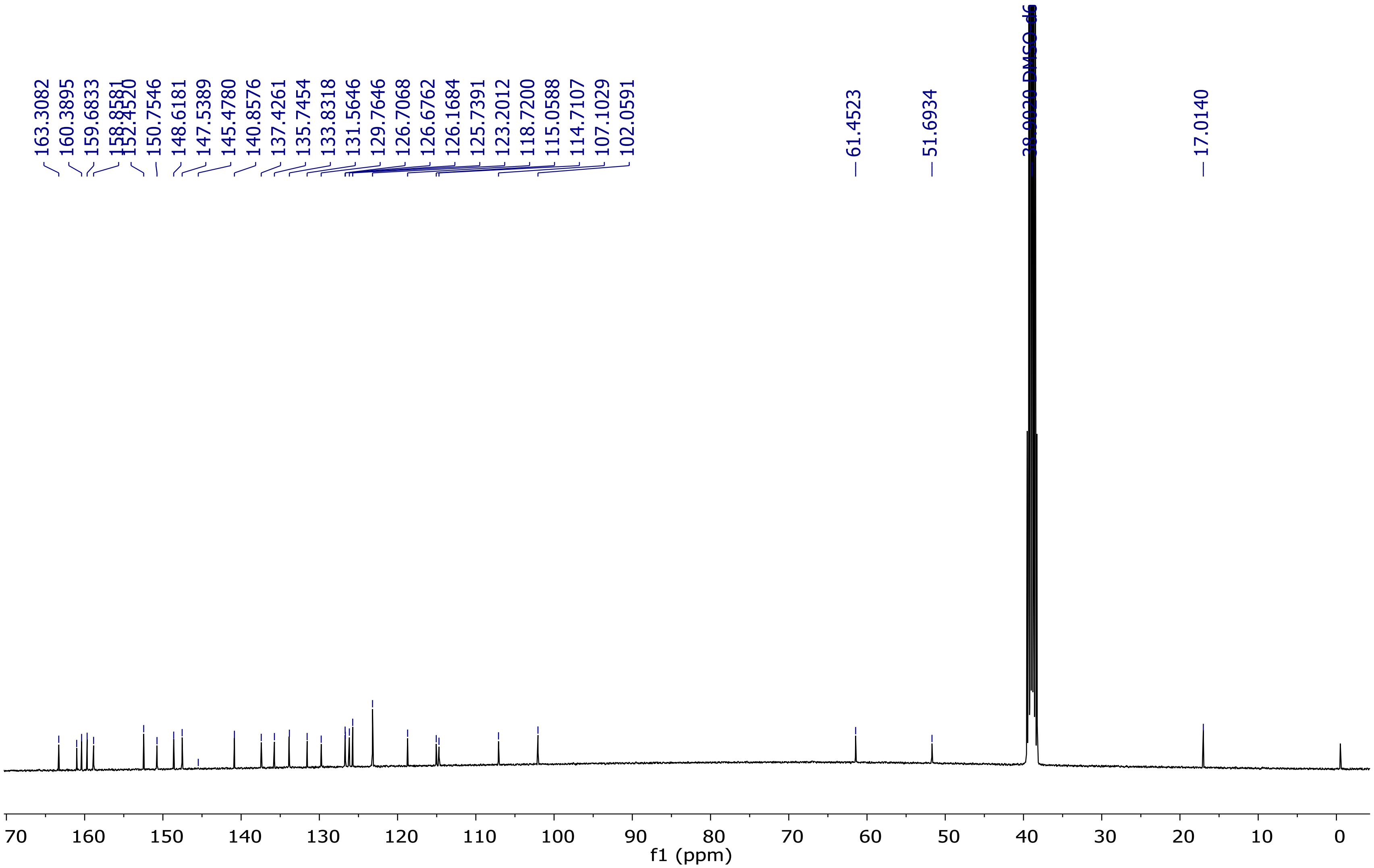


**Figure S56.** $^{13}$C NMR of **4a**

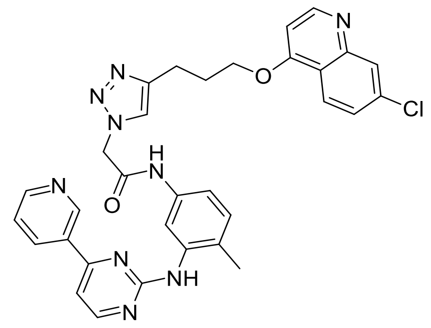

**4b**

2-(4-(3-((7-chloroquinolin-4-yl)oxy)propyl)-1*H*-1,2,3-triazol-1-yl)-*N*-(4-methyl-3-((4-(pyridin-3-yl)pyrimidin-2-yl)amino)phenyl)acetamide

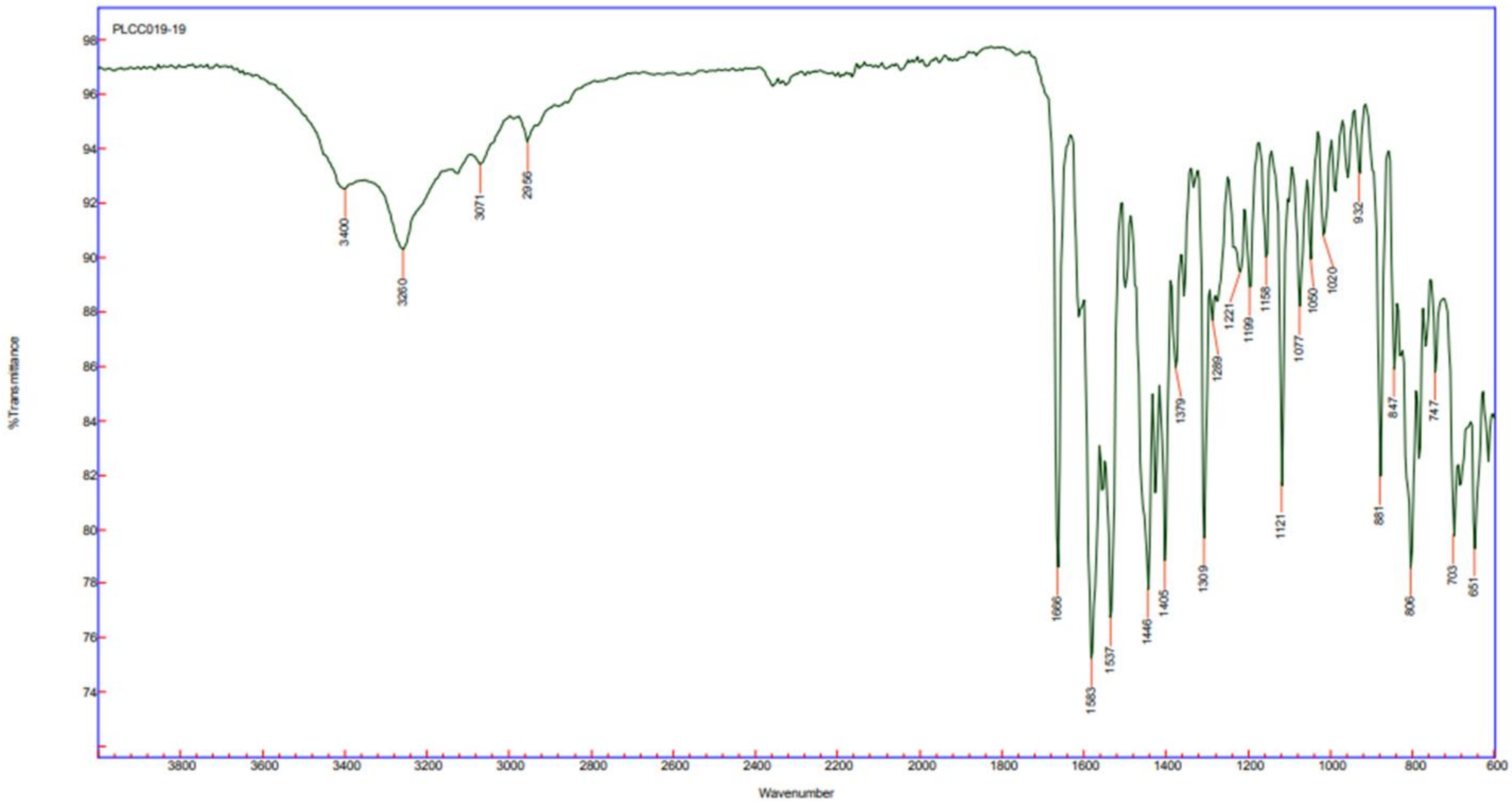


**Figure S57.** IR of **4b**

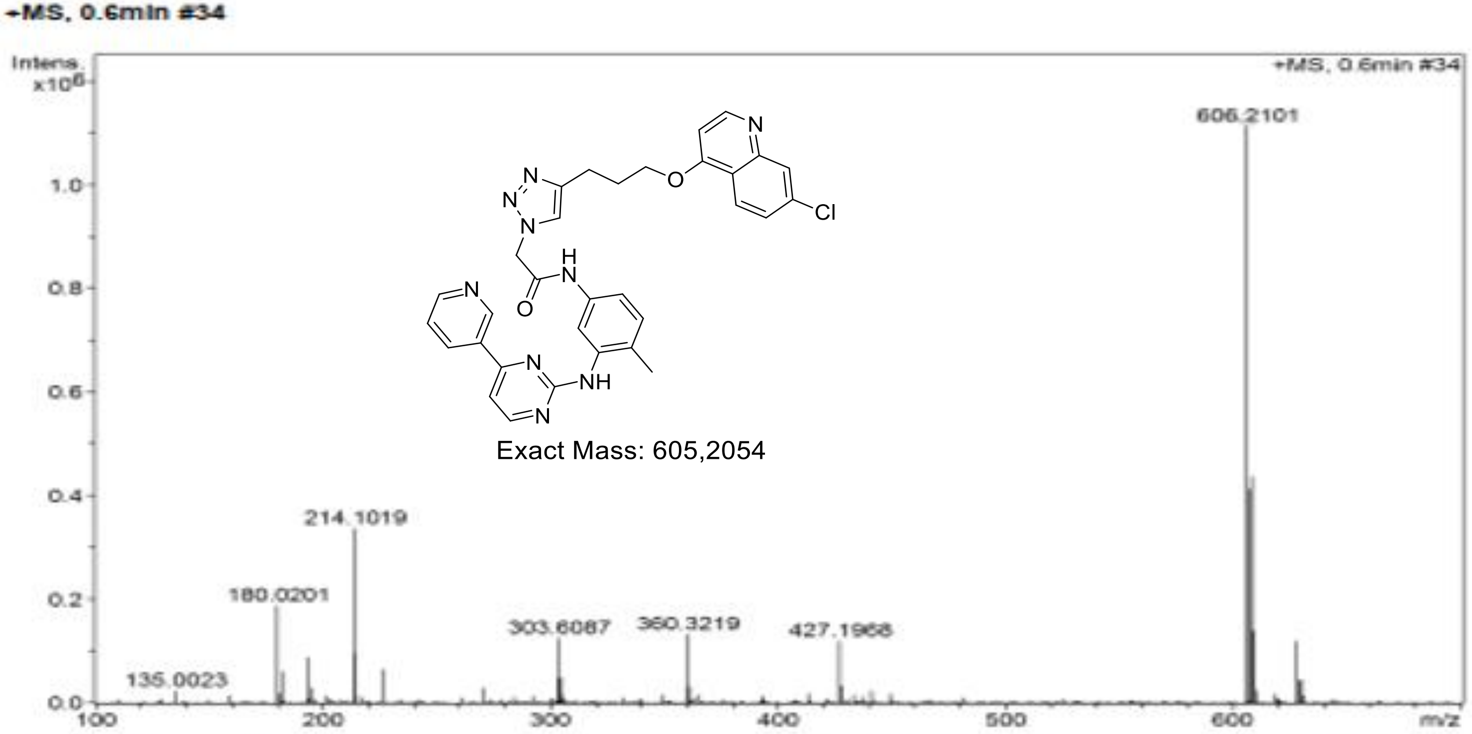


| Meas. m/z | # | Ion Formula | m/z | err [ppm] | Mean err [ppm] | rdb | N-Rule | e⁻ Conf | mSigma | Std I | Std Mean m/z | Std I VarNorm | Std m/z Diff | Std Comb Dev |
|---|---|---|---|---|---|---|---|---|---|---|---|---|---|---|
| 606.210133 | 1 | $C_{32}H_{29}ClN_9O_2$ | 606.212725 | 4.3 | 5.0 | 22.5 | ok | even | 5.6 | 7.1 | n.a. | n.a. | n.a. | n.a. |
| | 2 | $C_{31}H_{33}ClN_5O_6$ | 606.211388 | 2.1 | 3.0 | 17.5 | ok | even | 5.9 | 6.7 | n.a. | n.a. | n.a. | n.a. |
| | 3 | $C_{28}H_{25}ClN_{15}$ | 606.210040 | -0.2 | 0.0 | 23.5 | ok | even | 7.6 | 9.3 | n.a. | n.a. | n.a. | n.a. |
| | 4 | $C_{30}H_{37}ClNO_{10}$ | 606.210050 | -0.1 | 1.1 | 12.5 | ok | even | 16.5 | 19.6 | n.a. | n.a. | n.a. | n.a. |
| | 5 | $C_{27}H_{29}ClN_{11}O_4$ | 606.208703 | -2.4 | -1.9 | 18.5 | ok | even | 17.2 | 21.2 | n.a. | n.a. | n.a. | n.a. |
| | 6 | $C_{26}H_{33}ClN_7O_8$ | 606.207365 | -4.6 | -3.9 | 13.5 | ok | even | 27.7 | 34.6 | n.a. | n.a. | n.a. | n.a. |

**Figure S58.** HRMS of **4b**

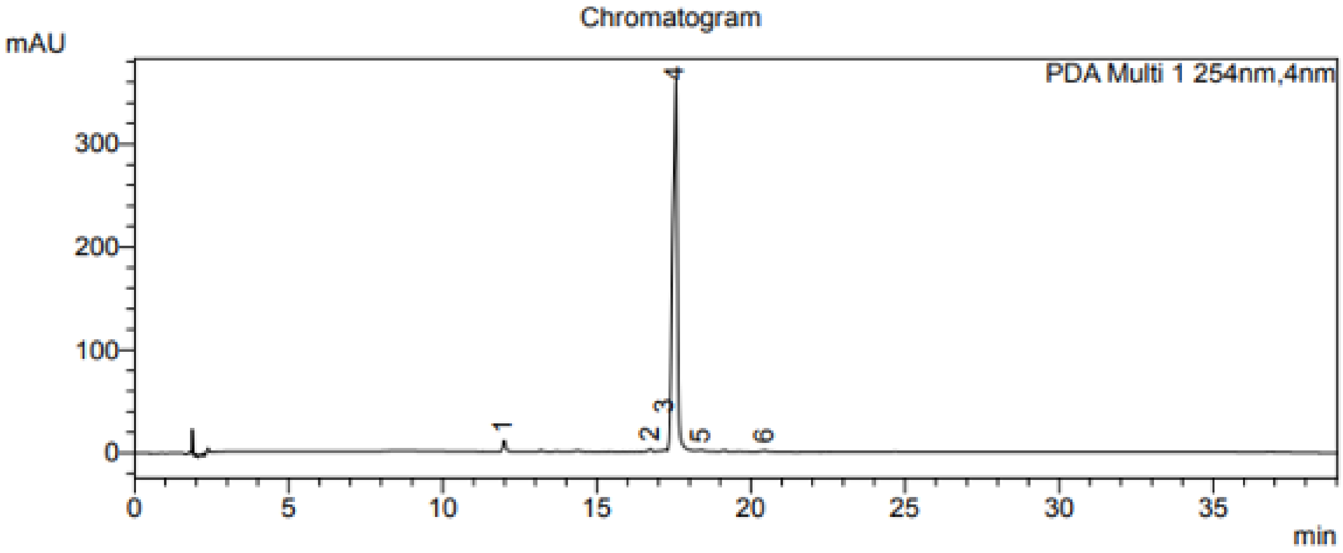


Peak Table

PDA Ch1 254nm

| Peak# | Ret. Time | Name | Area | Area% | Theoretical Plates/meter(USP) | Tailing Factor | Resolution(USP) | Capacity Factor(k') |
|---|---|---|---|---|---|---|---|---|
| 1 | 11,99 | | 71384 | 1,8 | 425726 | 1,302 | -- | -- |
| 2 | 16,75 | | 21218 | 0,5 | 353958 | 0,786 | 19,836 | 0,398 |
| 3 | 17,22 | | 15719 | 0,4 | 544355 | -- | 1,771 | 0,437 |
| 4 | 17,56 | | 3787808 | 96,3 | 374281 | 0,817 | 1,268 | 0,465 |
| 5 | 18,40 | | 21859 | 0,6 | 133088 | -- | 2,052 | 0,535 |
| 6 | 20,45 | | 15991 | 0,4 | 364347 | 0,836 | 4,708 | 0,706 |
| Total | | | 3933980 | 100,0 | | | | |

**Figure S59.** HPLC-UV of **4b**

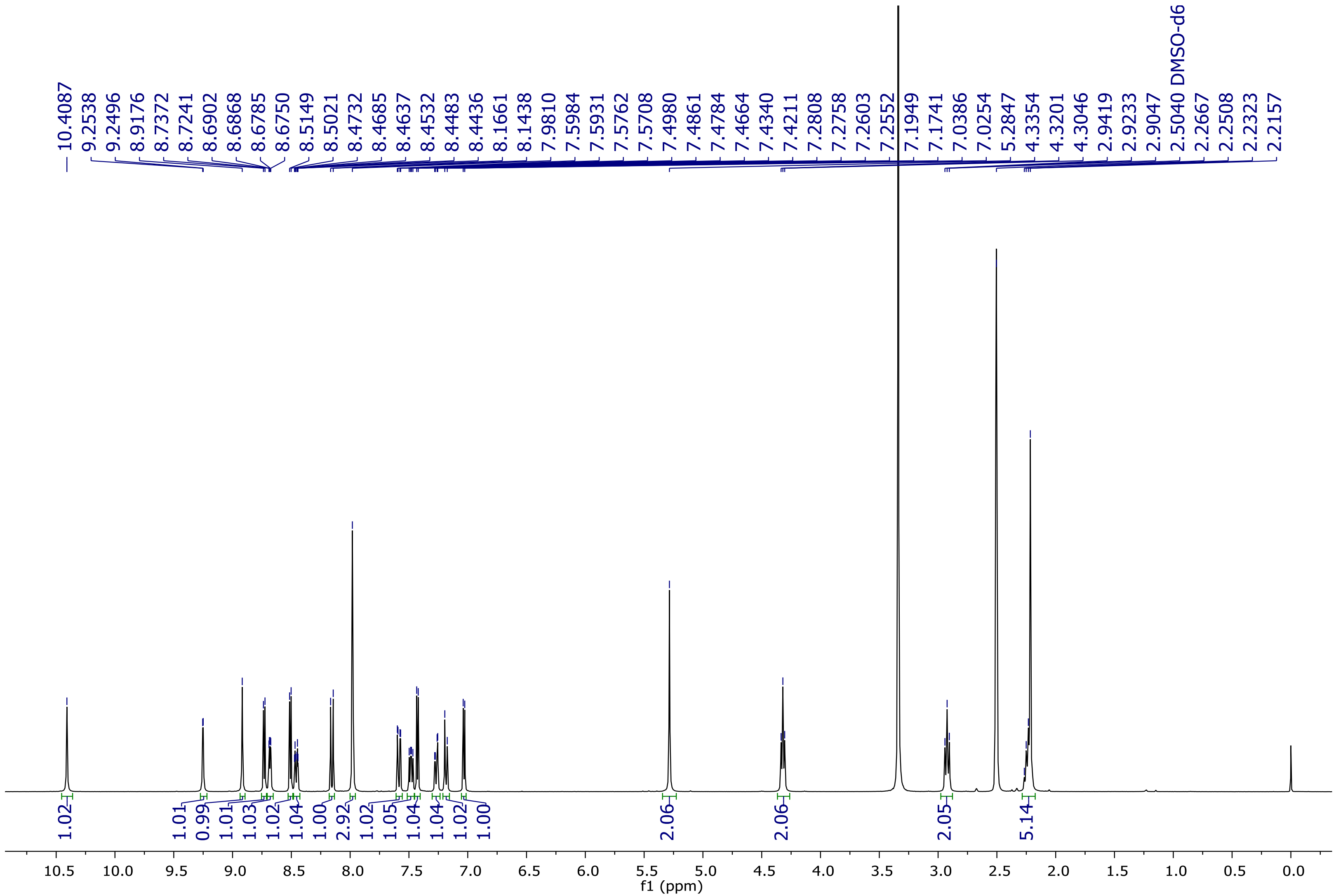


**Figure S60.** $^{1}$H NMR of **4b**

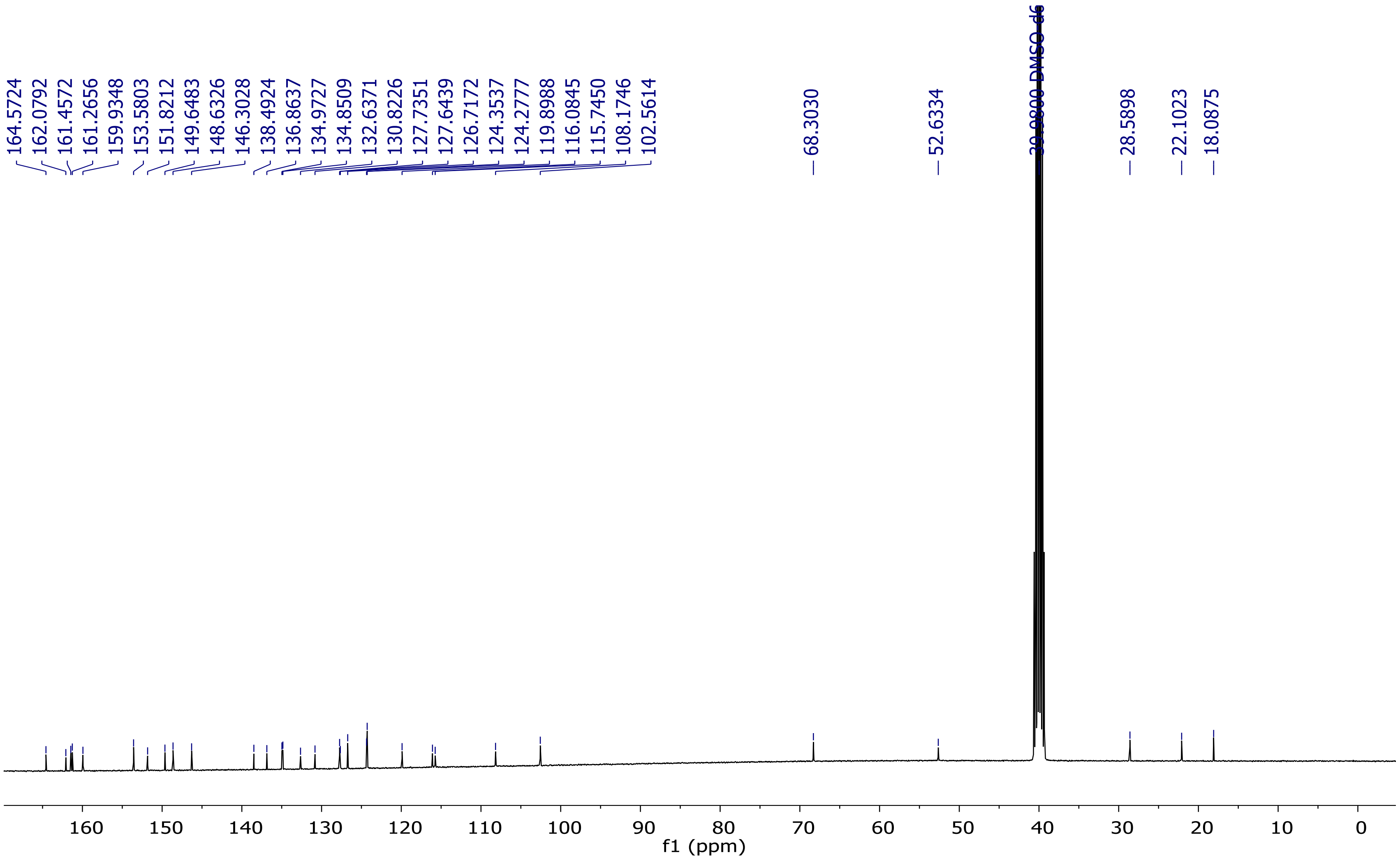


**Figure S61.** $^{13}C$ NMR of **4b**
